# Identification and characterization of the proteolytic flagellin from the common freshwater bacterium *Hylemonella gracilis*

**DOI:** 10.1038/s41598-020-76010-8

**Published:** 2020-11-04

**Authors:** Ulrich Eckhard, Constantin Blöchl, Benjamin G. L. Jenkins, Michael J. Mansfield, Christian G. Huber, Andrew C. Doxey, Hans Brandstetter

**Affiliations:** 1 Department of Biosciences, University of Salzburg, Hellbrunner Straße 34, 5020 Salzburg, Austria; 2 Proteolysis Lab, Department of Structural Biology, Molecular Biology Institute of Barcelona, CSIC, Barcelona Science Park, Baldiri Reixac, 15-21, 08028 Barcelona, Catalonia Spain; 3 Department of Biology, University of Waterloo, 200 University Ave. West, Waterloo, ON N2L 3G1 Canada; 4 Christian Doppler Laboratory for Innovative Tools for Biosimilar Characterization, University of Salzburg, Hellbrunner Straße 34, 5020 Salzburg, Austria; 5 Present Address: Genomics and Regulatory Sytems Unit, Okinawa Institute of Science and Technology Graduate University, Onna, Okinawa, 904-0495 Japan

**Keywords:** Proteases, Proteolysis, Proteomics, Microbiology

## Abstract

Flagellins are the protein components of bacterial flagella and assemble in up to 20,000 copies to form extracellular flagellar filaments. An unusual family of flagellins was recently discovered that contains a unique metalloprotease domain within its surface-exposed hypervariable region. To date, these proteolytic flagellins (also termed flagellinolysins) have only been characterized in the Gram-positive organism *Clostridium haemolyticum*, where flagellinolysin was shown to be proteolytically active and capable of cleaving extracellular protein substrates. The biological function of flagellinolysin and its activity in other organisms, however, remain unclear. Here, using molecular biochemistry and proteomics, we have performed an initial characterization of a novel flagellinolysin identified from *Hylemonella gracilis*, a Gram-negative organism originally isolated from pond water. We demonstrate that *H. gracilis* flagellinolysin (HgrFlaMP) is an active calcium-dependent zinc metallopeptidase and characterize its cleavage specificity profile using both trypsin and GluC-derived peptide libraries and protein substrates. Based on high-throughput degradomic assays, HgrFlaMP cleaved 784 unique peptides and displayed a cleavage site specificity similar to flagellinolysin from *C. haemolyticum.* Additionally, by using a set of six protein substrates, we identified 206 protein-embedded cleavage sites, further refining the substrate preference of HgrFlaMP, which is dominated by large hydrophobic amino acids in P1′, and small hydrophobic or medium-sized polar residues on the amino-terminal side of the scissile bond. Intriguingly, recombinant HgrFlaMP was also capable of cleaving full-length flagellins from another species, suggesting its potential involvement in interbacterial interactions. Our study reports the first experimentally characterized proteolytic flagellin in a Gram-negative organism, and provides new insights into flagellum-mediated enzymatic activity.

## Introduction

Proteolytic flagellins, also known as flagellinolysins, are a recently discovered, enzymatically active class of bacterial flagellins that contain a unique zinc metallopeptidase domain within their hypervariable region (HVR). As demonstrated in the animal pathogen *Clostridium haemolyticum*^1^, flagellinolysins are produced as surface-exposed components of flagellar filaments, and provide flagella with proteolytic activity against external substrates. Currently, 265 putative flagellinolysins (FlaMPs) have been bioinformatically identified across 194 species from the NCBI database, spanning both Gram-positive and Gram-negative bacteria (https://doxey.uwaterloo.ca/flagellinolysin-DB/; accessed 27-05-2020), and form a distinct family (M101) within the metallopeptidases in the MEROPS database^2^. To date, the only studies of flagellinolysin have been in the Gram-positive organism *C. haemolyticum*. Thus, the enzymatic activities and physiological functions of flagellinolysins are still unclear.

The bacterial flagellum is generated by the successive addition of flagellin monomers, which translocate through the central channel of the growing flagellum by the flagellar type III secretion system, generating a gigantic helical assembly of approximately 20,000 protein subunits^3,4^. The prototypic structural flagellin FliC from *Salmonella typhimurium* harbors two non-enzymatic HVR domains of ill-defined function^5^. Notably, removal of the corresponding domains in the *Escherichia coli* flagellin has no significant effect on filament formation^6^, suggesting that HVR-embedded domains may modulate surface properties of flagellar filaments without affecting core filament structure. This is possible due to the three-dimensional structure of flagellins where the conserved N and C terminal regions form together the D0 and D1 domains of the filament core, while the central HVR domain is surface exposed^5^. Consistent with this idea, the filament-forming N- and C-terminal domains of flagellins are extremely conserved across bacteria, but there is considerable variation in the sequence and length of flagellins HVRs, and clear evidence of diversifying selection at the HVR sequence level^7,8^. For instance, a recent study identified a new class of “giant flagellins”, encoding large HVR regions comprising over 1000 residues. These giant flagellins were shown to be abundant in marine Gammaproteobacteria, in which they produce thick flagellar filaments and may represent a mechanical adaptation to their environment^9^.

Importantly, the genomes of about 45% of all flagellated species encode more than one flagellin gene^10^. The production of multiple flagellin variants may diversify the structural and biophysical properties of flagellar filaments, which is consistent with findings that different flagellins show distinct patterns of filament localization^10–15^. *C. haemolyticum* is one such organism that produces multiple types of flagellin, including a structural flagellin (FliC, UniProt ID A0A0A0IUA0), and flagellinolysin (UniProt ID A0A0A0IT63). In purified flagellar filaments of *C. haemolyticum*, it was shown that FliC forms the major filament protein, while flagellinolysin is a less abundant component that is non-specifically distributed throughout the filament^1^.

Flagella not only function in motility, but also play critical roles in a growing variety of processes including biofilm development^16,17^, mechanosensing, host adhesion and invasion during bacterial pathogenesis, and secretion of virulence factors^18–21^. Considering the wide diversity of functions associated with secreted bacterial proteases, this opens a wide avenue of potential roles for flagella-embedded proteolysis. Flagellinolysins may provide additional nutrition by degrading extracellular proteins into peptides, which are subsequently taken up by peptide permeases^22^. Since the permease DppA represents the sensor for the chemotactic-signal transducer TAP in peptide chemotaxis^23^, it is possible that flagellinolysins may generate extracellular peptide gradients to guide the bacterium towards favorable protein-rich environments. Alternatively, flagellinolysins may represent potent toxins in bacterial warfare by attacking cell components of competing species^24^, and/or the extracellular polymeric substances of biofilms^25,26^. Similarly, proteolytic flagellins could aid host invasion, colonization and toxin diffusion by degrading the extracellular matrix, similar to what has been shown for bacterial collagenases^27–29^, either embedded in the filament or after secretion, as seen for example in *Campylobacter jejuni* for the non-enzymatic flagellin FlaC^30^.

To gain further insights into flagellinolysin function, we investigated the predicted flagellinolysin gene product from the Gram-negative bacterium *Hylemonella gracilis*, a common microbe in freshwater ecosystems throughout the world. *H. gracilis* forms motile spirilla of small diameter (0.2 to 0.3 µm), and is notable for its ability to pass through 0.1 µm sterilizing grade filters^31^. It produces bipolar tufts of flagella, and is classified within the Burkholderiales family of Betaproteobacteria^32,33^. Cultures are urease positive, a prominent virulence factor in *Helicobacter pylori*^34^, but cannot form spores or liquefy gelatin^33^. Interestingly, it has been reported that *H. gracilis* can prevent the long-term persistence of the plague-causing bacteria *Yersinia pestis* in filtered fresh water, suggesting bactericidal activity. And moreover, *Y. pestis* appears to condition its competitor for outgrowth, and allows *H. gracilis* to thrive on otherwise unsuitable media^35^.

Here, using molecular biochemistry and proteomics, we performed an in-depth proteolytic profiling of the flagellinolysin (flagellin metallopeptidase, FlaMP) from *H. gracilis*, herein referred to as HgrFlaMP. We demonstrate that HgrFlaMP is an active metalloprotease, and report cleavage specificity profiles against both peptide libraries and protein substrates. Our work establishes HgrFlaMP as the second biochemically described proteolytic flagellin to date, expanding our knowledge of this intriguing family of enzymes.

## Results

### Identification of flagellinolysin from the *Hylemonella gracilis* genome

**Figure 1:**
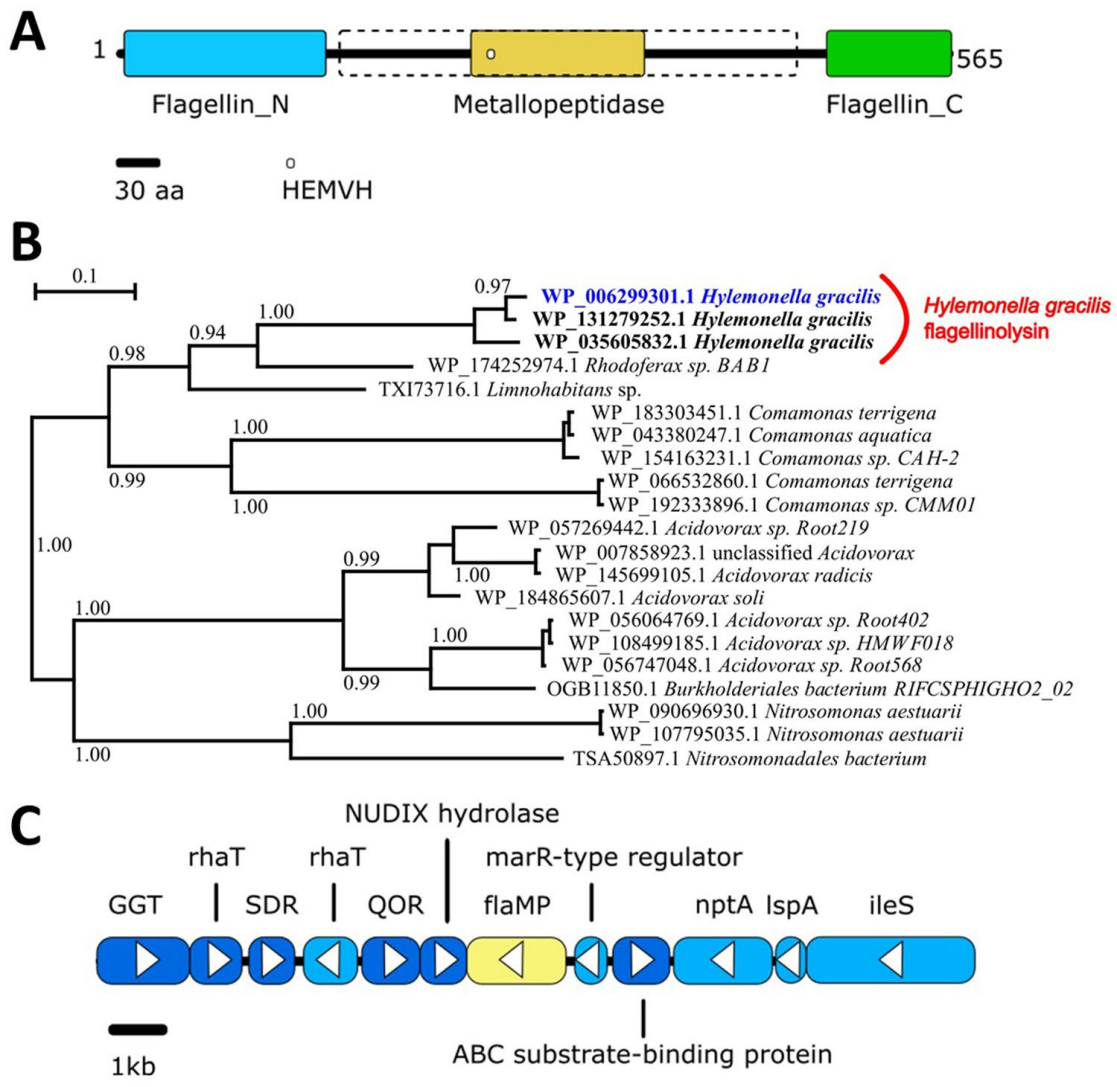
Identification of a putative proteolytic flagellin (flagellinolysin) from *Hylemonella gracilis*. (**A**) Bioinformatically annotated domain architecture of a predicted flagellinolysin from *H. gracilis* ATCC 19624 (UniProt F3KXG2, NCBI accession WP_006299301). The catalytic HExxH motif of zinc-metallopeptidases is indicated as a white circle and is found within the central hypervariable region, which is flanked by the N- and C-terminal flagellin domains. The dashed box indicates the domain boundary used for the biochemical characterization of the HVR-only protein (Leu163 to Ile450). (**B**) Phylogenetic tree of *H. gracilis* flagellinolysin and its top identified homologs by BLASTp from the NCBI database. The phylogenetic tree was generated from a MUSCLE-generated multiple alignment using PhyML with default parameters and an LG model, as implemented within SeaView. (**C**) Genomic context of the *H. gracilis* flagellinolysin gene (labeled as *flaMP* and highlighted in yellow), displaying its surrounding gene neighborhood.

Using the sequence of flagellinolysin from *C. haemolyticum* in a BLAST query against the NCBI and UniProtKB reference database, we identified a putative flagellinolysin protein from the organism, *H. gracilis* ATCC 19624 (UniProt F3KXG2, NCBI accession WP_006299301). The *H. gracilis* and *C. haemolyticum* homologs share 37% amino acid identity and are well-aligned over the zinc-binding HExxH catalytic motif (residues 249–253 and 233–237 respectively) of the metallopeptidase active site. According to the domain annotations from the Conserved Domain Database (CDD)^36^, the *H. gracilis* protein contains the characteristic flagellinolysin architecture consisting of conserved flagellin N-terminal and C-terminal domains, which form the filament core, and a metallopeptidase domain within the central hypervariable region (Fig. 1A). In addition to *H. gracilis* strain ATCC 19624, highly similar proteins (97% and 92% amino acid identity) were also identified in two other sequenced *H. gracilis* strains within the NCBI, which clustered together in a phylogenetic analysis (Fig. 1B). *H. gracilis* flagellinolysins are most closely related to flagellinolysin homologs from related taxa including *Rhodoferax sp*. (68% amino acid identity), *Limnohabitans* sp. (66% amino acid identity) and *Comamonas* sp. (55–56% amino acid identity), which are also in the Comamonadaceae family of bacteria. This suggests that HgrFlaMP has not evolved through a unique domain fusion event within *Hylemonella*, but instead flagellinolysin may be a conserved protein within the Comamonadaceae lineage and that these proteins share common ancestry, which may have originated earlier through lateral gene transfer from a more distantly related organism. Interestingly, while flagellinolysin is encoded within a flagellar gene cluster in the *C. haemolyticum* genome, this is not the case for the gene from *H. gracilis* (Fig. 1C). In all three *H. gracilis* strains, flagellinolysin is encoded nearby non-flagellar genes encoding NUDIX hydrolase^37^, a MarR-type transcriptional regulator^38^, and others. The functional relevance of this genomic context is unclear.

### Protein expression and purification

**Figure 2:**
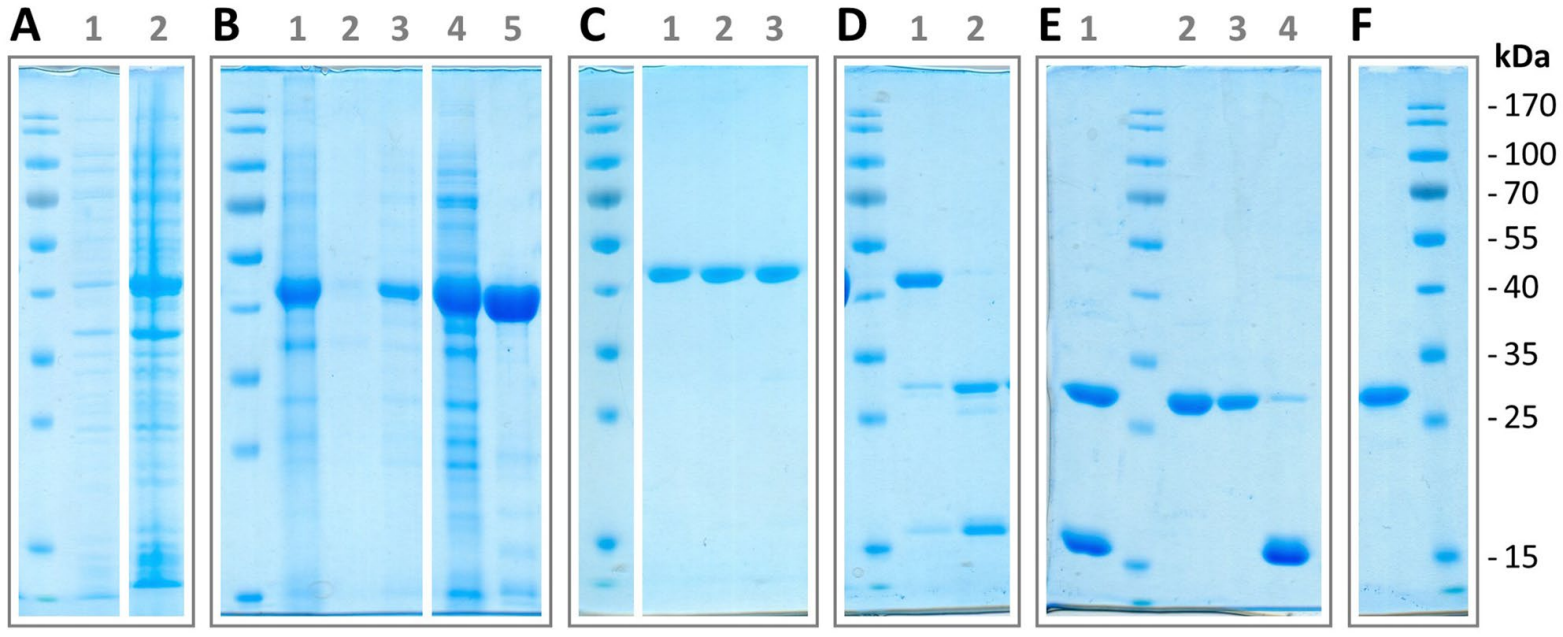
Expression and purification of the HVR-domain of HgrFlaMP. (**A**) Protein expression samples of His6-SUMO tagged recombinant protein before (1) and after (2) induction with IPTG (expected molecular weight: 44 kDa). (**B**) Cell lysis and subsequent NiNTA purification including refolding. 1 total lysate; 2 soluble protein after lysis; 3 insoluble protein in 8 M urea; 4 NiNTA flow-through; 5 NiNTA elution. (**C**) Protein after size-exclusion chromatography; peak fraction and the two surrounding SEC fractions are shown. (**D**) Removal of the N-terminal purification tag (expected molecular weight: 14 kDa) by SUMO protease. Samples right after protease addition (1) and 1 h of incubation (2) are shown. (**E**) NiNTA re-chromatography. 1 recombinant protein after tag removal (expected molecular weight: 30 kDa); 2 NiNTA flow-through and 3 wash containing the target protein; 4 NiNTA elution dominated by the His6-SUMO tag. (**F**) Recombinant tag-depleted HgrFlaMP-HVR after size-exclusion chromatography. Figure panels with lanes from different gels show white separation lines, and all original scans can be found in the supplementary materials.

To examine the biological activity of the putative flagellinolysin from *H. gracilis*, we cloned and expressed the full-length (Met1-Arg565) and HVR-only (Leu163-Ile450) proteins. Both variants yielded inclusion bodies in *Escherichia coli*, and were refolded on-column by decreasing concentrations of urea. A size-exclusion chromatography step right after refolding and before tag-removal with SUMO-protease was crucial to separate folded protein from soluble aggregates, as the latter hindered separation of tagged and untagged protein via NiNTA re-chromatography, likely due to assemblies of mixed species. This was especially a problem for the full-length protein, likely due to the presence of the terminal flagellin D0 domain, which represents the major driver of self-polymerization^39^. However, we have previously demonstrated that both flagella-embedded full-length flagellinolysin from *C. haemolyticum* and its recombinantly-produced HVR-protein show similar proteolytic behavior^1^. Thus, after initial trials with full-length HgrFlaMP, we focused on the HVR-protein as it showed better stability and was better suited for the full purification protocol. The purification process for the HVR protein is shown in Fig. 2, and representative size-exclusion chromatograms are included in Supplementary Figure 1. The partial purification of the full-length protein prior to purification-tag removal is shown in Supplementary Figure 2AB. Importantly, despite solubilization in 8 M urea, not all the recombinant HVR protein was available for NiNTA purification, probably due to soluble protein aggregates and thus inaccessibility of the His6-tag (Fig. 2B). However, reloading of the flow-through after another 24 h of incubation in 8 M urea—but not immediately after the first round of NiNTA—rescued approximately 50% of the remaining target protein. As protein purifications started from rather pure inclusion body preparations, one round of NiNTA and size-exclusion chromatography yielded highly pure and monodisperse target protein, albeit still His6-SUMO-tagged (Fig. 2C, Supplementary Figure 1A). The purification tag was subsequently removed by incubation with His6-tagged SUMO protease, and the tag-depleted protein was purified by final NiNTA and size-exclusion chromatography steps (Fig. 2D–F).

### Biochemical characterization

**Figure 3:**
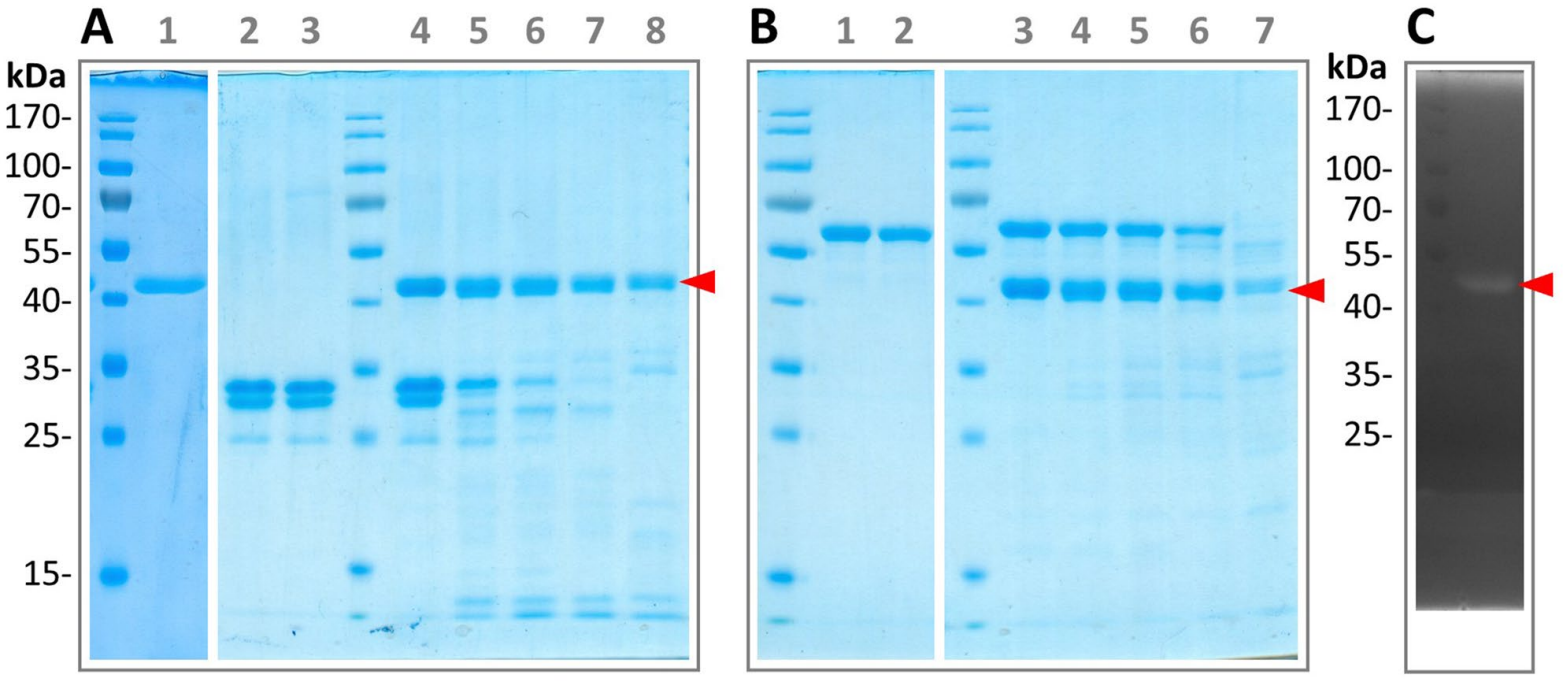
Degradation of bovine casein and the structural flagellin from *P. tunicata* by His6-SUMO-tagged HgrFlaMP-HVR. (**A**) 1 tagged HgrFlaMP after NiNTA and SEC purification (expected molecular weight: 44 kDa). 2–3 bovine casein after 0 and 16 h of incubation at 16 °C (control). 4–8 bovine casein after incubation with HgrFlaMP-HVR for 0, 1, 2, 4, and 16 h, respectively. (**B**) 1–2 structural flagellin from *P. tunicata* (expected molecular weight 61 kDa) after 0 and 16 h of incubation at 16 °C. 3–7 structural flagellin after incubation with HgrFlaMP-HVR for 0, 1, 2, 4, and 16 h, respectively. (**C**) Casein zymography of His6-SUMO-tagged HgrFlaMP-HVR. For clarity, the flagellinolysin band is indicated by a red triangle. Figure panels with lanes from different gels show white separation lines, and all original scans can be found in the supplementary materials.

**Figure 4:**
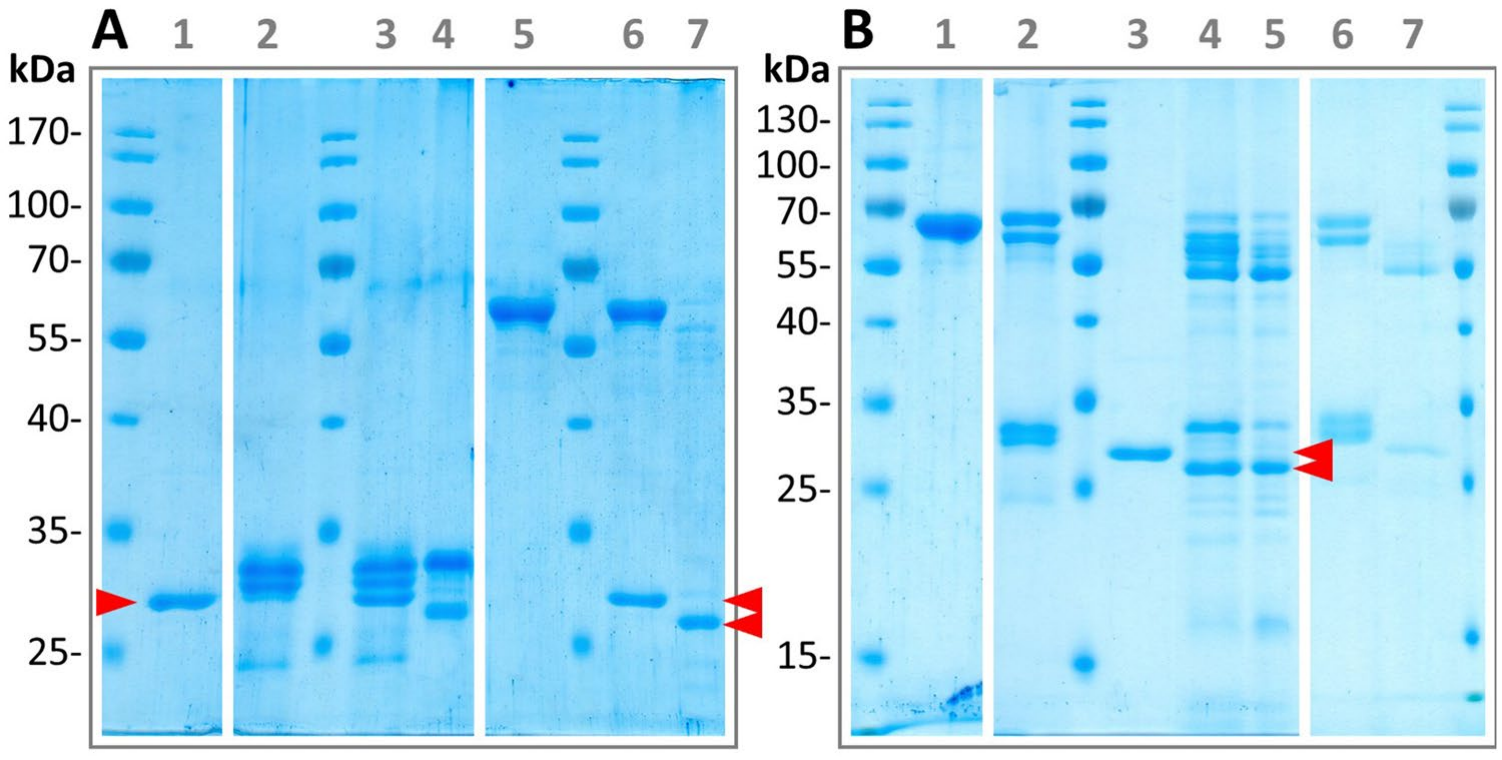
Degradation of bovine casein and the structural and proteolytic flagellin from *P. tunicata* by recombinant HgrFlaMP-HVR. (**A**) 1 tag-depleted HgrFlaMP-HVR (expected molecular weight: 30 kDa). 2 bovine casein. 3–4 bovine casein incubated with HgrFlaMP-HVR in presence of EDTA or Ca^2+^/Zn^2+^, respectively. 5 structural flagellin from *P. tunicata* (expected molecular weight: 61 kDa). 6–7 structural flagellin incubated with flagellinolysin HVR in presence of EDTA or Ca^2+^/Zn^2+^, respectively. (**B**) 1 inactivated HAxxH variant of the full-length proteolytic flagellin from *P. tunicata* (expected molecular weight: 65 kDa). 2 substrate mix consisting of bovine casein and both the structural and proteolytic flagellin from *P. tunicata*. 3 tag-depleted HgrFlaMP-HVR. 4–5 substrate mix after 2 and 4 h of incubation with HgrFlaMP-HVR at 16 °C. 6–7 incubation of substrate mix with flagellinolysin HVR in presence of either 10 mM EDTA or 10 mM Ca^2+^ and 10 µM Zn^2+^ after 2 h at 20 °C. HgrFlaMP-HVR is indicated by a red triangle. Figure panels with lanes from different gels show white separation lines, and all original scans can be found in the supplementary materials.

Initial biochemical characterizations were performed using the His6-SUMO-tagged protein. As initial candidate substrates, we selected bovine casein, given its standard use as a general protease target, as well as flagellins from another bacterial species, given our hypothesis that flagellinolysin may target flagella from other competing cells. While only minor activity was detected for the full-length flagellinolysin against bovine casein (Supplementary Figure 2C), full degradation of both casein and the structural flagellin from *Pseudoalteromonas tunicata* could be observed when using the tagged HVR protein (Fig. 3A,B). The proteolytic activity of HgrFlaMP could be further confirmed by casein zymography (Fig. 3C). Interestingly, the recombinant protein displayed a time- and substrate-dependent tendency of self-digestion. While substrate degradation clearly dominated over self-cleavage in the presence of casein, cleavage of the structural flagellin appeared to be only slightly favored over autolysis. Intriguingly, the purified tag-depleted HgrFlaMP-HVR protein revealed a highly similar—but not identical—proteolytic behavior, probably due to the presence of soluble aggregates next to correctly folded protein in the His-SUMO tagged preparations (Fig. 4). Both bovine casein and the *P. tunicata* structural flagellin were readily digested by the tag-depleted HgrFlaMP-HVR protein, but instead of self-degradation, a shift in molecular weight by 1–2 kDa was observed, consistent with the removal of 10–20 amino acids from the N- or C-terminus. Additionally, a preferential degradation of beta-casein over alpha- and kappa-casein^40^ could be observed (Fig. 4A), and which we later confirmed by mass spectrometry (Supplementary Figures 3–6). We then additionally included inactive full-length flagellinolysin from *P. tunicata* into the proteinaceous substrate mix, which was likewise degraded by HgrFlaMP (Fig. 4B). Both flagellins from *P. tunicata* were expressed and purified as tag-depleted variants as described for HgrFlaMP. Representative size-exclusion chromatograms of the final purification step are shown in Supplementary Figure 7. Importantly, four hours of incubation with HgrFlaMP-HVR at a protease to substrate ratio of 1:25 at 22 °C were sufficient to ensure nearly complete degradation of the provided substrates (Fig. 4B, lanes 6–7). Consistent with our finding for flagellinolysin of *C. haemolyticum*^1^, addition of 10 mM CaCl_2_ and 10 µM ZnCl_2_ were needed to reach full enzymatic activity, while 10 mM EDTA abrogated proteolysis. This further supports the notion of flagellinolysins as zinc- and calcium-dependent metallopeptidases, as bioinformatically predicted due to the remote homology between the flagellinolysin HVR domain and the peptidase domain of clostridial collagenases^1,41^. A multiple sequence alignment of 10 selected flagellinolysins around their active site, and sequence logos of the conserved regions surrounding the zinc-binding HExxH motif and the putative calcium binding aspartate are shown in Supplementary Figure 8.

### Specificity profiling by proteomic identification of protease cleavage sites (PICS)

**Figure 5:**
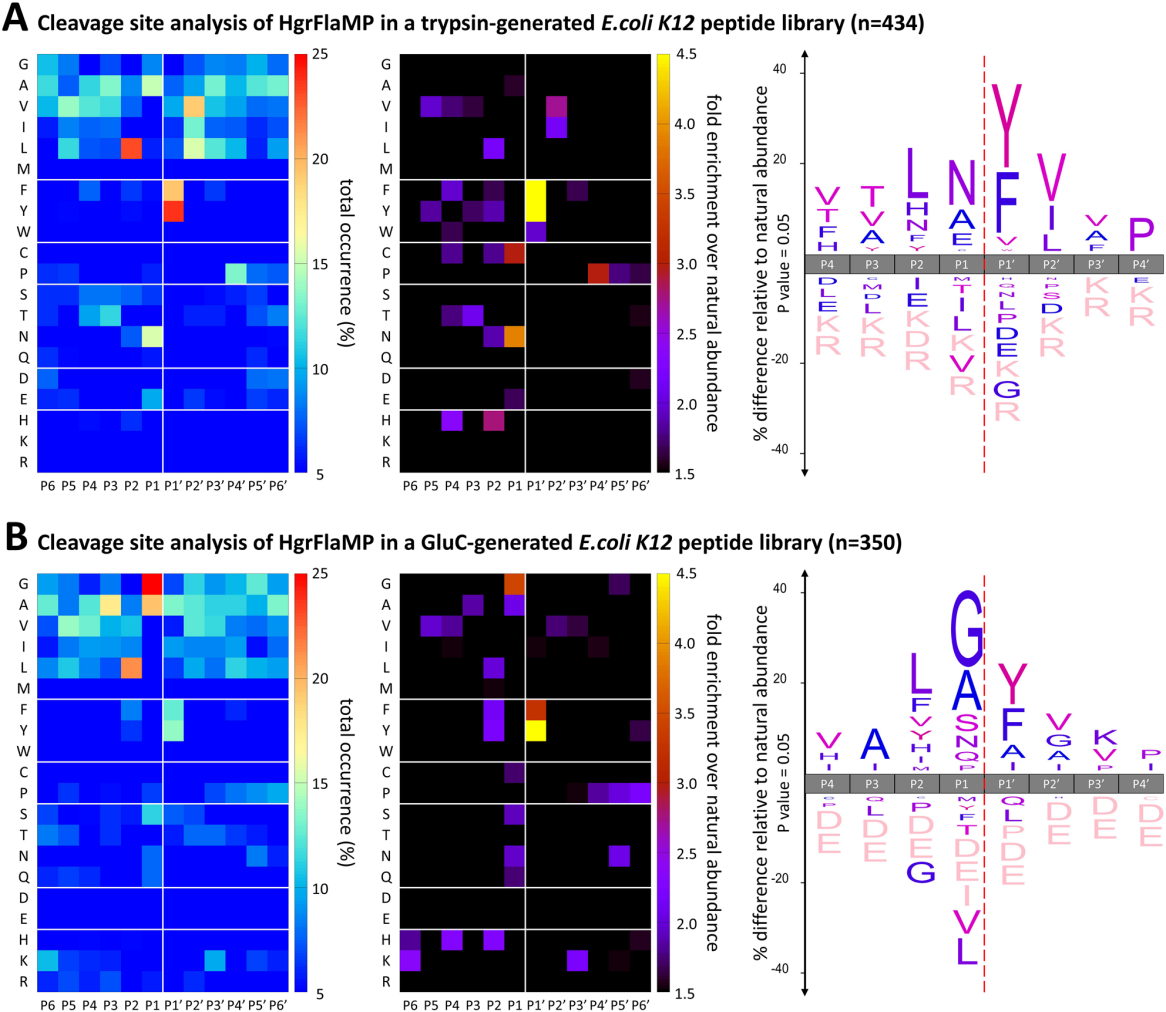
PICS specificity profiling of the proteolytic flagellin from *Hylemonella gracilis*. The alignment of 434 and 350 unique cleavage sites that were determined in a (**A**) trypsin- and (**B**) GluC-derived peptide library, respectively, is summarized as heat maps showing the relative occurrence (left) and fold-enrichment over natural abundance (middle) for the individual amino acids at the respective cleavage subsites. P6 to P6′ subsite positions are shown on the x-axis, and the single letter code for amino acid residues is plotted on the y-axis. In iceLogo^44^ (right), significantly over- and under-represented amino acids (p value 0.05) are shown above and below the x-axis, respectively, and amino acids that have not been identified are depicted in pink. Heat maps were created using gnuplot (www.gnuplot.info).

As a first step, we generated both trypsin- and GluC-generated peptide libraries from purified *E. coli* proteomes, which thereby provide complementary peptide substrates of biological amino acid diversity. The purified HgrFlaMP-HVR was then incubated with library aliquots in the presence of either EDTA (control) or calcium plus zinc, and cleavage products were identified together with non-cleaved library peptides by quantitative proteomics using light and heavy dimethylation for control and protease-treated samples, respectively. Following peptide-spectrum matching at a false discovery rate (FDR) of 1% using X!Tandem^42^ and PeptideShaker^43^, complete cleavage sites were bioinformatically inferred from the *E. coli* proteome (Supplemental Table 1), from which we obtained 434 and 350 unique P6 to P6′ cleavage sites for the trypsin- and GluC-generated peptide library, respectively (Fig. 5). Amino acid occurrences were counted for each sub-site, normalized to the natural amino acid abundance, and summarized as heat maps. Over- and under-representation of each amino acid was further analyzed using iceLogo^44^ to evaluate individual subsite preferences. Importantly, we used both trypsin- and GluC-generated peptide libraries to account for the amino acid bias introduced by the library enzyme; *i.e.* trypsin-generated peptide libraries lack internal basic residues while GluC libraries are devoid of acidic residues^45^. Consequently, a preference or dislike of acidic residues can only be seen in a tryptic library, as GluC-derived peptides have these amino acids only at their C-termini. Intriguingly, the two peptide libraries yielded similar, but not identical specificity profiles, but which is not unexpected due to the different peptide composition of the two libraries. While the substrate preferences at the P1′ and P2 positions—substrate and active-site cleft subsite nomenclature according to Schechter and Berger 1967^46^—were particularly conserved between the two PICS library preparations, a preference for glycine emerged only in the GluC-derived peptide library (Fig. 5B). Large hydrophobic and aromatic residues, such as phenylalanine and tyrosine, dominated P1′ in both libraries, and were also readily found in P2, where they were accompanied by other large amino acids such as leucine and histidine. Overall, these specificity results are largely similar to our specificity findings for *C. haemolyticum* flagellinolysin^1^. However, in *H. gracilis*, small (e.g. Ala, Gly) or medium-sized polar amino acids (e.g. Asn, Gln) dominated with the negatively charged glutamate in subsite P1, which contrasts the previously identified strong preference for both large hydrophobic and polar residues observed in *C. haemolyticum*. Moreover, the amino acid preference in the positions flanking the scissile bond, namely P1 and P1′, closely resembles the specificity profiles of mammalian matrix metalloproteinases (MMPs), where Gly/Ala and Asn/Glu are frequently found in P1 next to medium-to-large hydrophobic amino acids in P1′^45,47^. However, while MMPs additionally enrich for proline in P3, this feature is completely absent in both flagellinolysins, pointing to a comparably open S3 subsite, *i.e.* without the subsite roof typical for MMPs. This supports the aforementioned inability of *H. gracilis* to liquefy gelatin^33^ as gelatin contains around 24% of proline and hydroxyproline. Overall, the identified specificity profiles were rather broad, highlighting the cleavage promiscuity of flagellinolysins. Anchored by a mostly hydrophobic P2 residue and particularly by the P1′ position, which has a clear preference for an aromatic residue such as tyrosine or phenylalanine, the main determinants span only the central P2 to P2′ region, and only a few amino acids are significantly underrepresented relative to their natural abundance throughout the profile.

### Refinement of the peptide-derived specificity profile by using a set of proteinaceous substrates

To bolster the peptide-based specificity profiles obtained from the PICS experiments, we repeated the aforementioned substrate mix digests and analyzed them also by quantitative mass spectrometry. To do so, a protein substrate mix consisting of the bovine caseins alpha-1, alpha-2, beta, and kappa, and the structural and proteolytic flagellins from *Pseudoalteromonas tunicata* (PtuFlaMP), the latter as a mutationally inactivated HAxxH variant, was incubated with recombinant HgrFlaMP at a protease to substrate ratio of 1:25. To induce activity, 10 mM Ca^2+^ and 10 µM Zn^2+^ were added, while 10 mM EDTA was added to the negative control. After incubation for two hours at 22 °C, reactions were stopped by adding 100 mM EDTA, and the sample was halved. While one part was directly acidified and purified using C18 peptide clean-up tips, the other was incubated for another hour at 37 °C with trypsin to alter the physicochemical properties of HgrFlaMP-generated cleavage peptides that are otherwise inadequate for mass spectrometry analysis. The trypsin digests were stopped by addition of 0.1% formic acid, and the derived peptides purified as above. Without any further optimization of the cleavage conditions and by using HgrFlaMP alone, we obtained sequence coverages of 66%, 38%, 71%, and 45% for the mature bovine caseins alpha-S1, alpha-S2, beta, and kappa, respectively, (Supplementary Figures 3–6), and a total of 95 cleavage sites were identified in these four proteins. The corresponding peptide-spectrum matches are documented in Supplementary Table 2. Importantly, none of the identified peptides matched the respective signal peptides annotated in the UniProt database, validating our data-analysis pipeline as these are missing from mature caseins purified from bovine milk. Taking a closer look at alpha-S1-casein (Supplementary Figure 3), the uncovered part mainly corresponds to the amino acid stretch between Leu55 and Gly108. Importantly, this region has only one of the protein’s 18 tyrosine and phenylalanine residues, which are, however, the primary specificity determinants in P1′ based on our specificity analysis, and both Ser and Asp are enriched more than threefold, which are two rather disfavored residues by HgrFlaMP. Additionally, its only tyrosine residue is located in position 105, and cleavage would generate a tripeptide inaccessible for detection with our applied mass spectrometry settings.

**Figure 6:**
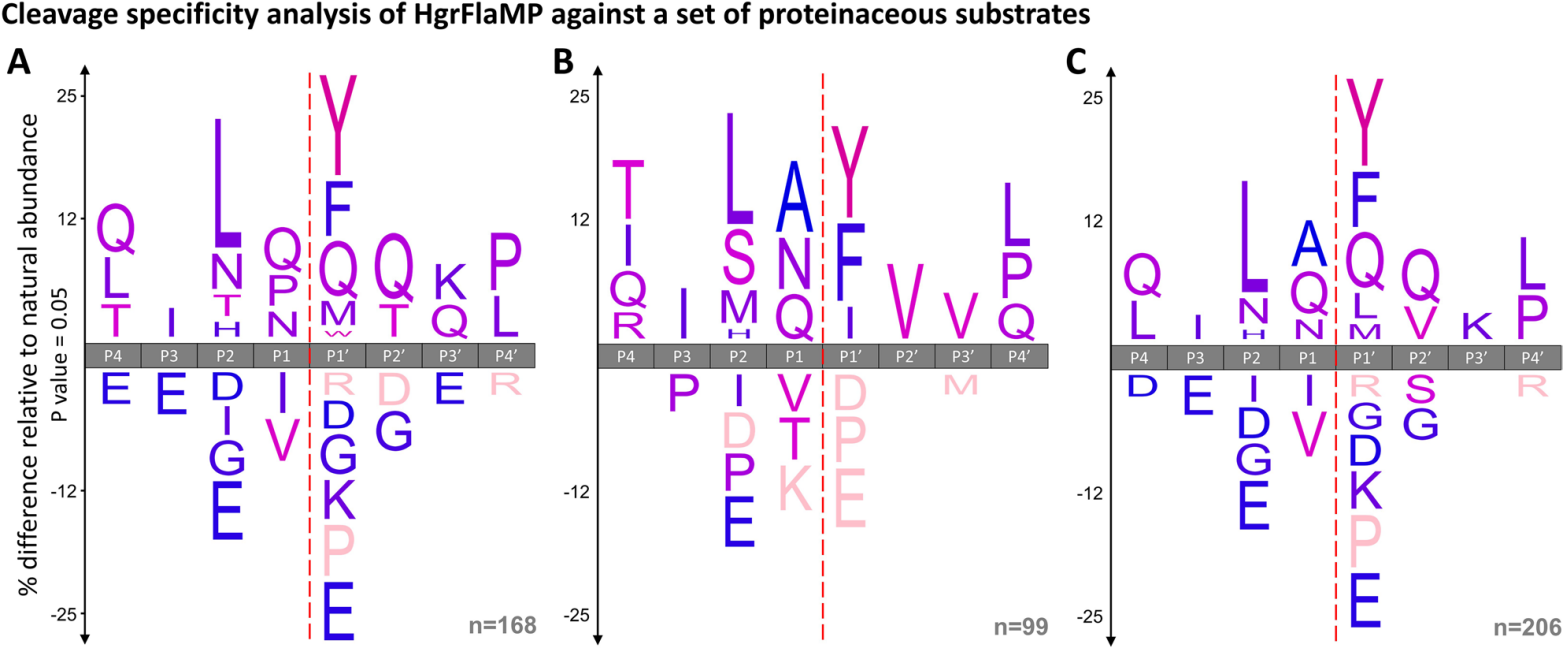
iceLogo representation of the proteolytic cleavage preference of HgrFlaMP using a set of six protein substrates. (**A**) HgrFlaMP specificity profile derived from 168 cleavage sites identified in bovine caseins and the structural and proteolytic flagellin from *P. tunicata*. (**B**) A total of 99 HgrFlaMP cleavage sites, 38 of which were intangible in the previous setting, were identified by a two-step digestion protocol utilizing trypsin as orthogonal cleavage enzyme. (**C**) Combined specificity logo based on 206 unique cleavage events, 95 within bovine caseins, and 111 in the flagellins from *P. tunicata*.

While we obtained a sequence coverage of 61% for the structural flagellin from *P. tunicata* (Supplementary Figure 9), we could only identify cleavage products accounting for 39% of its proteolytic counterpart despite similar molecular weight (Supplementary Figure 10). Intriguingly, the major difference is attributed to the central HVR region of the flagellinolysin, spanning approximately Leu155 to Gly593, which appears to be hardly cleaved by HgrFlaMP, while the N-terminal and C-terminal coiled-coil domains were readily degraded. And, contrary to the above-mentioned example of alpha-S1-casein, two thirds of the protein’s 32 tyrosine/phenylalanine residues reside within the HVR-region. Strikingly, the specificity logos obtained this way (Fig. 6) agreed well with our profiling results using PICS, especially with the one from the trypsin-derived peptide library (Fig. 5A). In total, we identified 206 unique cleavage sites using this approach, 168 after HgrFlaMP digest alone (Fig. 6A), 99 by the sequential digestion protocol, and hence 61 in both experimental set-ups (Fig. 6B). In P1′, tyrosine and phenylalanine were again the dominating residues, followed by glutamine, isoleucine, leucine and methionine. In P2, leucine was clearly preferred over asparagine and histidine, while in P1, glutamine, asparagine, and alanine were again favored. However, it is important to note that the repertoire of potential cleavage sites is less balanced in such a small protein-based cleavage assay than in a proteome-derived peptide library assay such as PICS. Finally, due to the sequence similarity between structural flagellins, especially in their N- and C-terminal domains (Supplementary Figure 11), we analogously expect potential cleavage of the *H. gracilis* structural flagellin by HgrFlaMP, and of other structural and proteolytic flagellins.

## Discussion

We successfully cloned, expressed, and purified HgrFlaMP, the putative flagellinolysin from *Hylemonella gracilis* (Figs. 1, 2, Supplementary Figure 1), and experimentally assessed its predicted function as an active protease capable of cleaving both peptide and protein substrates (Figs. 3, 4, 5, 6). Importantly, flagellinolysins were discovered only recently in 2017^1^, and so biochemical information about these enzymes is still scarce. Thus, this report represents the second characterization of a proteolytic flagellin, but the first from a Gram-negative organism.

Using proteomics and both proteome-derived peptide libraries and a set of intact protein substrates, we showed that HgrFlaMP is indeed a protease and that it exhibits a similar cleavage specificity to its previously characterized homolog from *C. haemolyticum*^1^. Substrate preference is dominated by large, predominantly hydrophobic residues in P1′, such as the aromatic amino acids phenylalanine and tyrosine, which are also favored in P2 together with leucine and histidine. Sequence specificity profiles obtained from the peptide-based PICS assay and the protein-cleavage assay are in good agreement, and perfectly explain the difference in sequence coverage for alpha-1 and alpha-2 casein by HgrFlaMP, which were 66 and 38% respectively. For beta-casein, we obtained a peptide coverage of over 71% in a single experiment without any optimization, and overall, we could identify more than 200 cleavage sites in a set of only six proteins, which pinpoints a rather broad sequence specificity combined with a high substrate promiscuity for proteolytic flagellins. Of note, the identified cleavage site preferences against both peptide libraries and a set of protein substrates must be interpreted collectively, and individual amino acid occurrences, fold enrichment, and relative difference to natural abundance have to be considered side-by-side for comprehensive interpretation, as each experiment and type of analysis highlights a slightly different aspect of the identified specificity profiles. Notably, the His6-SUMO-tagged protein exhibited lower stability than the tag-depleted enzyme after the addition of calcium and zinc, and the presence of substrate appears to stabilize the protease, probably by competing with self-cleavage. However, in the biological context of the bacterial flagellum, this may not be relevant as the N- and C-terminal coiled-coil domains form the inner backbone of the filament and are thus largely inaccessible for the HVR-domain, and the proteolytic units would be spread out over the filament with structural flagellins as additional spacer molecules.

While we did not succeed in purifying full-length HgrFlaMP because of its high propensity to self-aggregation during purification and autolysis, we did obtain crystallization-grade protein of our HVR variant. However, despite extensive trials, no crystallization conditions could be established at this point, but we are confident that our cleavage assays will help us to narrow down the minimal domain boundaries for a proteolytically active and stable variant. This may be of great importance, as with the current preparation we observe partial to complete self-cleavage, as indicated by an approx. 1–2 kDa shift in SDS-PAGE upon addition of calcium and zinc (Fig. 4). Intriguingly, when taking a closer look at our mass spectrometry cleavage data, we observed a preference for the N- and C-terminal coiled-coil regions in the proteolytic flagellin of *P. tunicata*, while the central HVR region remained nearly intact (Supplementary Figure 10). On the other hand, despite a slight preference for the terminal D0/D1 domains, complete degradation of the recombinant structural flagellin is observed in our degradomic experiment (Supplementary Figure 9), which supports the idea of flagellinolysins being able to target flagella from other organisms. Further work is needed to fully substantiate this finding.

Overall, flagellin-mediated proteolysis opens an entire avenue of potential biological functions. Flagellinolysins may act as secreted proteases, either after extracellular export via the flagellar type III secretion system, or by liberating the proteolytic HVR domain alone while leaving the filament intact. In doing so, proteolytic flagellins could perform any of the documented roles of secreted microbial proteases. However, if flagellinolysin activity is localized to the filament, this would also provide advantages by specific localization of proteolytic activity to external substrates that are in direct physical contact with the bacterium. Furthermore, retention of flagellinolysin within the flagellar filament avoids loss of enzymatic subunits through diffusion from the cell. The proteolytic activity of flagellinolysins could be of nutritional value, as intact proteins are more difficult to import than cleaved peptides that are generated in close proximity to the cell. Additionally, proteolytic flagellins may play a crucial role in host–pathogen interaction by degrading the extracellular matrix and thus aiding host infiltration and colonization. And in a biofilm setting, flagellinolysins may contribute to interspecies competition and microbial warfare by degrading the extracellular polymeric substance and/or proteinaceous cell wall compounds of competing bacteria, or aid biofilm formation and remodeling.

Notably, only monomeric but not filament-embedded flagellins activate Toll-like receptor 5 (TLR5), as the main TLR5 recognition motifs lie within the evolutionary conserved D0 and D1 domains^48–50^. Based on our proteomic data with recombinant protein, these same regions are preferentially targeted by flagellinolysins. Consequently, proteolytic flagellins could contribute to immune evasion by targeting flagellin monomers after their release from the filament, and thus deterring the binding of this prominent pathogen-associated molecular pattern to its cognate detector. Equivalently, proteolytic targeting of TLR5 and/or its associated adapter and signaling proteins may also abrogate a potential inflammatory and immune response, and thus may allow a pathogen to remain undetected during infection^51,52^.

In summary, we successfully produced a recombinant construct of the HVR-region of the flagellinolysin from *H. gracilis*, and performed a first biochemical characterization of its proteolytic activity using both classical cleavage assays and proteomics. We thereby established HgrFlaMP as the second characterized proteolytic flagellin to date and the first from a Gram-negative organism, and combined with the specificity profile of *C. haemolyticum* flagellinolysin, a common cleavage specificity profile is emerging for this protease family. Since flagellum-assisted proteolysis can be detected in hundreds of bacterial species across the tree of life throughout multiple environments, further characterization of flagellinolysin function and specificity will be important for understanding its potential roles in a wide range of possible activities, including biofilm development, interbacterial warfare, and host colonization and pathogenesis.

## Methods

### Bioinformatic and phylogenetic analysis

BLASTp searches were performed using the peptidase domain of the flagellinolysin from *C. haemolyticum* (NCBI accession number WP_039229452.1) to identify homologs in *Hylemonella gracilis* (UniProt ID F3KXG2), which were subsequently used to search the NCBI and UniprotKB databases for related proteins. A multiple sequence alignment of the top homologs was constructed using MUSCLE^53^ over the full-length proteins, and a phylogenetic analysis was performed using PhyML^54^ using the LG model and aLRT values for clade support, as implemented within SeaView^55^. Domain architectures were predicted using the NCBI’s Conserved Domain Database^36^ with default thresholds. Multiple sequence alignments of structural and proteolytic flagellins in the Supplementary Figures were performed using ClustalO^56^, and amino acid identity and similarity of protein regions calculated by BLASTp^57^. The molecular representation of the structural flagellin from *Salmonella typhimurium* (PDB entry 2uxu)^5^ was created in PyMol (The PyMOL Molecular Graphics System, Version 1.8 Schrödinger, LLC). Sequence Logo analysis of the active site HExxH motif and the putative calcium-binding site was performed using WebLogo^58^ with sequencing numbering according to the proteolytic flagellin FliA(H) from *Clostridium haemolyticum* (ChaFlaMP) to allow for better comparability with our previous sequence analysis of flagellinolysins^1^.

### Recombinant protein production

The full-length gene of the proteolytic flagellin from *Hylemonella gracilis* (UniProt ID F3KXG2) was synthesized de novo by GeneArt (Life technologies, Thermo Fisher). Both full-length HgrFlaMP (M1 to R565; approx. 59 kDa) and a variant representing the hypervariable region (HVR; L163-I450; approx. 30 kDa), were subcloned into pET28-N-His-SUMO, sequenced at Eurofins Genomics, and transfected into *E. coli* BL21 for protein expression. Cultures were inoculated 1:500 and grown at 37 °C and 230 rpm until an OD600 of ~ 1.2. Temperature was lowered to 20 °C, and cells induced with 1 mM isopropylthiogalactosid (IPTG). After 16 h induction, cells were harvested by centrifugation at 4000 RCF for 15 min at 10 °C, and cell pellets stored at − 20 °C until lysis. Cells were lysed by sonication in 10 ml of ice-cold Buffer A (100 mM Tris, 300 mM NaCl, 5 mM of beta-mercaptoethanol (bME), pH 8.0) per wet gram of pellet, and 10 µg/ml DNase (Thermo Fisher Scientific) and 10 mM MgSO_4_ were added for DNA removal. The mixture was incubated on ice for 1 h and soluble proteins were removed by centrifugation at 15,000 RCF and 4 °C. The pellet was resuspended using a Potter–Elvehjem Homogenizer in the original volume of chilled Buffer B (2 M Urea in 100 mM Tris, 300 mM NaCl, 5 mM bME, pH 8.0), incubated for 2 h on a rotator, and supernatant was removed by centrifugation. The remaining pellet containing insoluble proteins was resuspended in Buffer C (8 M Urea in 100 mM Tris, 5 mM bME, pH 8.0), incubated overnight, and clarified by two rounds of centrifugation prior loading the sample onto pre-equilibrated NiNTA gravity columns. After capture of the unfolded protein, a step-wise refolding protocol was performed from 6 to 0 M Urea at pH 8.0 in the presence of 100 mM Tris, 300 mM NaCl, 10 mM imidazole and 5 mM bME. For every refolding step, 10 column volumes of buffer were used. Prior elution of the target protein with 250 mM imidazole, columns were washed with 25 column volumes of 20 mM imidazole. Note, the last two buffers did not contain any bME. Elution samples were concentrated using centrifugal filtration (Amicon Ultra-15, MWCO 10 kDa, Merck), and aggregates removed via size-exclusion chromatography using a Superdex S200 16/60 GL column, and 50 mM Tris, 100 mM NaCl, pH 8.0, as running buffer. Peak fractions were pooled and incubated with home-made SUMO-protease overnight at a ratio of 1:1000 (w/w) in the presence of 5 mM bME. A second round of NiNTA ensured separation of the purification tag and the added SUMO protease from the target protein. Flow-through samples were concentrated and further purified using size-exclusion chromatography. Peak fractions were pooled and either used directly for biochemical analysis or frozen in liquid nitrogen as 50 µl aliquots at 1–5 mg/ml. All protein purification steps were performed at 4 °C in a cold room or on ice. Of note, protein preparations of HgrFlaMP appeared inactive after purification and needed addition of calcium and zinc to restore proteolytic activity.

### SDS PAGE and casein zymography

Proteins were separated using 10 or 12% SDS-PAGE under reducing or non-reducing conditions and stained using 1% Coomassie Brilliant Blue G250 (BioRad). The PageRuler Prestained Protein Ladder (Thermo Scientific) was used as molecular weight reference throughout the project. For zymography, protein samples were separated by SDS-PAGE containing 0.1% of bovine casein (Sigma Aldrich) under non reducing conditions. To remove SDS, gels were first washed with double distilled H2O, then rinsed twice with renaturation buffer (2.5% Triton X-100 in 50 mM Tris, 200 mM NaCl, 5 mM EDTA, pH 8.0), and incubated in the same buffer for 1 h. Next, gels were rinsed twice with developing buffer (0.02% Brij-35 in 50 mM Tris, 200 mM NaCl, 20 mM CaCl_2_, 20 µM ZnCl_2_, pH 8.0), and incubated for 2–4 h at room temperature, and stained using 0.5% Coomassie Brilliant Blue R250 (BioRad). Note, due to the lack of cysteine residues, the proteolytic flagellin from *H. gracilis* does not contain any disulfide bridges, and the protein behaves identical on reducing and non-reducing SDS-gels.

### In vitro cleavage assays of protein substrates

For protein-based cleavage assays, various proteins were incubated with recombinant flagellinolysin from *Hylemonella gracilis* at various protease to substrate ratios. The following proteins were included in the final substrate mix: bovine caseins (Sigma Aldrich), and the structural and proteolytic flagellins of *Pseudoalteromonas tunicata* (UniProt IDs A4C6G1 and A4C6E0, respectively). The latter was produced as a HAxxH variant, where the general base glutamate of the HExxH motif is exchanged to an alanine, rendering the enzyme inactive. The flagellin samples from *P. tunicata* were prepared as described above for *H. gracilis*. All cleavage assays were initiated by the addition of 10 mM CaCl_2_ and 10 µM ZnCl_2_, and negative controls were performed in the presence of 10 mM EDTA. Reactions were stopped after their respective time-points by adding 100 mM EDTA, and analyzed either by SDS-PAGE or mass spectrometry. For the proteomic analysis of the cleavage assay, the sample was split after inactivation of HgrFlaMP, and half was additionally incubated with trypsin to render otherwise too long peptides suitable for mass spectrometry. Reactions were stopped after 1 h at 37 °C by adding formic acid to a final concentration of 0.1%. All samples were purified via C18 tips (100 µl bed, Pierce, Thermo Fisher Scientific, Vienna, Austria) prior HPLC–MS/MS analysis.

### Protease specificity profiling using proteome-derived peptide libraries

Proteomic identification of protease cleavage sites (PICS) experiments were performed with trypsin and GluC-derived peptide libraries from whole proteome extracts of *Escherichia coli* as described in great detail in the literature^45,59,60^. PICS cleavage assays were performed by incubation of 50 to 100 µg of peptide library with recombinant flagellinolysin at a protease to peptide library ratio of 1:50 (w/w) in 50 mM HEPES, 150 mM NaCl, 10 mM CaCl_2_, 10 µM ZnCl_2_, pH 7.4. For negative controls, 10 mM EDTA was added instead of zinc and calcium ions, and reactions were stopped by the addition of 100 mM EDTA after overnight incubation at 20 °C. Peptides were stable isotope labeled by reductive dimethylation with CH_2_O and ^13^CD_2_O for controls and protease‐treated samples, respectively, cysteines were carbamidomethylated, and reactions stopped by addition of 100 mM Tris‐HCl (pH 7.5). Protease‐treated samples and controls were subsequently mixed and purified via C18 tips.

### High-performance liquid chromatography and mass spectrometry

Peptide separations were carried out on a nanoHPLC instrument (UltiMate U3000 RSLCnano, Thermo Fisher Scientific, Germering, Germany) equipped with an Acclaim PepMap 100 C18 column (500 × 0.075 mm i.d., 3 µm particle size, 100 Å pore size, Thermo Fisher Scientific, Sunnyvale, CA, USA) at a flow rate of 350 nL^.^min^-1^ and a column oven temperature of 50 °C. Eluent A (H_2_O (MilliQ Integral 3 instrument, Millipore, Billerica, MA, USA) + 0.1% formic acid (Sigma-Aldrich)) and eluent B (acetonitrile (VWR International, Vienna, Austria) + 0.1% formic acid) were employed in a stepped linear gradient as follows: 1.0–22% B in 200 min, 22–40% in 40 min, 80% B for 20 min and 1.0% B for 40 min. Samples were injected in full-loop mode (loop volume 1.0 µl) at a peptide concentration of 2.0 mg mL^−1^. Mass spectrometric data was acquired on a quadrupole-Orbitrap hybrid mass spectrometer (Thermo Scientific QExactive Plus benchtop quadrupole-Orbitrap mass spectrometer) hyphenated to the HPLC system via a Nanospray Flex ion source (Thermo Fisher Scientific, Bremen, Germany). A SilicaTip emitter with 360 µm o.d., 20 µm i.d. and a tip i.d. of 10 µm (New Objective, Woburn, MA, USA) was used for electrospray ionization (ESI). Spray voltage was set to 1.5 kV, S-lens RF level to 55.0, and capillary temperature to 320 °C. A MS1 AGC target of 3e6 was employed in an *m/z* range of 400–2000 with a maximum injection time of 100 ms. Each MS1 scans at a resolution setting of 70,000 at 200 m*/z* was followed by 15 data-dependent MS2 scans for PICS experiments and 10 data-dependent MS2 scans for cleavage experiments on protein substrates, both at a resolution of 17,500 at *m/z* 200. HCD fragmentation was carried out at 28.0 NCE in a 2.0 m*/z* isolation window with an AGC target of 1e5 and a maximum injection time of 100 ms. Fragmented peptides were excluded from fragmentation for 30 s. The mass spectrometer was calibrated using the Pierce LTQ Velos ESI Positive Ion Calibration Solution from Life Technologies (Vienna, Austria), and the MS raw data associated with the present paper are available upon request.

### Proteomic data analysis

Thermo raw files were converted to .mgf peaklists using ProteoWizard msConvert (v3.0.20075)^61,62^, and data were analyzed using X!Tandem^42^ within SearchGUI (v3.3.19)^63^ for peptide-spectrum matching and PeptideShaker (v1.16.45) for statistical analysis^43^. PICS cleavage data were searched non-specific against a decoy database containing the UniProt annotated *E. coli* K12 proteome (downloaded December 2019)^64^, common contaminants such as human keratins, and proteases under investigation. For the substrate-based cleavage assay, the amino acid sequences of bovine caseins alpha-1, alpha-2, beta, and kappa were added to the database together with the flagellins from *P. tunicata*. Search parameters included a mass tolerance of 20 ppm for both parent and fragment ions, and methionine oxidation (+ 15.99 Da) and cysteine carboxyamidomethylation (+ 57.02 Da) were set as variable. Search settings allowed for free and dimethylated lysines and N-termini (light dm: + 28.05 Da; heavy dm: + 34.06 Da), and the “quick-pyrrolidone” (− 17.03 Da from Q; − 18.02 Da from E) and “quick-acetyl” (+ 42.01 Da) options were enabled. Results were imported into PeptideShaker, analyzed to a false discovery rate of 1%, and exported as csv-files for further analysis. The complete cleavage sites were reconstructed similar to the web-based program WebPICS^65^, and the generated non-redundant cleavage sites summarized as heat maps using the open source plotting tool Gnuplot (www.gnuplot.info) and iceLogos^44^. Heat maps show either relative occurrence or fold-change over natural abundance of the 20 proteinaceous amino acids at the P6 to P6′ cleavage position, while iceLogos^44^ were used to display the percent difference of occurrence compared to natural abundance. Of note, for both the heat maps and iceLogo analysis, the *E. coli K12* reference proteome was used to ensure comparability with our previous specificity profiling data on clostridial collagenases^66^, the human matrix metalloproteinase family^45,67^, and the proteolytic flagellin FliA(H) from *Clostridium haemolyticum*^1^. Please see Supplementary Tables 1 and 2 for all identified peptide-spectrum matches and cleavage sites. For the protein coverage maps shown in Supplementary Figures 3 to 6 and 9 to 10, the MS Tools web application *Draw Map* (https://www.hxms.com/mstools/) was used^68^. Importantly, peptide-based specificity profiling is not designed for the discovery of folded substrates; thus, the provided UniProt identifiers are for reference only.

## Supplementary information

Supplementary Figures.

Supplementary Table 1.

Supplementary Table 2.
