# Supplementary material for: Identification and characterization of the proteolytic flagellin from the common freshwater bacterium Hylemonella gracilis
Source: Sci Rep. 2020 Nov 4;10:19052. doi: 10.1038/s41598-020-76010-8 (PMC7643111; doi:10.1038/s41598-020-76010-8)

# Identification and characterization of the proteolytic flagellin from the common freshwater bacterium *Hylemonella gracilis*

Ulrich Eckhard[1,2,*], Constantin Blöchl[1], Benjamin G. L. Jenkins[3], Michael J. Mansfield[3,§], Christian G. Huber[1,4], Andrew C. Doxey[3,#,*], and Hans Brandstetter[1,4,#].

1. Department of Biosciences, University of Salzburg, Hellbrunner Straße 34, 5020 Salzburg, Austria.
2. Proteolysis Lab, Department of Structural Biology, Molecular Biology Institute of Barcelona, CSIC, Barcelona Science Park, Baldiri Reixac, 15-21, 08028 Barcelona, Catalonia, Spain.
3. Department of Biology, University of Waterloo, 200 University Ave. West, Waterloo, Ontario N2L 3G1 Canada.
4. Christian Doppler Laboratory for Innovative Tools for Biosimilar Characterization, University of Salzburg, Hellbrunner Straße 34, 5020 Salzburg, Austria.

§Present address: Genomics and Regulatory Sytems Unit, Okinawa Institute of Science and Technology Graduate University, Onna, Okinawa, 904-0495, Japan.

#These authors share senior authorship.

***Correspondence:** Ulrich Eckhard (ueccri@ibmb.csic.es) or Andrew C. Doxey (acdoxey@uwaterloo.ca).

# Supplementary Figures

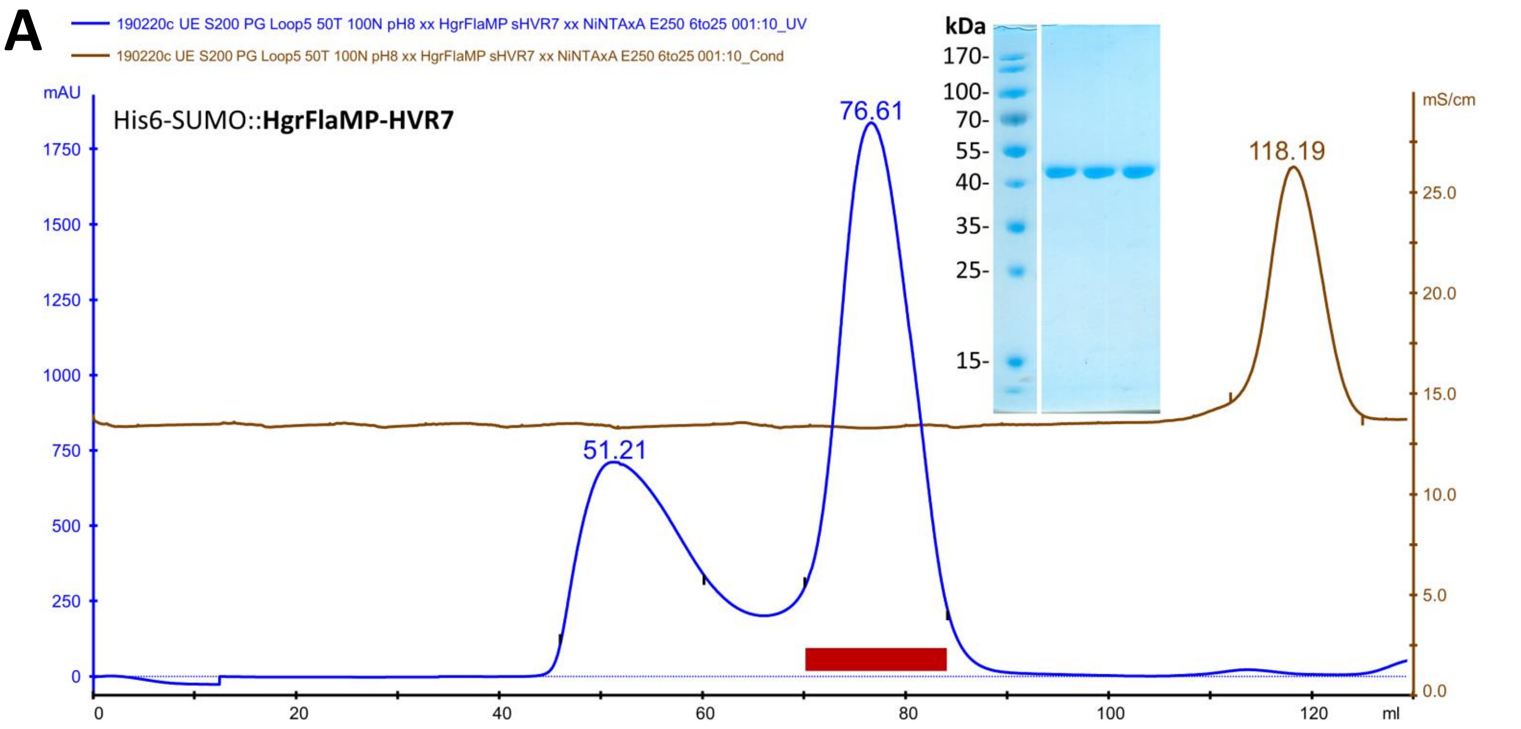


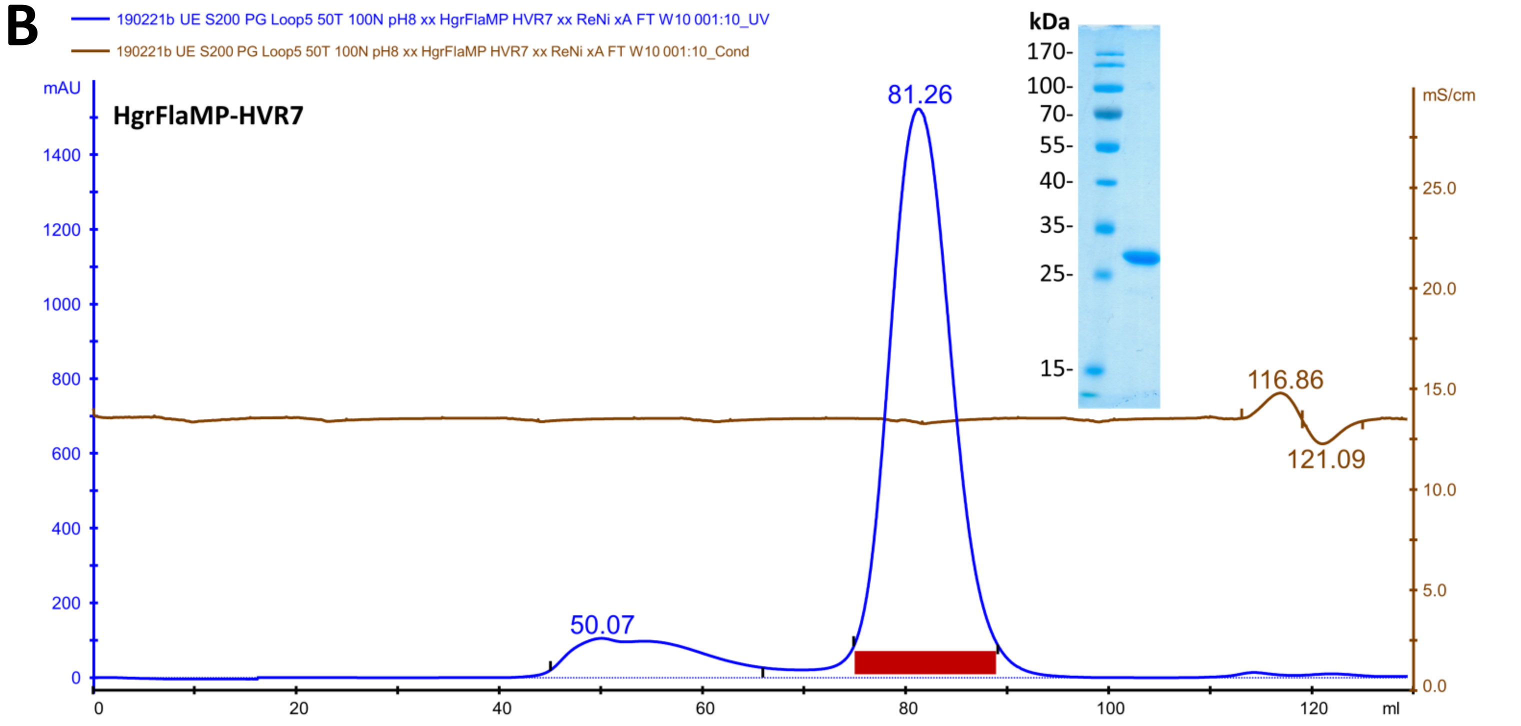


**Supplementary Figure 1.** Size exclusion chromatography of recombinant flagellinolysin HVR-protein from *Hylemonella gracilis*. **(A)** His-SUMO-tagged HgrFlaMP-HVR after NiNTA purification. Injection volume: 5ml. Running buffer: 50 mM Tris, 100 mM NaCl, pH 8.0. Target protein eluted as monodisperse peak at approx. 76.6 ml. SDS-PAGE insert shows three representative peak samples prior pooling. **(B)** Tagless HgrFlaMP-HVR protein after NiNTA SUMO-protease digest and rechromatography. Loop volume: 5ml. Injection buffer: 50 mM Tris, 100 mM NaCl, pH 8.0. Target protein eluted as monodisperse peak at approx. 81.3 ml. SDS-PAGE insert shows the peak sample after pooling.

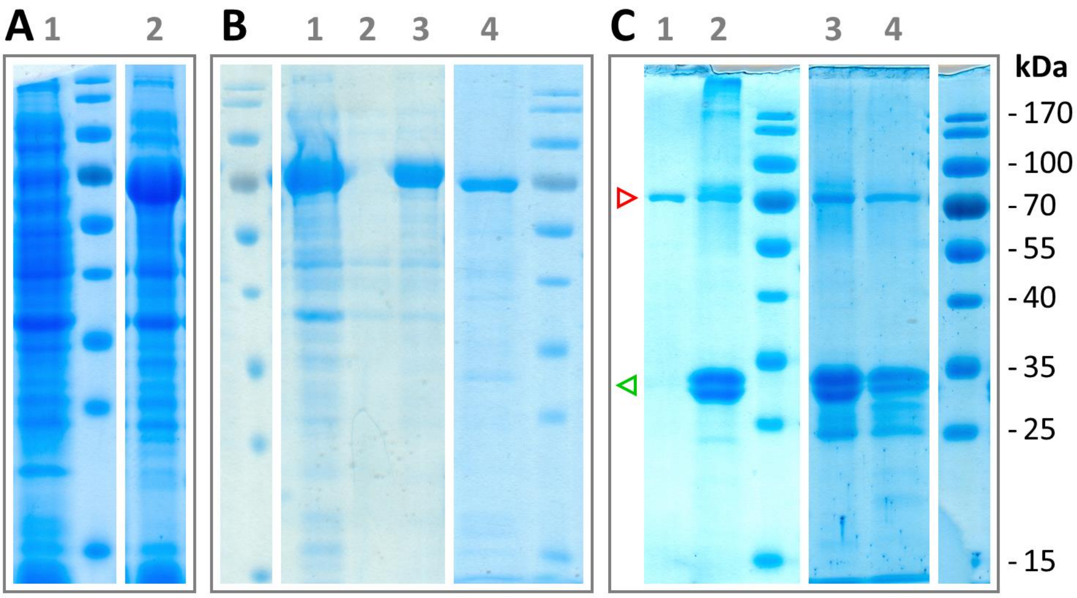


**Supplementary Figure 2.** Expression and purification of His6-SUMO tagged full-length flagellinolysin from *Hylemonella gracilis*, and proteolytic cleavage of bovine casein. **(A)** Expression samples before and after induction with IPTG. **(B)** Cell lysis and protein purification using NiNTA chromatography. 1 total lysate. 2 soluble protein after lysis. 3 insoluble protein fraction. 4 recombinant protein after NiNTA purification. **(C)** Proteolytic activity against bovine casein. 1 recombinant HgrFlaMP-FL (red triangle). 2 incubation with bovine casein (green triangle) prior addition of calcium and zinc. 3 sample after two hours of incubation in presence of 10 mM EDTA. 4 sample after two hours of incubation in presence of 10 mM CaCl2 and 10 µM ZnCl2.

**Supplementary Figure 3** **Eckhard et al. 2020**

## A Sequence coverage of alpha-S1-casein from *Bos taurus* by HgrFlaMP.

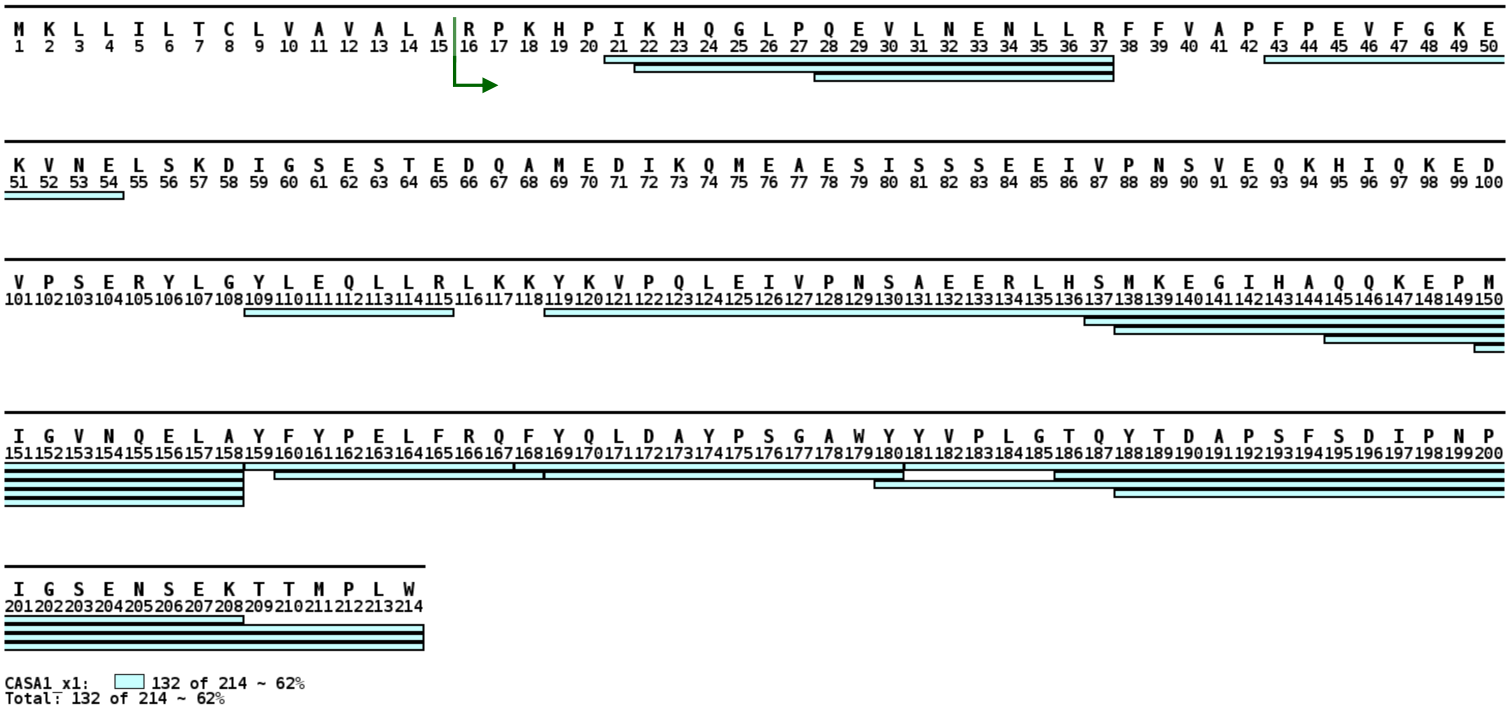


## B Identified cleavage sites (P1-P1') in alpha-S1-casein from *Bos taurus* by HgrFlaMP.

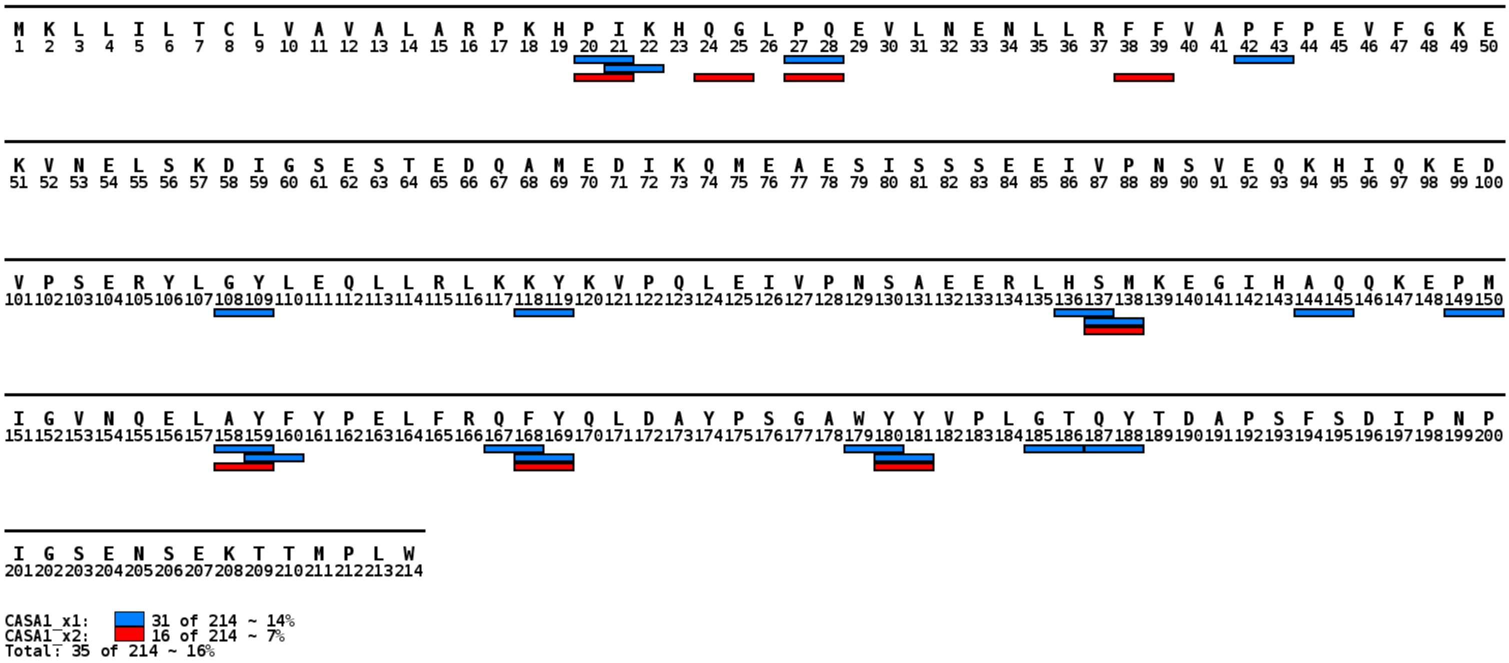


**Supplementary Figure 3. (A)** Sequence coverage of alpha-S1-casein from *Bos taurus* after digestion with recombinant flagellinolysin from *Hylemonella gracilis* (HgrFlaMP-HVR). A protein coverage of 61.7% was obtained for the full-length protein. The green arrow indicates the start of the mature protein. **(B)** HgrFlaMP cleavage sites are indicated by colored bars spanning P1 to P1'. A total of 20 non-redundant cleavage sites could be identified. Blue bars: 18 cleavage sites derived from the proteomic experiment shown above. Red bars: 8 HgrFlaMP-derived cleavage sites after additional trypsin-digestion to render otherwise too long peptides suitable for mass spectrometry. LC-MS/MS data were collected using a Q-Exactive Plus mass spectrometer (Thermo Scientific), peptide-spectrum matches identified with X!Tandem (Craig and Beavis 2004), and validated with PeptideShaker (Vaudel et al. 2015) at a false discovery rate of 1%. Figures were created using the web-tool Draw Map within the MS Tools collection (Kavan and Man 2011).

**A** Sequence coverage of alpha-S2-casein from *Bos taurus* by HgrFlaMP.

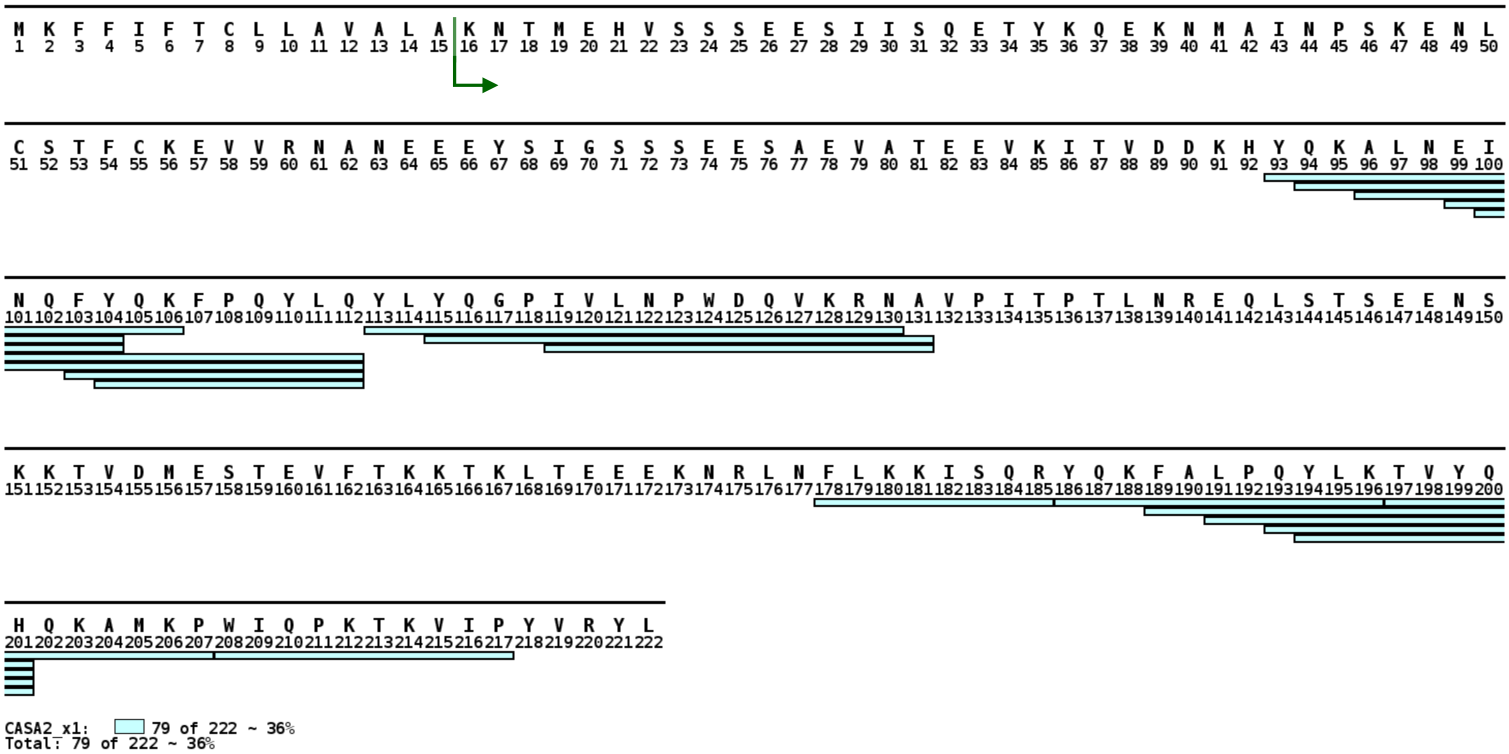


**B** Identified cleavage sites (P1-P1’) in alpha-S2-casein from *Bos taurus* by HgrFlaMP.

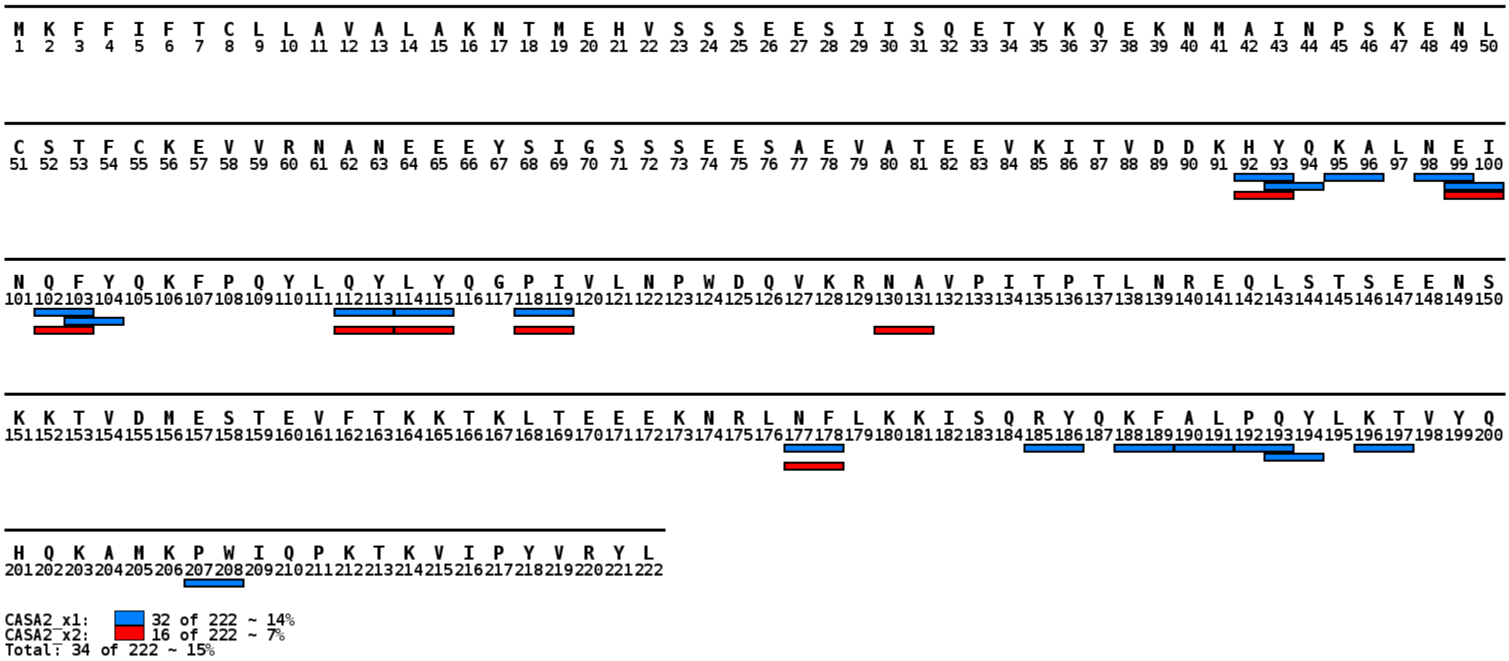


**Supplementary Figure 4. (A)** Sequence coverage of alpha-S2-casein from *Bos taurus* after digestion with recombinant flagellinolysin from *Hylemonella gracilis* (HgrFlaMP-HVR). A protein coverage of 35.6% was obtained for the full-length protein. The green arrow indicates the start of the mature protein. **(B)** HgrFlaMP cleavage sites are indicated by colored bars spanning P1 to P1’. A total of 19 non-redundant cleavage sites could be identified. Blue bars: 18 cleavage sites derived from the proteomic experiment shown above. Red bars: 8 HgrFlaMP-derived cleavage sites after additional trypsin-digestion to render otherwise too long peptides suitable for mass spectrometry. LC-MS/MS data were collected using a Q-Exactive Plus mass spectrometer (Thermo Scientific), peptide-spectrum matches identified with X!Tandem (Craig and Beavis 2004), and validated with PeptideShaker (Vaudel et al. 2015) at a false discovery rate of 1%. Figures were created using the web-tool Draw Map within the MS Tools collection (Kavan and Man 2011).

## A Sequence coverage of beta-casein from *Bos taurus* by HgrFlaMP.

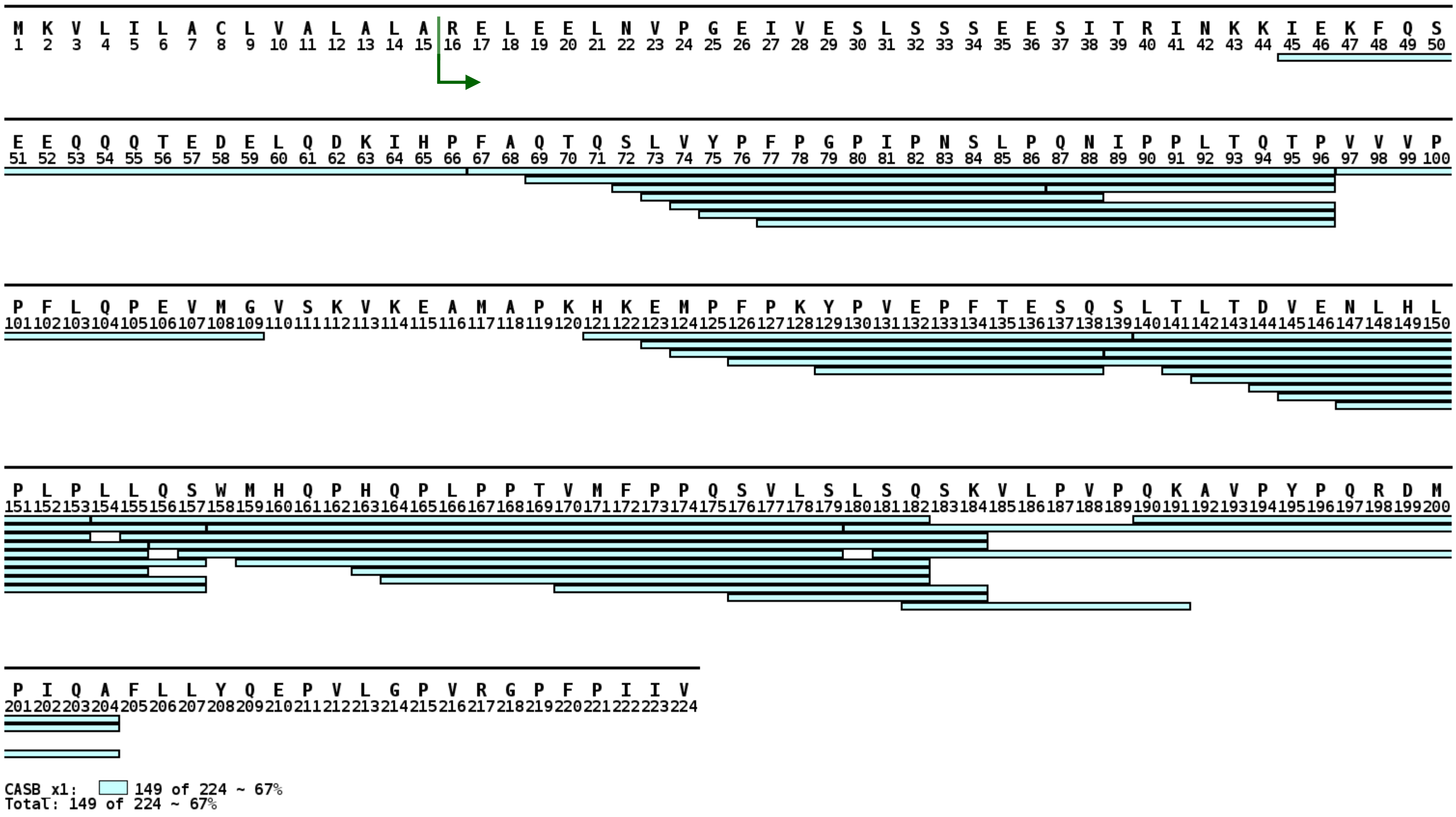


## B Identified cleavage sites (P1-P1') in beta-casein from *Bos taurus* by HgrFlaMP.

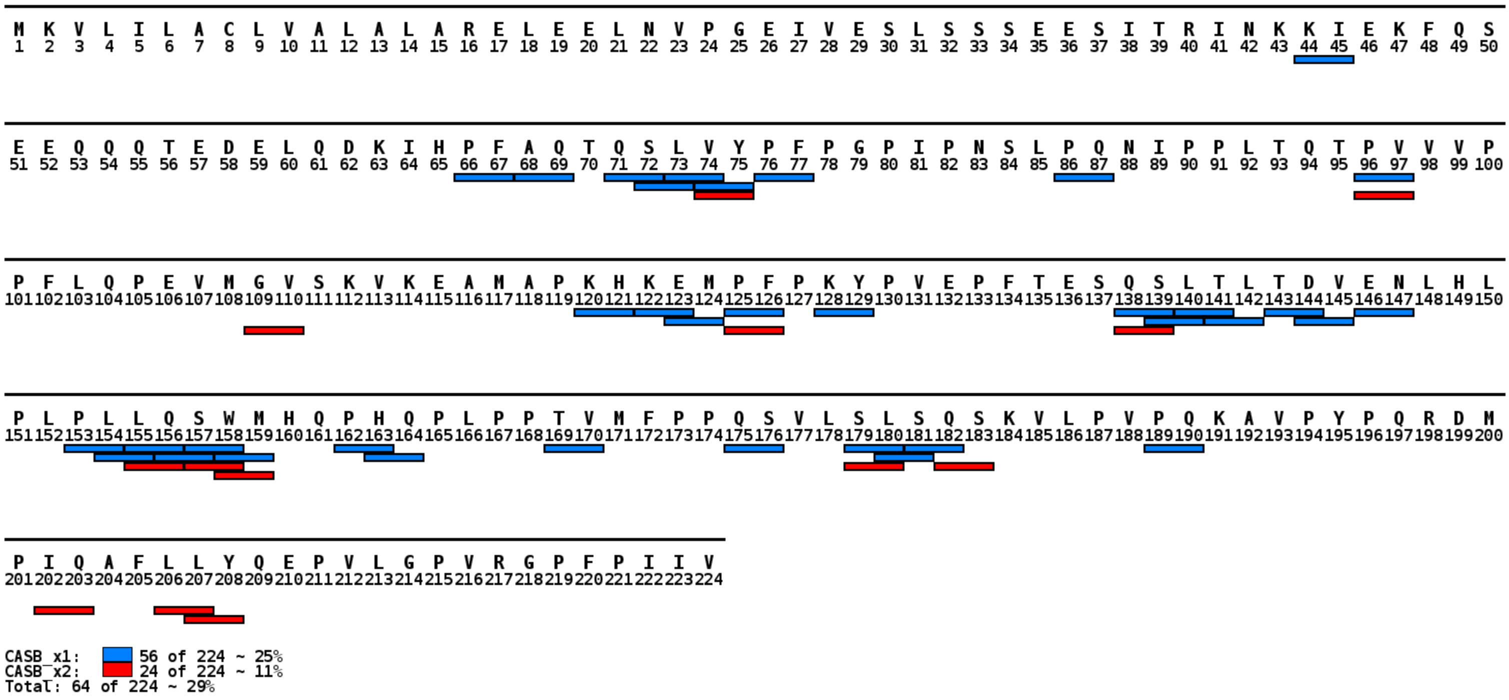


**Supplementary Figure 5. (A)** Sequence coverage of beta-casein from *Bos taurus* after digestion with recombinant flagellinolysin from *Hylemonella gracilis* (HgrFlaMP-HVR). A protein coverage of 66.5% was obtained for the full-length protein. The green arrow indicates the start of the mature protein. **(B)** HgrFlaMP cleavage sites are indicated by colored bars spanning P1 to P1'. A total of 41 non-redundant cleavage sites could be identified. Blue bars: 36 cleavage sites derived from the proteomic experiment shown above. Red bars: 13 HgrFlaMP-derived cleavage sites after additional trypsin-digestion to render otherwise too long peptides suitable for mass spectrometry. Figures were created using the web-tool Draw Map within the MS Tools collection (Kavan and Man 2011).

**A** Sequence coverage of kappa-casein from *Bos taurus* by HgrFlaMP.

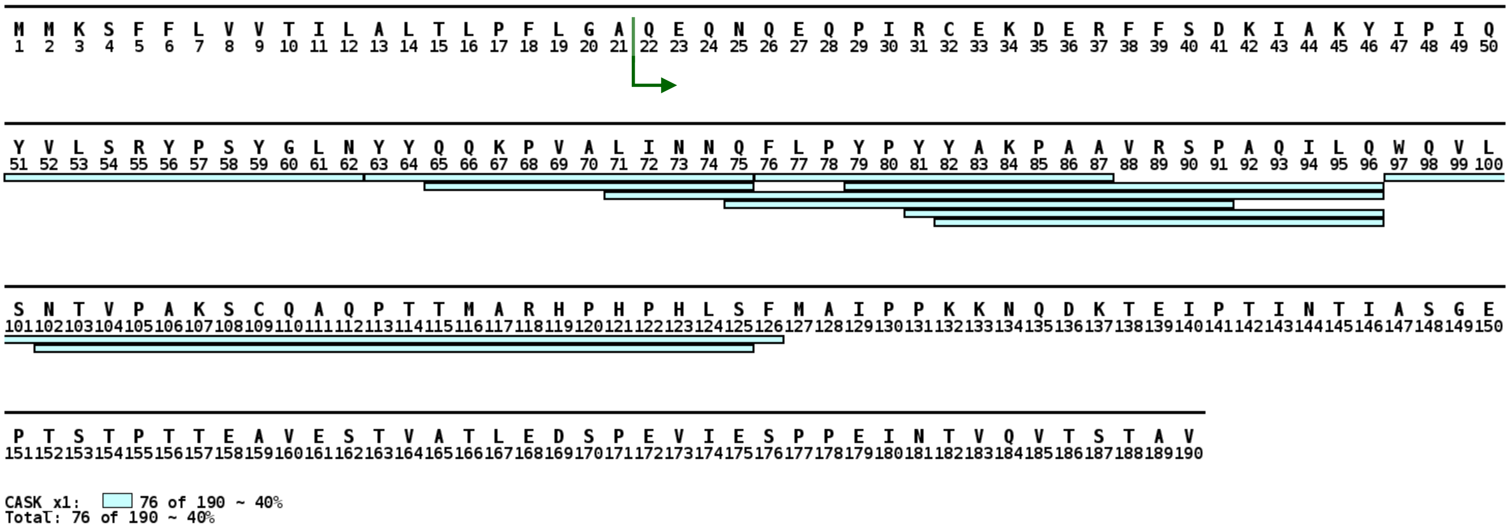


**B** Identified cleavage sites (P1-P1’) in kappa-casein from *Bos taurus* by HgrFlaMP.

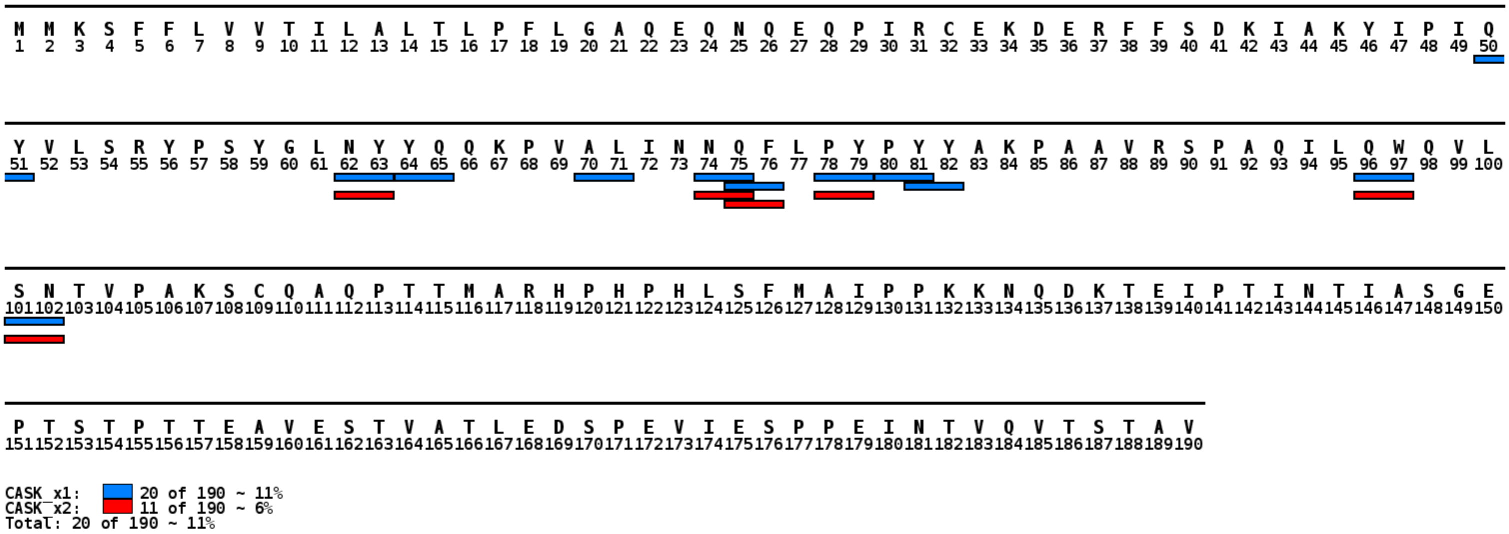


**Supplementary Figure 6. (A)** Sequence coverage of kappa-casein from *Bos taurus* after digestion with recombinant flagellinolysin from *Hylemonella gracilis* (HgrFlaMP-HVR). A protein coverage of 40.0% was obtained for the full-length protein. The green arrow indicates the start of the mature protein. **(B)** HgrFlaMP cleavage sites are indicated by colored bars spanning P1 to P1’. A total of 11 non-redundant cleavage sites could be identified. Blue bars: 11 cleavage sites derived from the proteomic experiment shown above. Red bars: 6 HgrFlaMP-derived cleavage sites after additional trypsin-digestion to render otherwise too long peptides suitable for mass spectrometry. LC-MS/MS data were collected using a Q-Exactive Plus mass spectrometer (Thermo Scientific), peptide-spectrum matches identified with X!Tandem (Craig and Beavis 2004), and validated with PeptideShaker (Vaudel et al. 2015) at a false discovery rate of 1%. Figures were created using the web-tool Draw Map within the MS Tools collection (Kavan and Man 2011).

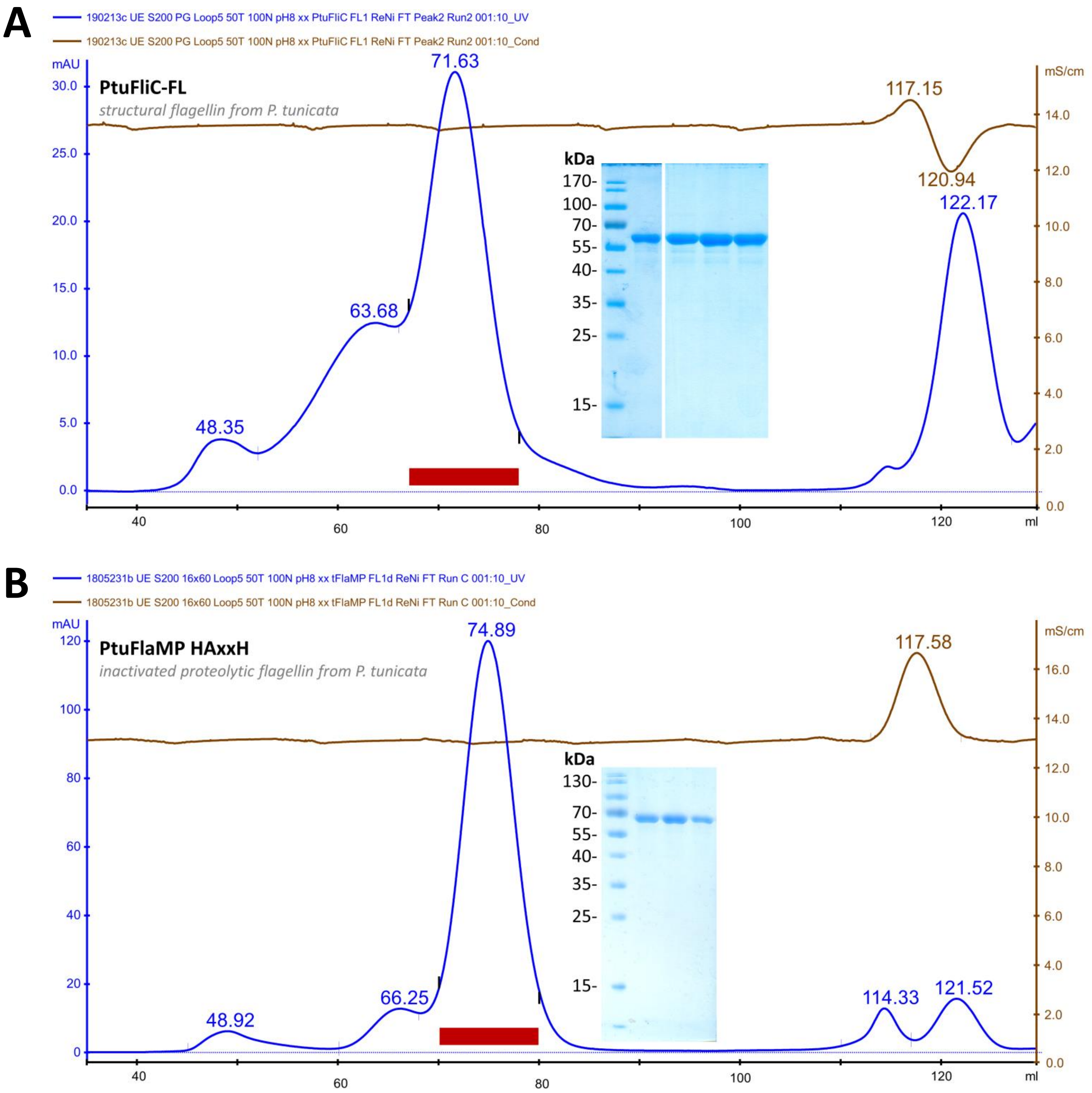


**Supplementary Figure 7. (A)** Size exclusion chromatography of recombinant full-length structural flagellin from *Pseudoalteromonas tunicata* (PtuFliC). **(B)** Size exclusion chromatography of the HAxxH-inactivated full-length proteolytic flagellin from *Pseudoalteromonas tunicata* (PtuFlaMP HAxxH). After His6-SUMO-tag removal and rechromatography, the two flagellin proteins eluted as monodisperse peaks at 71.6 (**A**) and 74.9ml (**B**), respectively. Injection volume: 5 ml. Running buffer: 50 mM Tris, 100 mM NaCl, pH 8.0. SDS-PAGE inserts shows the representative protein samples from the peak fractions.

## A Multiple sequence alignment of ten selected flagellinolysins around the active site.

```
HgrFlaMP    AGGTAARVTSLA-----GPS--GPGTNIT LEI D MADF VPPNLPNGG--TAPFYSDRII 247
ChaFlaMP    AYGVLASVTP------------KTGEFE LHI D SSDF EKGDGESGNNIHGKLYDDRII 231
PtuFlaMP    DGGTAAYVKP-A-------YSGGQAVQMV MVI D KGDF ATSADIHDD---SSLALNETL 235
AkaFlaMP    AGGVAAYVQV-T-----GFDGQGRGNNLV LQV D MADF VPPNPPNGG--SAPFYNDRVI 245
BafiFlaMP   EGGTLAYVSSLV-----PSATGGTGTNIS MTI D MADF LPASWPNGG--GSFIYNDRIV 241
BufiFlaMP   GGSTLAYASLASAIGNDGTSYSVHGITLG MTV D KDDF SNSSVD-GSTGNGTNKLESTI 298
CboFlaMP    AYGTLAYVGG------------KTGELE LHI D SSDF EKGDGESGNNIHGKLYDDRII 231
DmeFlaMP    GNDFVAWVSN-A-----YNTTTKEAVSQE LHI D LSDF TPVTTPDGG--TAPYYNDRII 242
NmaFlaMP    AGGVAAQVVGMI-----PGSFTGKATDVK LQI D MSDF TPPNLPNGG--TAPIYNDRII 244
TacFlaMP    ASGVLASVS----------YLAPAGTELM MNI D LDDF ST---LDGD---KTNQLKSTL 235
                  .  *  .                 : :  *   **        ..      .  :

HgrFlaMP    A HEMVH AIMATGQSWGELVN-D-GGATWFL E GVAEFIHGADERVQADGVPATL----- 298
ChaFlaMP    Q HEMTH AVMNDALGIDKMNDLHDKNKLWFI E GTAEAMAGADERVKDIIGNDTQTG-ID 287
PtuFlaMP    L HEMVH ATMASNFQL-------TNLPIWFL E GTAEAIRGADSRVAQDSAGLGLKNIID 286
AkaFlaMP    A HEMVH AVMARSTNWQNIT----GSHLWFA E GAAEFIHGAEERVRADVANLGVAA--- 296
BafiFlaMP   A HEMVH AVMAATLNWGHTSADNPAIPKWFK E GTAEFLAGGDERLKSSLTTESASD--- 296
BufiFlaMP   A HELMH TIMQYTMPVAMAKD---FFPTWFT E GVAQLTGGGVATNWNNGLYNAVSGITS 353
CboFlaMP    Q HEMTH AVMNDALGLDKMNDLHKNNKVWFV E GTAEAMAGADERVKGVIGNNTQTAI-- 287
DmeFlaMP    A HEMVH AVMGVTMNS-------YGMSTWFQ E GAAELIQGADERLSGAINARLGGGETI 293
NmaFlaMP    A HEMVH AVMYRSMNIASMFD-PAVDQEWFL E GTAEFIHGADERLQSSINSIGIGG--- 298
TacFlaMP    L HEMTH AVMANQMDL-------ASTPTWFR E GTAEAVSGADDRVASDIANFGVAALKA 286
              **: *  : *                **   * *.*:    *.
```

## B Sequence logos of the putative $Ca^{2+}$ binding site (top) and the active site HExxH motif (bottom).

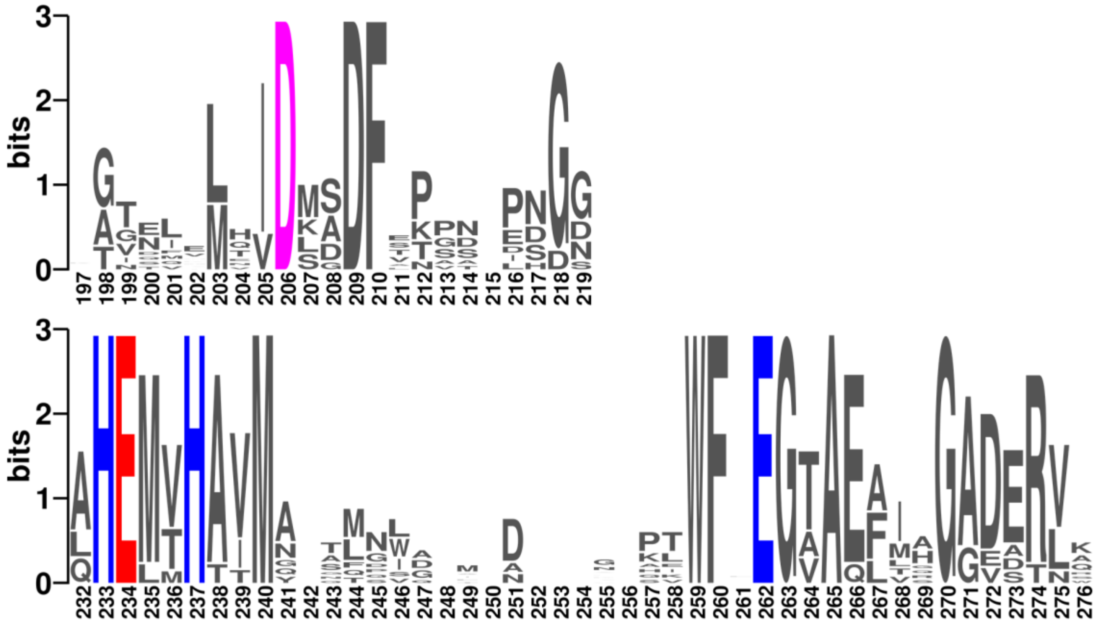

**Supplementary Figure 8. (A)** Multiple sequence alignment of the active site region of 10 selected flagellinolysins using Clustal Omega (Madeira et al. 2019). The proteolytic flagellins from the following species were used *Hylemonella gracilis* (HgrFlaMP; WP_006299301), *Clostridium haemolyticum* (ChaFlaMP; BAB87738), *Pseudoalteromonas tunicata* (PtuFlaMP; EAR29544), *Acidovorax kalamii* (AkaFlaMP; WP_094287282), *Bacillus firmus* (BafiFlaMP; WP_035329181), *Butyrivibrio fibrisolvens* (BuFiFlaMP; WP_073390425), *Clostridium botulinum* (CboFlaMP; WP_039258875), *Desulfobulbus mediterraneus* (DmeFlaMP; WP_035245515), *Nitrosomonas marina* (NmaFlaMP; WP_090628462), and *Thalassomonas actiniarum* (TacFlaMP; WP_044831744). **(B)** Sequence Logo analysis of the and the active site HExxH motif and the putative calcium-binding site. Sequence logos were generated using WebLogo (Crooks et al. 2004). Sequencing numbering is based on the flagellinolysin FliA(H) from *Clostridium haemolyticum* (ChaFlaMP) to allow for better comparability with our previous analysis (Eckhard et al. 2017). The zinc binding residues are highlighted in blue, the general base glutamate in red, and the putative calcium-binding aspartate in magenta. Of note, in the structural homolog, the clostridial collagenases, the calcium-binding site is built-up by one acidic residue (glutamate), and three backbone oxygens (Eckhard et al. 2013).

**A** Sequence coverage of the structural flagellin FliC from *Pseudoalteromonas tunicata* by HgrFlaMP.

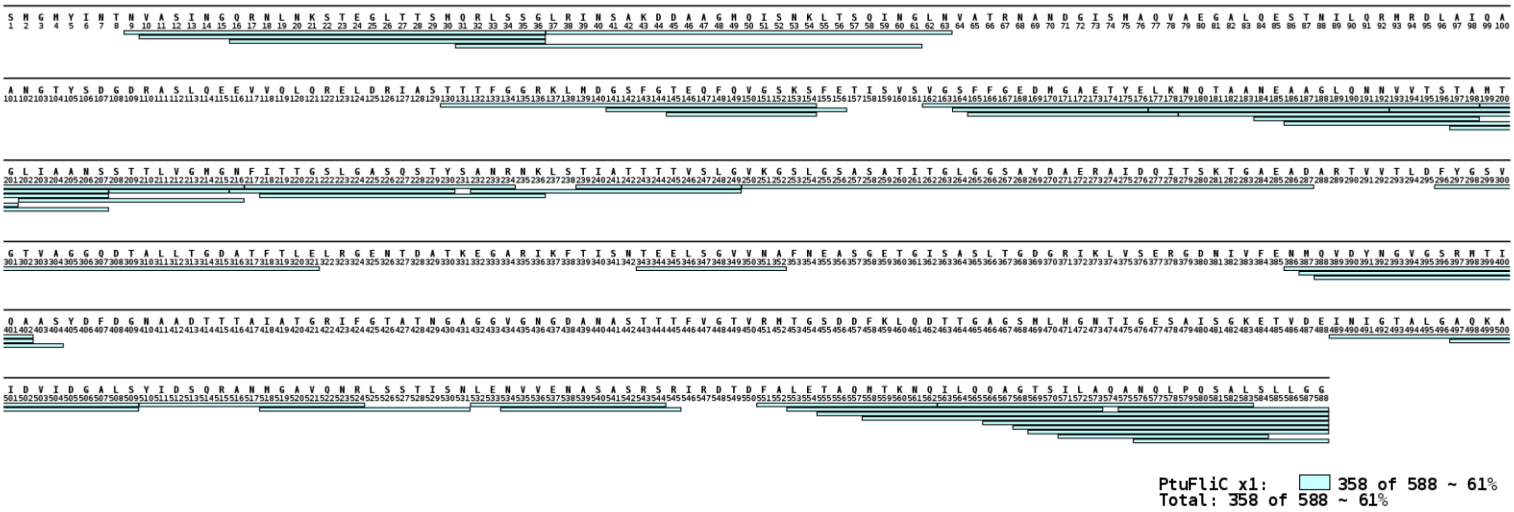


**B** Identified cleavage sites (P1-P1') in the structural flagellin FliC from *P. tunicata* by HgrFlaMP.

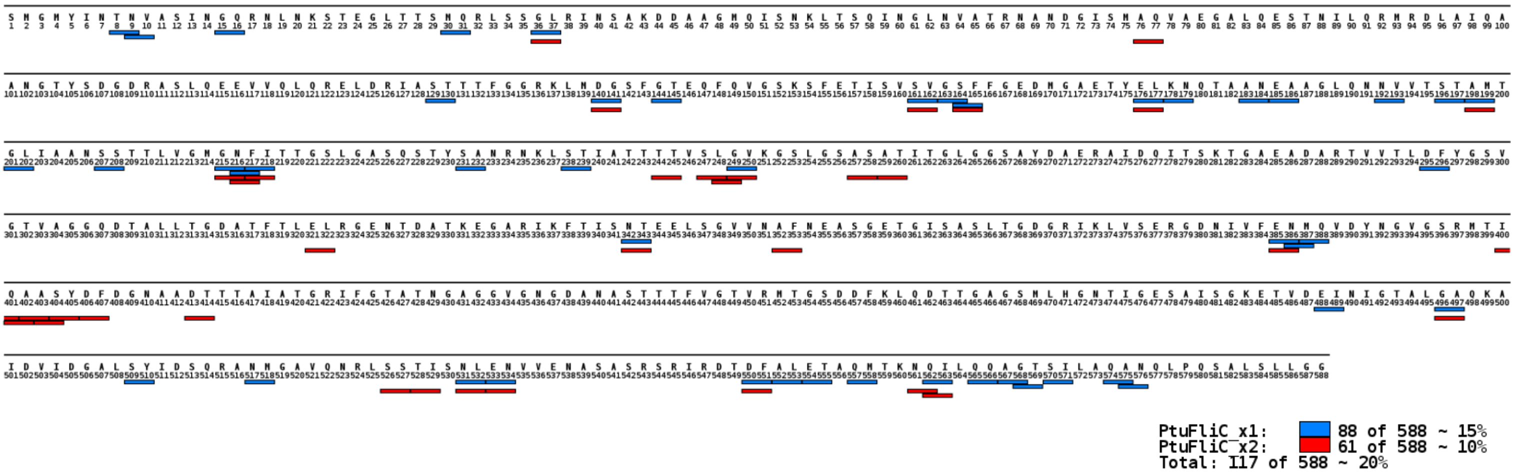


**Supplementary Figure 9. (A)** Sequence coverage of the structural flagellin FliC from *Pseudomalteromonas tunicata* after digestion with recombinant flagellinolysin from *Hylemonella gracilis* (HgrFlaMP-HVR). A protein coverage of 60.9% was obtained. **(B)** HgrFlaMP cleavage sites are indicated by colored bars spanning P1 to P1'. A total of 66 non-redundant cleavage sites could be identified. Blue bars: 48 cleavage sites derived from the proteomic experiment shown above. Red bars: 35 HgrFlaMP-derived cleavage sites after additional trypsin-digestion to render otherwise too long peptides suitable for mass spectrometry. LC-MS/MS data were collected using a Q-Exactive Plus mass spectrometer (Thermo Scientific), peptide-spectrum matches identified with X!Tandem (Craig and Beavis 2004), and validated with PeptideShaker (Vaudel et al. 2015) at a false discovery rate of 1%. Figures were created using the web-tool Draw Map within the MS Tools collection (Kavan and Man 2011).

**A** Sequence coverage of the proteolytic flagellin PtuFlaMP from *Pseudoalteromonas tunicata* by HgrFlaMP.

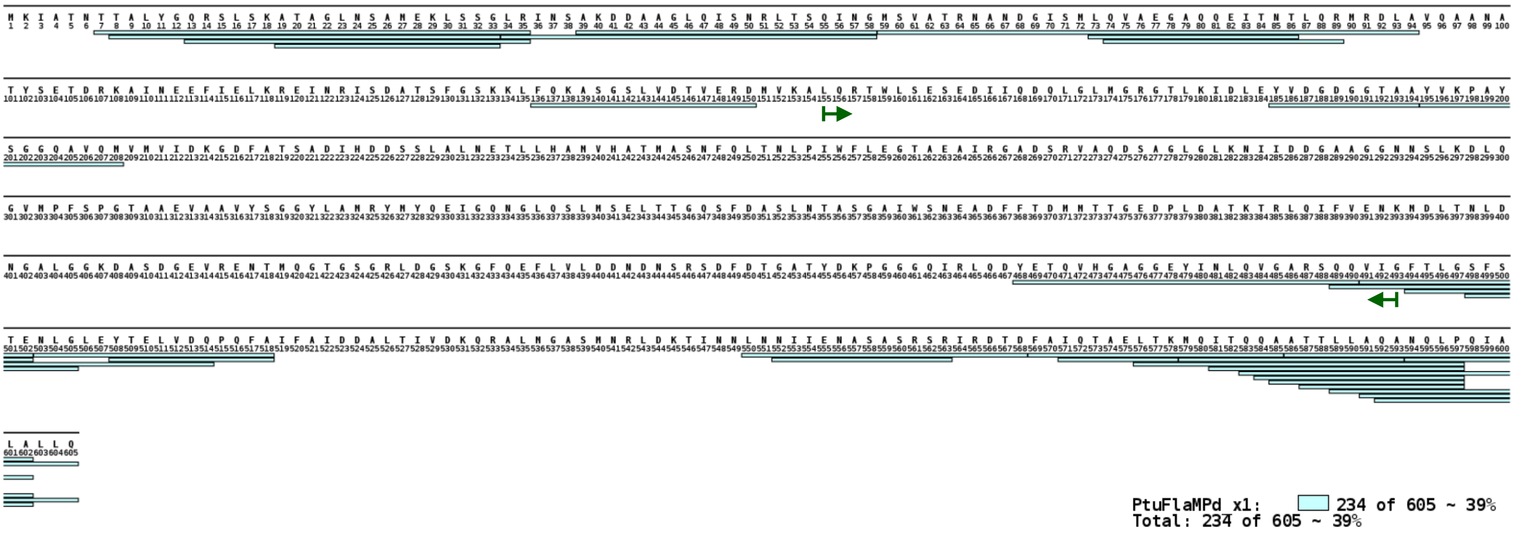


**B** Identified cleavage sites (P1-P1') in the proteolytic flagellin PtuFlaMP from *P. tunicata* by HgrFlaMP.

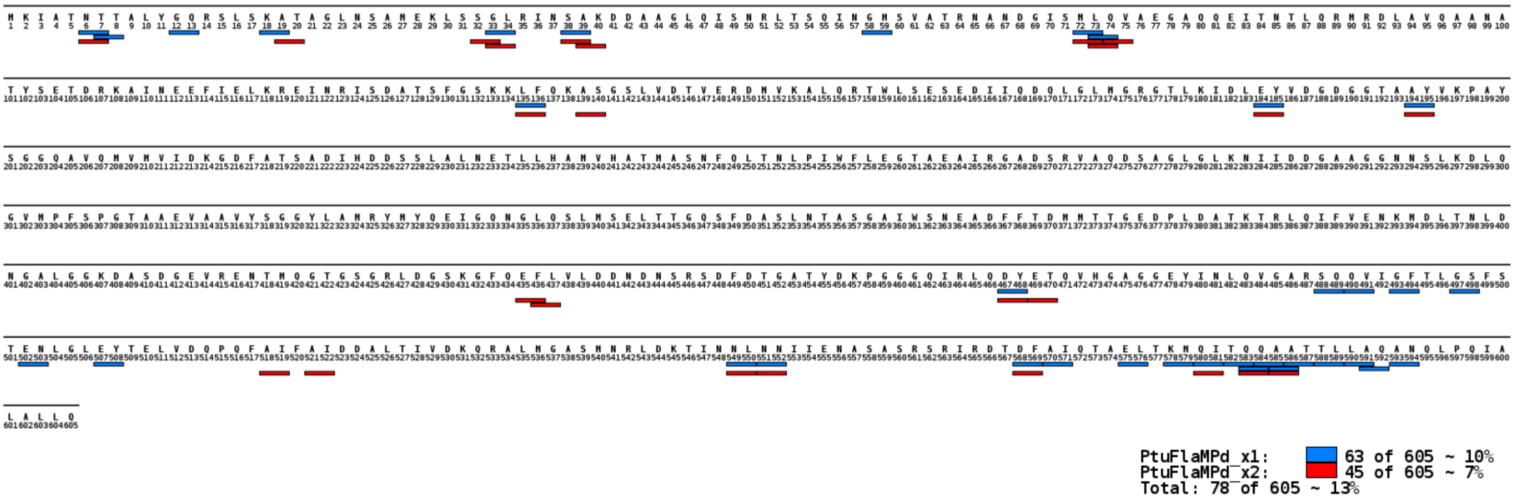


**Supplementary Figure 10. (A)** Sequence coverage of the HAxxH variant of the proteolytic flagellin from *P. tunicata* (PtuFlaMP HAxxH) after digestion with recombinant flagellinolysin from *Hylemonella gracilis* (HgrFlaMP-HVR). A protein coverage of 38.7% was obtained. Intriguingly, identified peptides nearly exclusively mapped to the N- and C-terminal parts conserved in flagellins, and not to the central HVR domain (L155-G493; indicated by green arrows). **(B)** HgrFlaMP cleavage sites are indicated by colored bars spanning P1 to P1'. A total of 45 non-redundant cleavage sites could be identified. Blue bars: 35 cleavage sites derived from the proteomic experiment shown above. Red bars: 25 HgrFlaMP-derived cleavage sites after additional trypsin-digestion to render otherwise too long peptides suitable for mass spectrometry. LC-MS/MS data were collected using a Q-Exactive Plus mass spectrometer (Thermo Scientific), peptide-spectrum matches identified with X!Tandem (Craig and Beavis 2004), and validated with PeptideShaker (Vaudel et al. 2015) at a false discovery rate of 1%. Figures were created using the web-tool Draw Map within the MS Tools collection (Kavan and Man 2011).

## A Sequence alignment of the structural flagellins of *Hylemonella gracilis* and *Pseudoalteromonas tunicata*.

```
HgrFliC     MAAVINTNINSLSAQRYQGISDKSLTTAIQRLSSGLRINSAKDDAAGLAISERFTSQIRG    60
PtuFliC     MGMYINTNVASINGQRNLNKSTEGLTTSMQRLSSGLRINSAKDDAAGMQISNKLTSQING    60
            *.  ****: *:..**  . * :.***::******************: **:::****.*

HgrFliC     LNQAARNANDGVSLAQTTEGALKAASDILQRVRELAVQSANATNSASDRQALNSEVAQLV    120
PtuFliC     LNVATRNANDGISMAQVAEGALQESTNILQRMRDLAIQAANGTYSDGDRASLQEEVVQLQ    120
            ** *:******:*:**.:****: :::****:*:**:*:**.* * .** :*:.**.**

HgrFliC     SELDRISTSTEFNGSKLLDGTFGTAQFQIGANANQTIVAATANARTSVYGNNQGINTVAA    180
PtuFliC     RELDRIASTTTFGGRKLMDGSFGTEQFQVGSKSFETISVSVG----SFFGEDMGAETYEL    176
             *****:::* *.* **:**:*** ***:*::: :** .:..    *.:*:: * :*

HgrFliC     VQTVASGAT------------------------------W---GANGVLAETFTINAA--    205
PtuFliC     KNQTAANEAAGLQNNVVTSTAMTGLIAANSSTTLVGMGNFITTGSLGASQSTYSANRNKL    236
             : .*:. :                              :   *: *.  .*:: *

HgrFliC     ----------------AGSAA-IATVLSETAKSVATKVNQQTSVTGVTAEARTDAQLSFA    248
PtuFliC     STIATTTTVSLGVKGSLGSASATITGLGGSAYDAERAIDQITSKTGAEADARTVVTLDFY    296
                             ***:   * *. :* ..   ::* ** **. *:*** . *.*

HgrFliC     ----------------ATGAYTLNLRSDNGNN---PESITFNLTDTSNTPSGLAAAISAF    289
PtuFliC     GSVGTVAGGQDTALLTGDATFTLELRGENTDATKEGARIKFTISN----TEELSGVVNAF    352
                            . .::**:**.:* :       *.*.:::     . *:..:.**

HgrFliC     NDKASKTGVTASLNAAGTGIILTNSTGNDIMVSDTSVINAGAV---TVNKMKTDTSGA--    344
PtuFliC     NEASGETGISASLTGDGRIKL-VSERGDNIVFENMQVDYNGVGSRMTIQAASYDFDGNAA    411
            *: :.:**::***.. *   : ... *::*:..: .*   *.    *::  . * .*

HgrFliC     -----LASVSTMT---------LAADTNAQNSIVSGYLTFDSNKSFVLSHAAT--VAASN    388
PtuFliC     DTTTAIATGRIFGTATNGAGGVGNGDANASTTTFVGTVRMTGSDDFKLQDTTGAGSMLHG    471
                 :*:   :            .*:**..: . * : : ....* *..::       .

HgrFliC     AYIGSDTSSTLHKVADLDVTSVIKANDAIKTVDSALAFISGERAKLGALQSRFDSTINNL    448
PtuFliC     NTIGESAISGKETVDEINIGTALGAQKAIDVIDGALSYIDSQRANMGAVQNRLSSTISNL    531
              **..: *  ..* :::: :.: *:.**..:*.**::*..:**::**:*.*:.***.**

HgrFliC     NITAENLTASRSRIQDADFASETAALSRAQILQQAGTAMIAQANQIPQGVLQLLQ-    503
PtuFliC     ENVVENASASRSRIRDTDFALETAQMTKNQILQQAGTSILAQANQLPQSALSLLGG    587
            : ..** :******:*:*** *** ::: ********:::*****:**..*.**
```

## B Sequence identity matrix of HgrFliC and PtuFliC.

| | Identities | Positives | Gaps |
|---|---|---|---|
| Overall | 218/593 (37%) | 313/593 (53%) | 60/593 (10%) |
| N-term | 98/185 (53%) | 129/185 (69%) | 4/185 (2%) |
| HVR | 69/316 (22%) | 114/316 (36%) | 56/316 (17%) |
| C-term | 51/96 (53%) | 70/96 (72%) | 0/96 (0%) |

## C

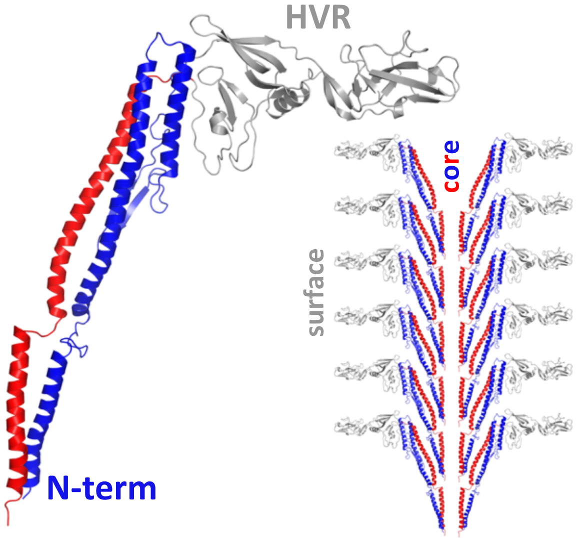


**Supplementary Figure 11. (A)** Protein sequence alignment and **(B)** sequence identity matrix of the structural flagellins from *Hylemonella gracilis* (HgrFliC; UniProt A0A4P6UHD3) and *Pseudoalteromonas tunicata* (PtuFliC; UniProt A4C6G1) using Clustal Omega (Madeira et al. 2019) and BLASTp (Altschul et al. 1990). **(C)** Representation of the structural flagellin FliC from *Salmonella typhimurium* (PDB entry 1uxu; Yonekura et al. 2003), and a schematic for its packing within a flagellar filament. The highly conserved N- and C-terminal coiled-coil parts of flagellins, which build the core of the flagellar filament, are highlighted in blue and red, respectively, while the central surface located hypervariable region is depicted in grey.

# SDS-PAGE images used for figure preparations.

# Figure 2A

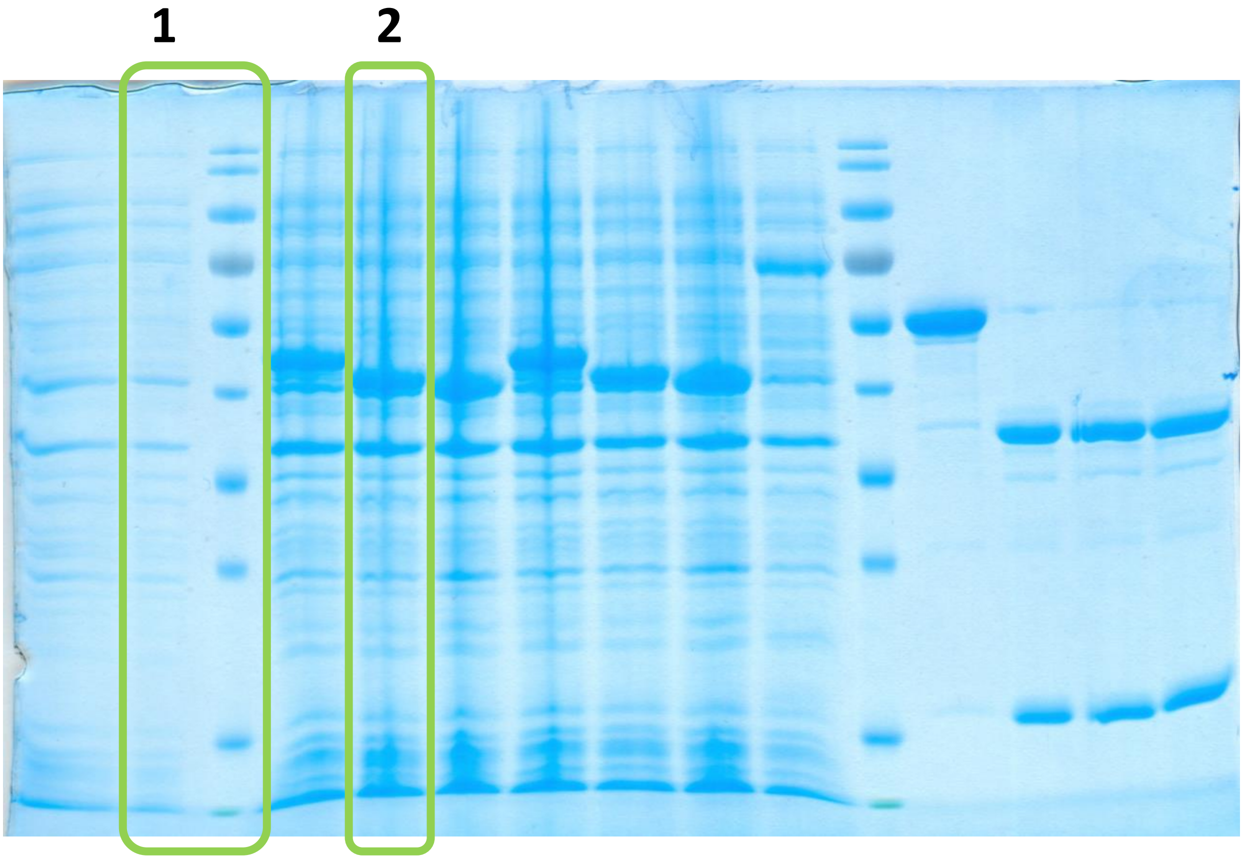


# Figure 2B

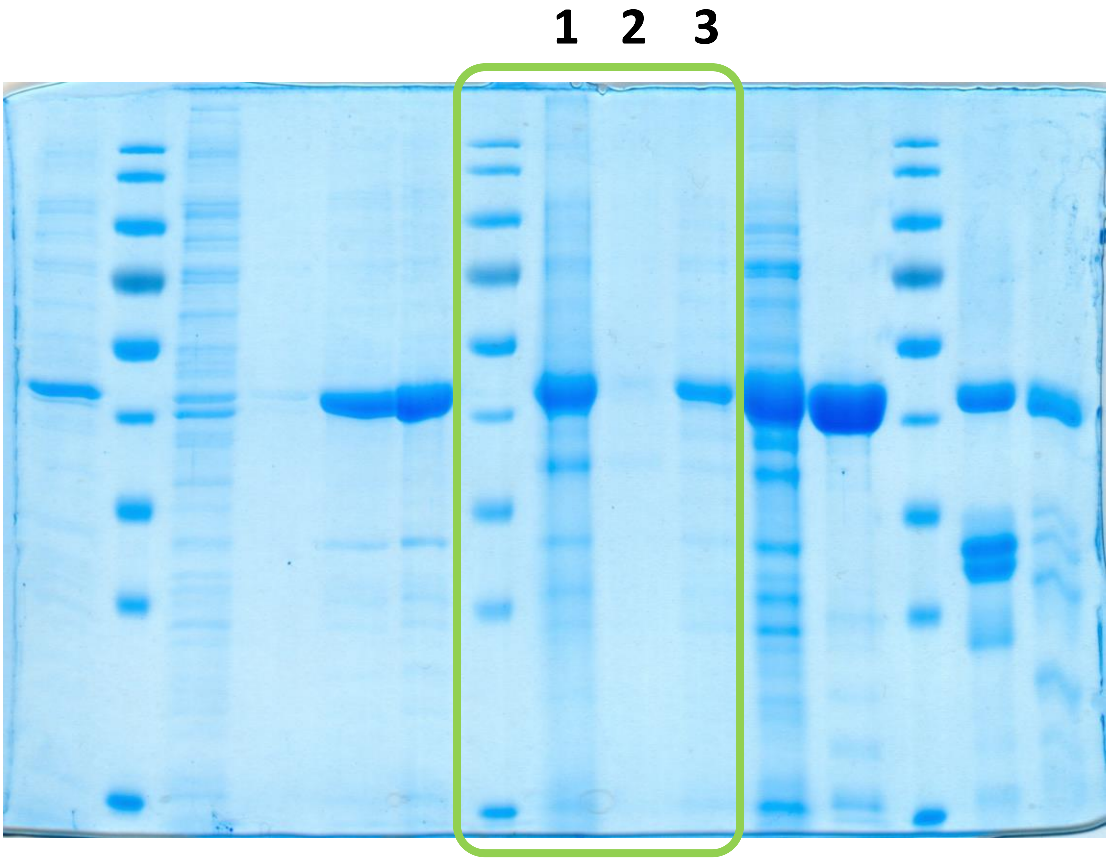

# Figure 2B

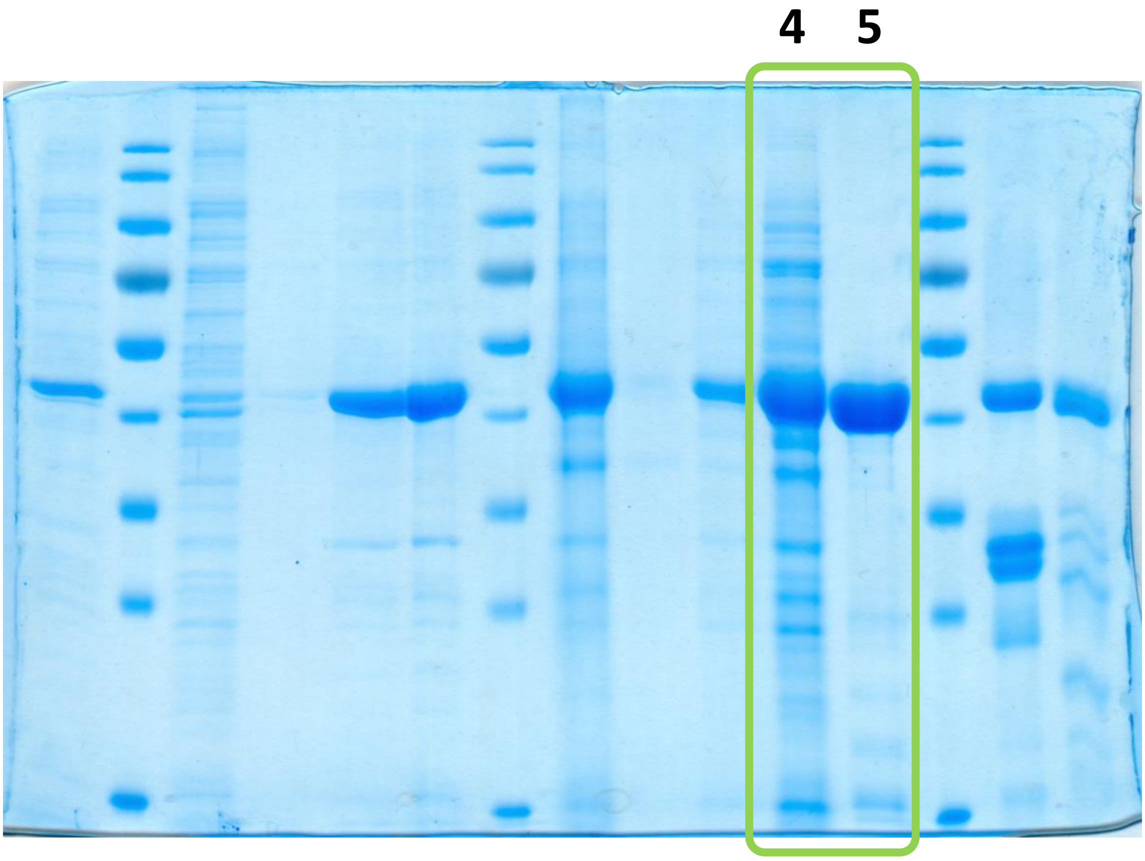


# Figure 2C

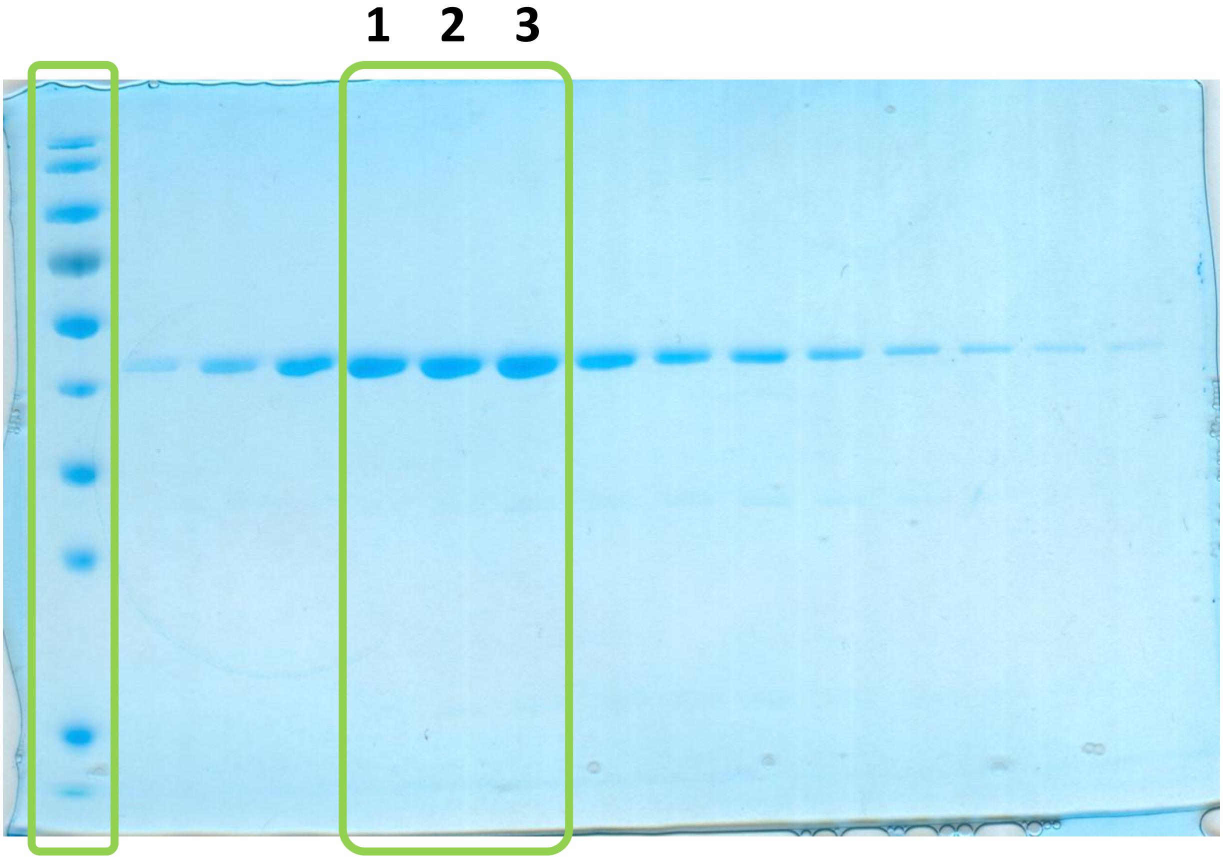

# Figure 2D

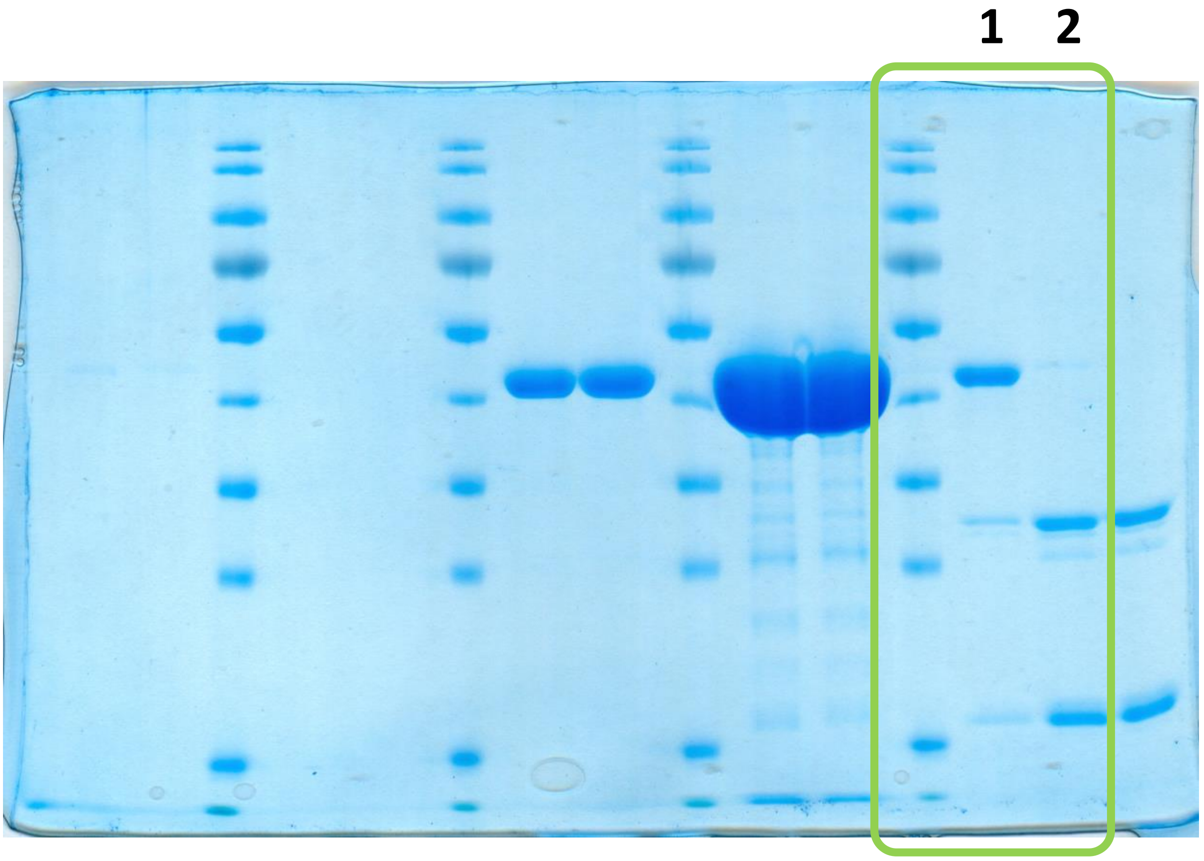


# Figure 2E

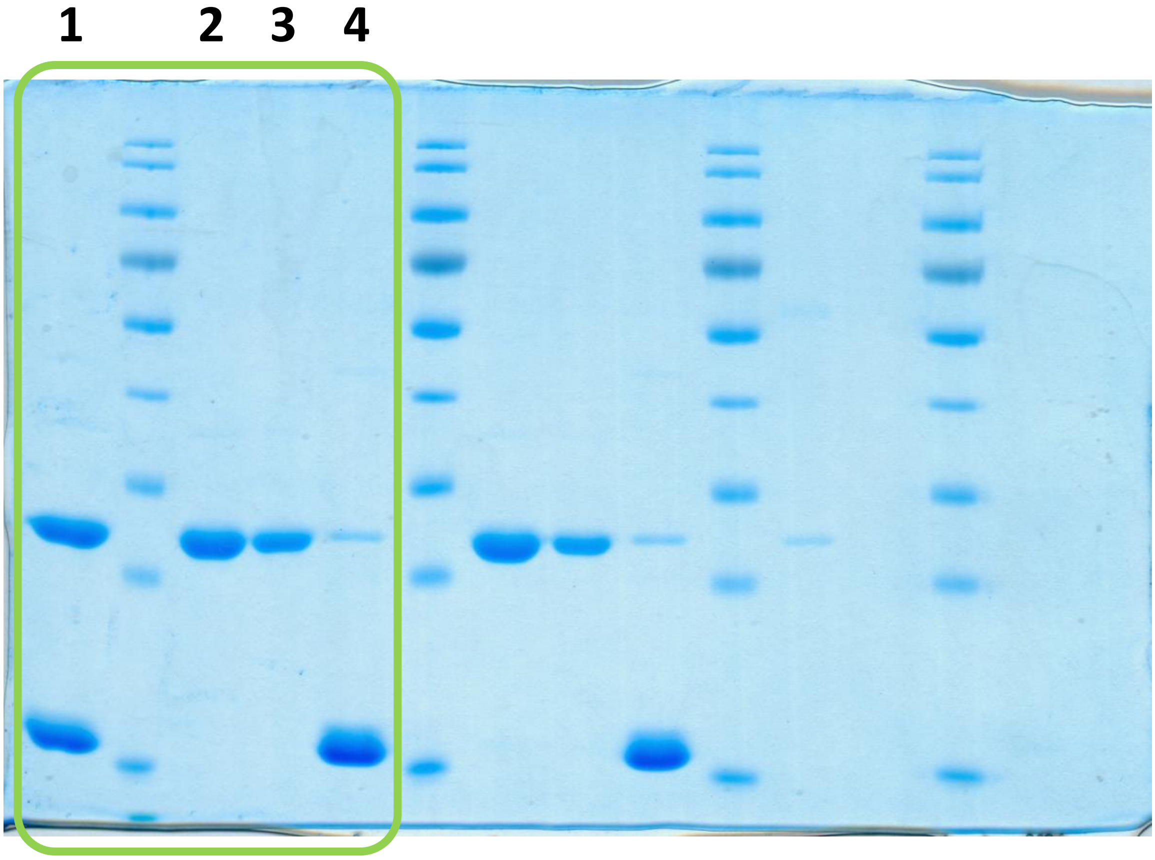

# Figure 2F

1

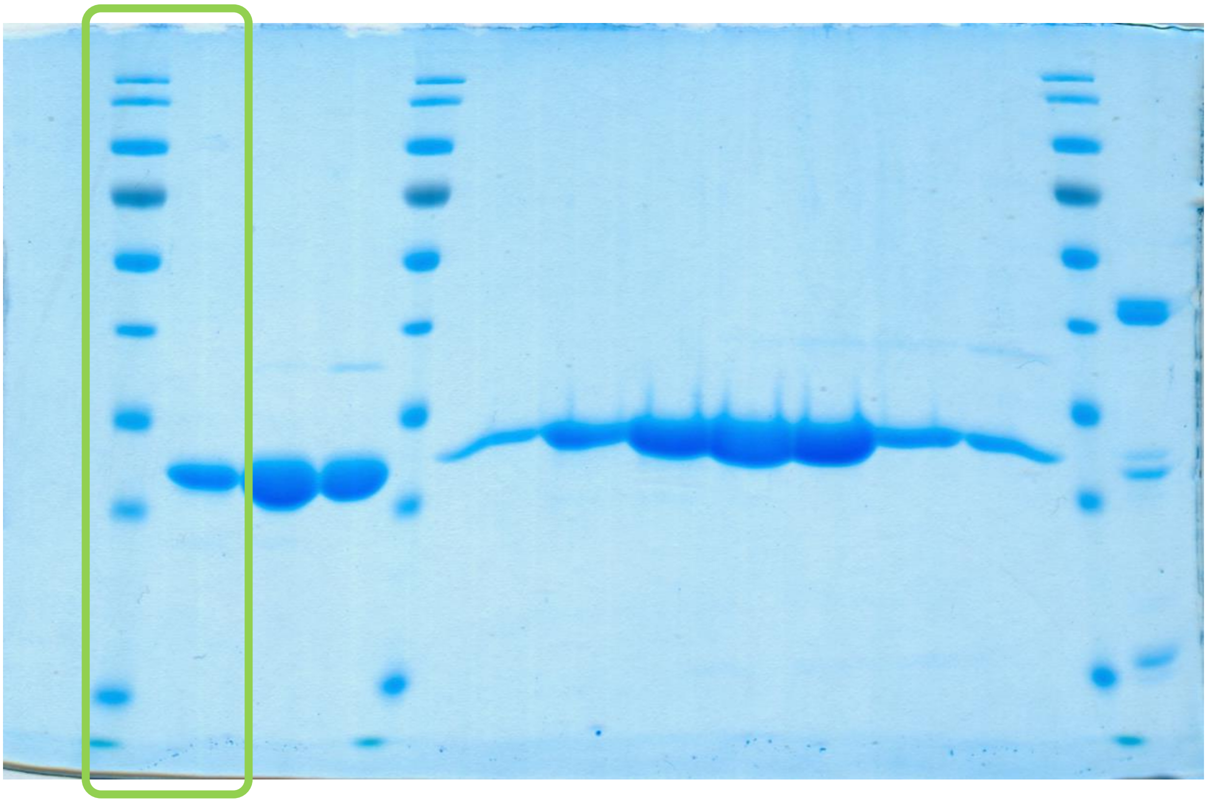

# Figure 3A

1

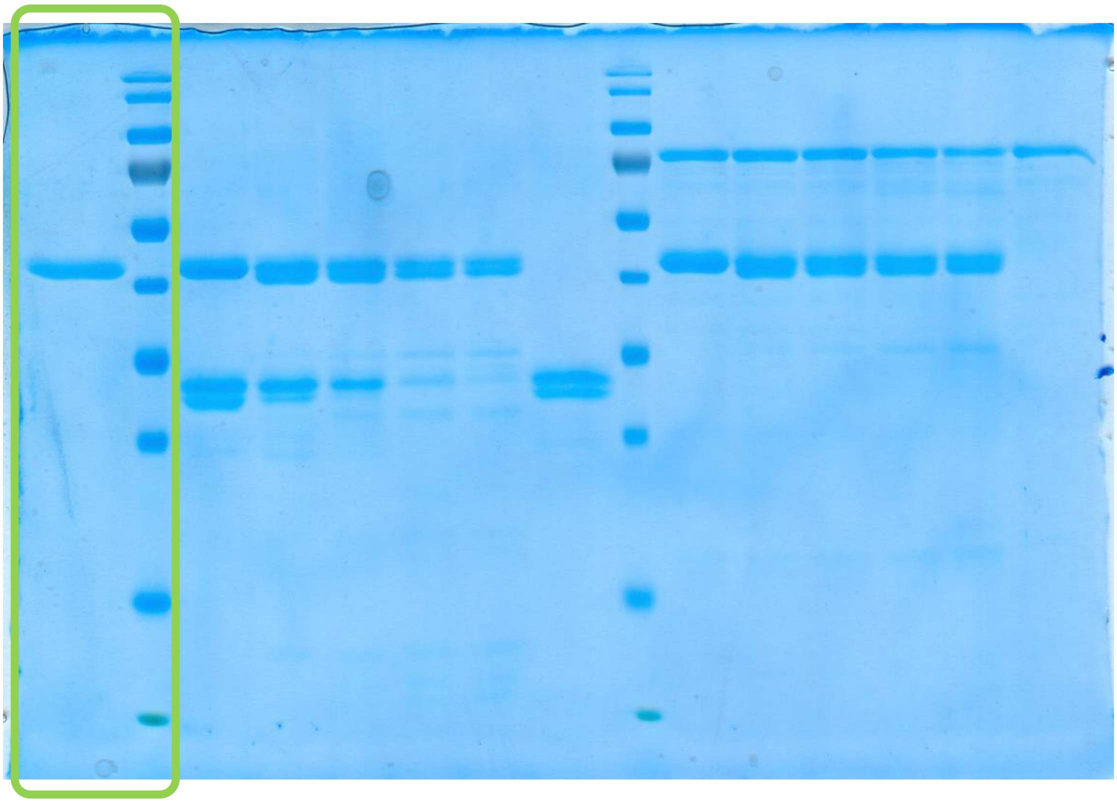

# Figure 3A

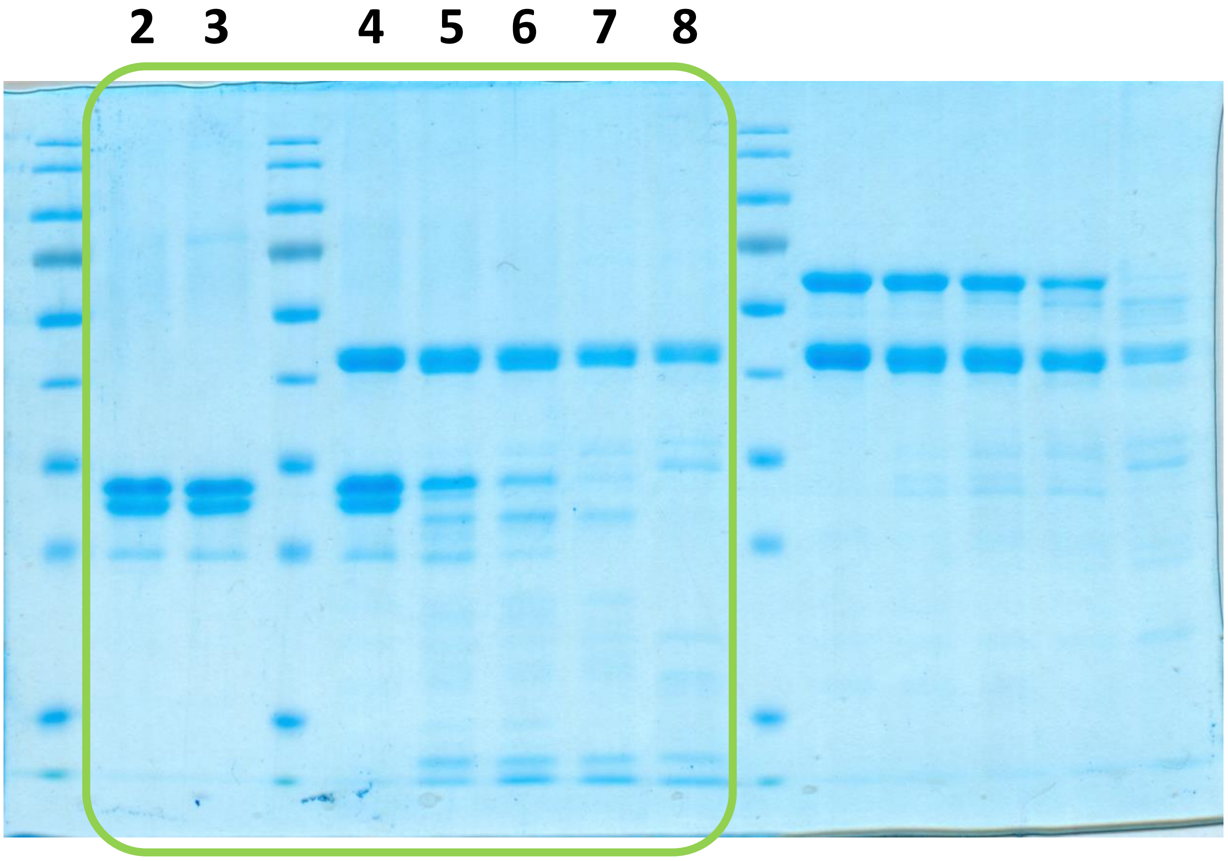


# Figure 3B

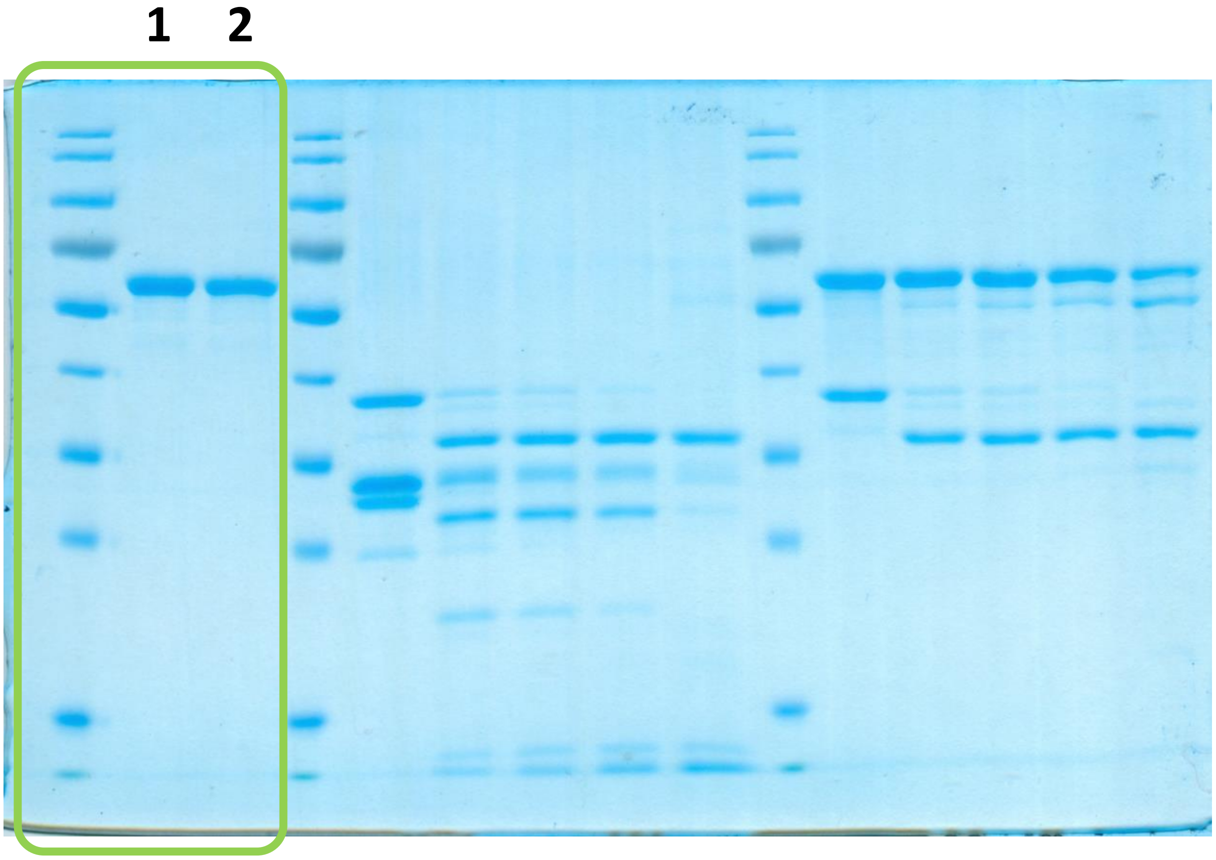

# Figure 3B

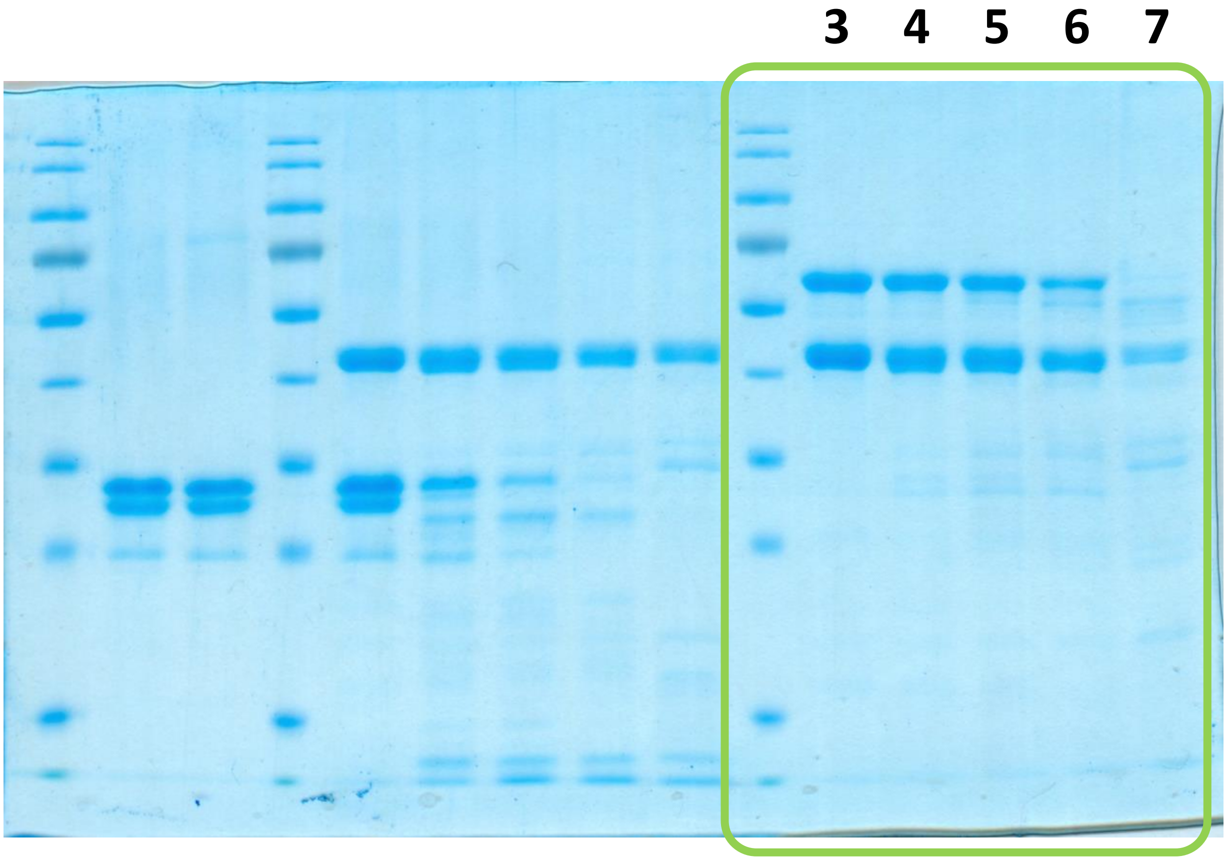


# Figure 3C

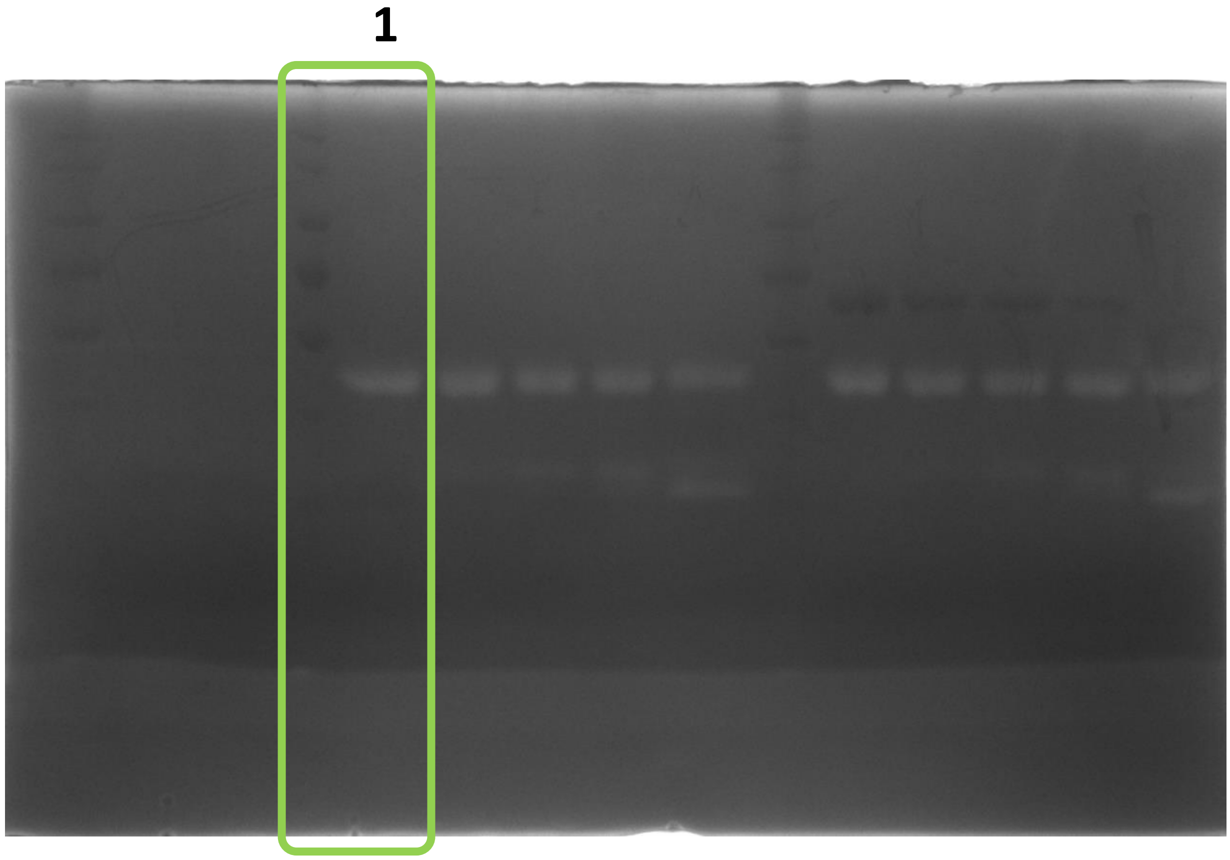

# Figure 4A

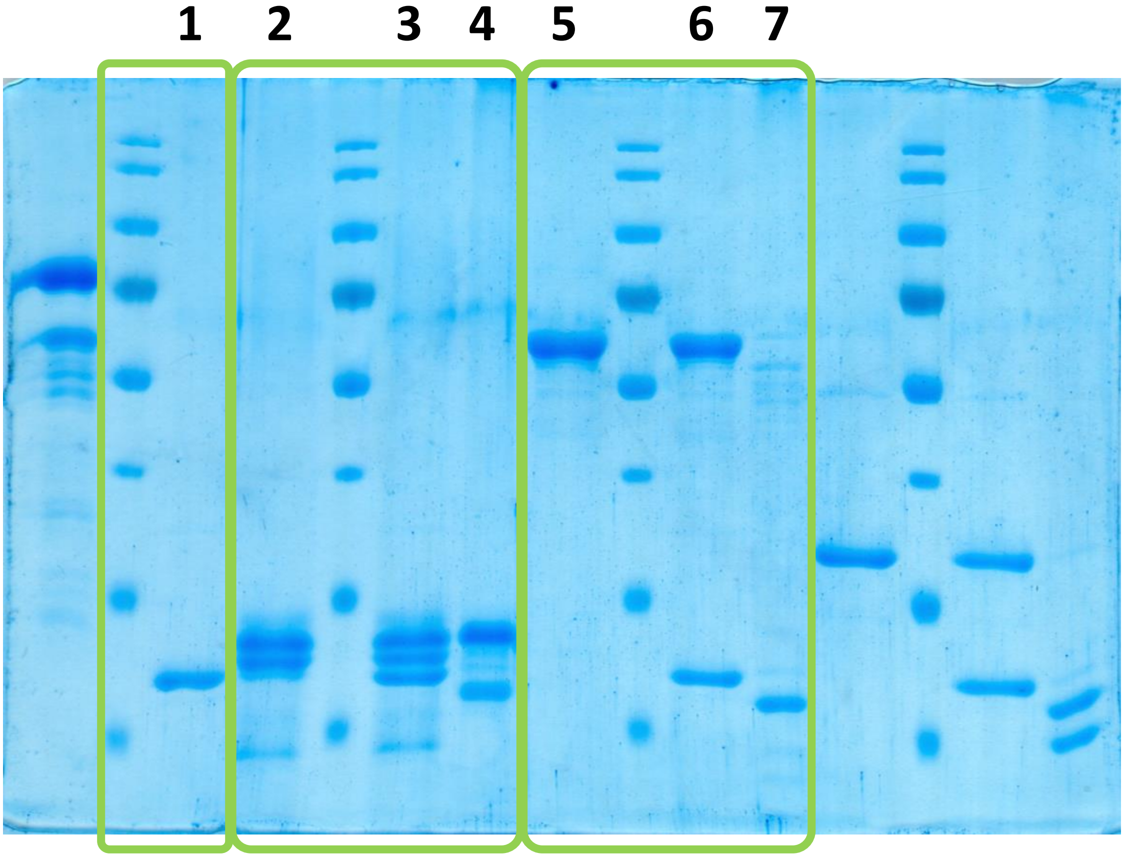


# Figure 4B

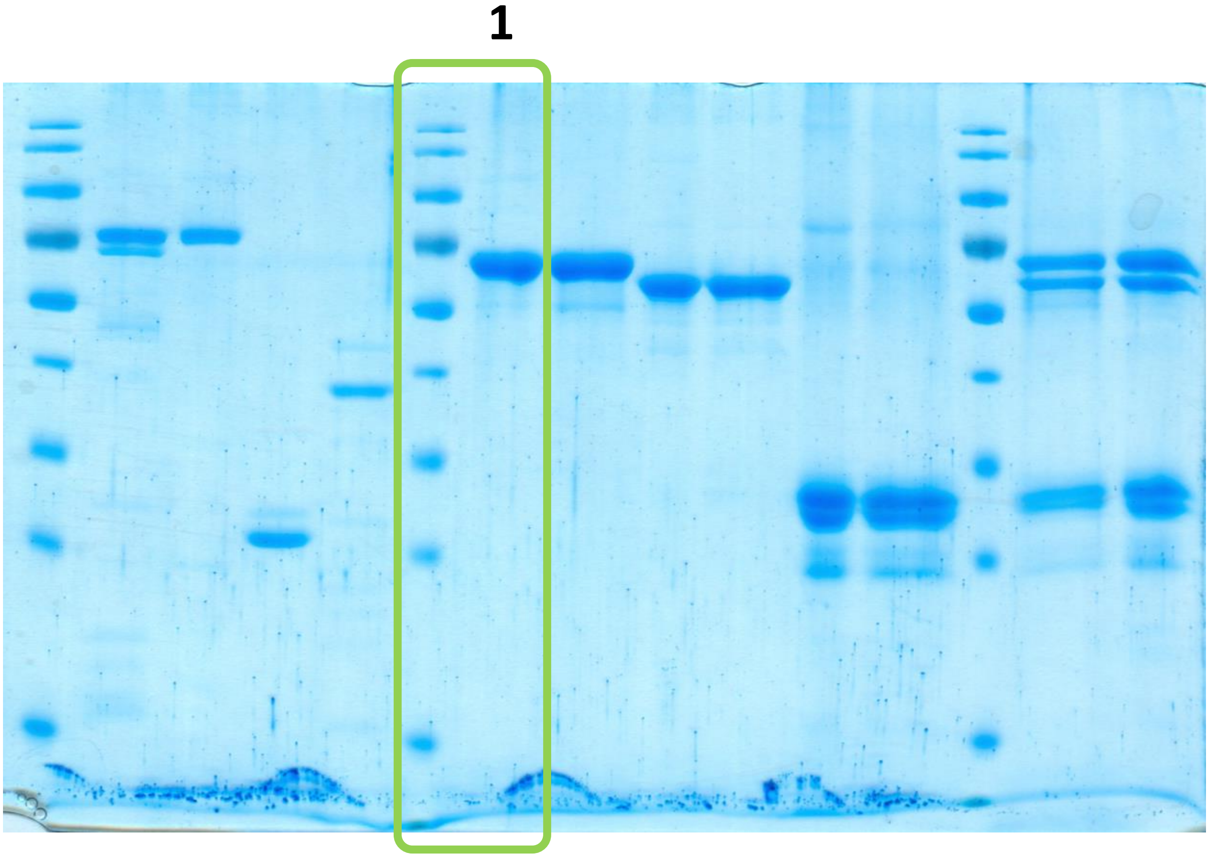

# Figure 4B

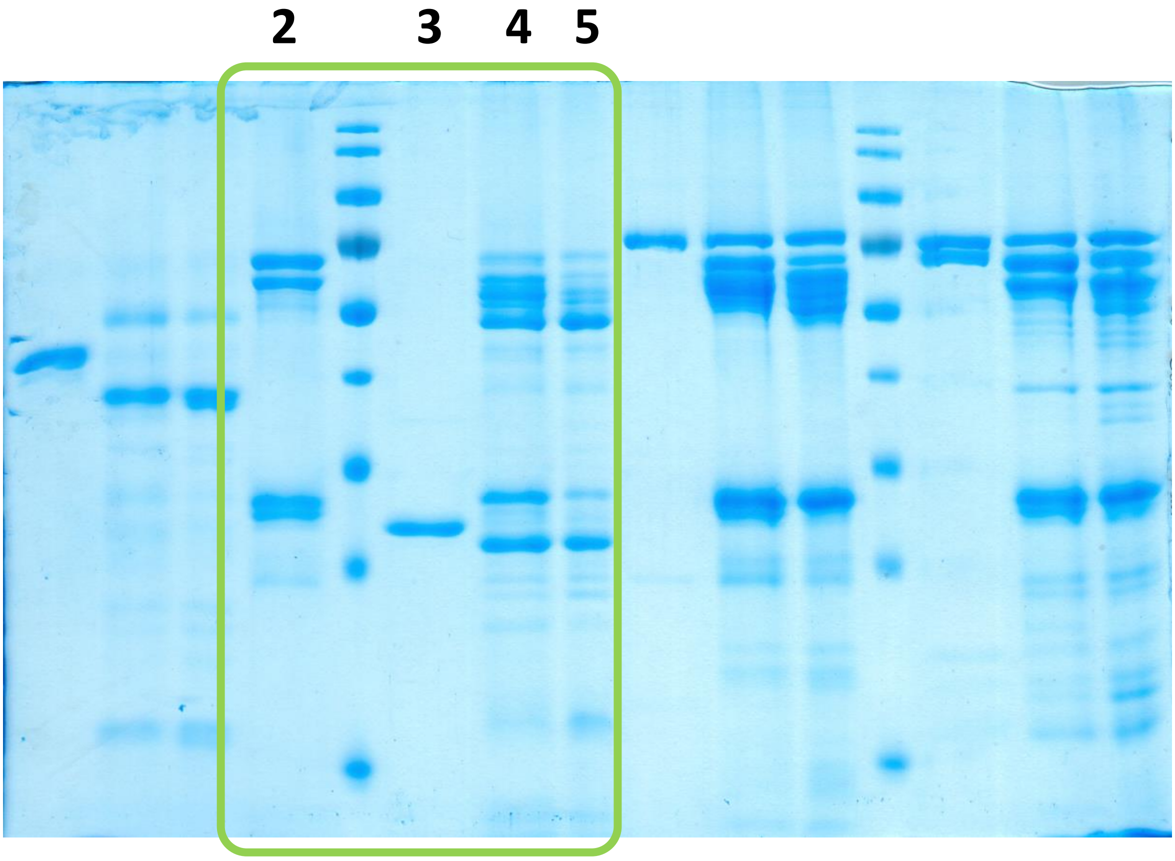


# Figure 4B

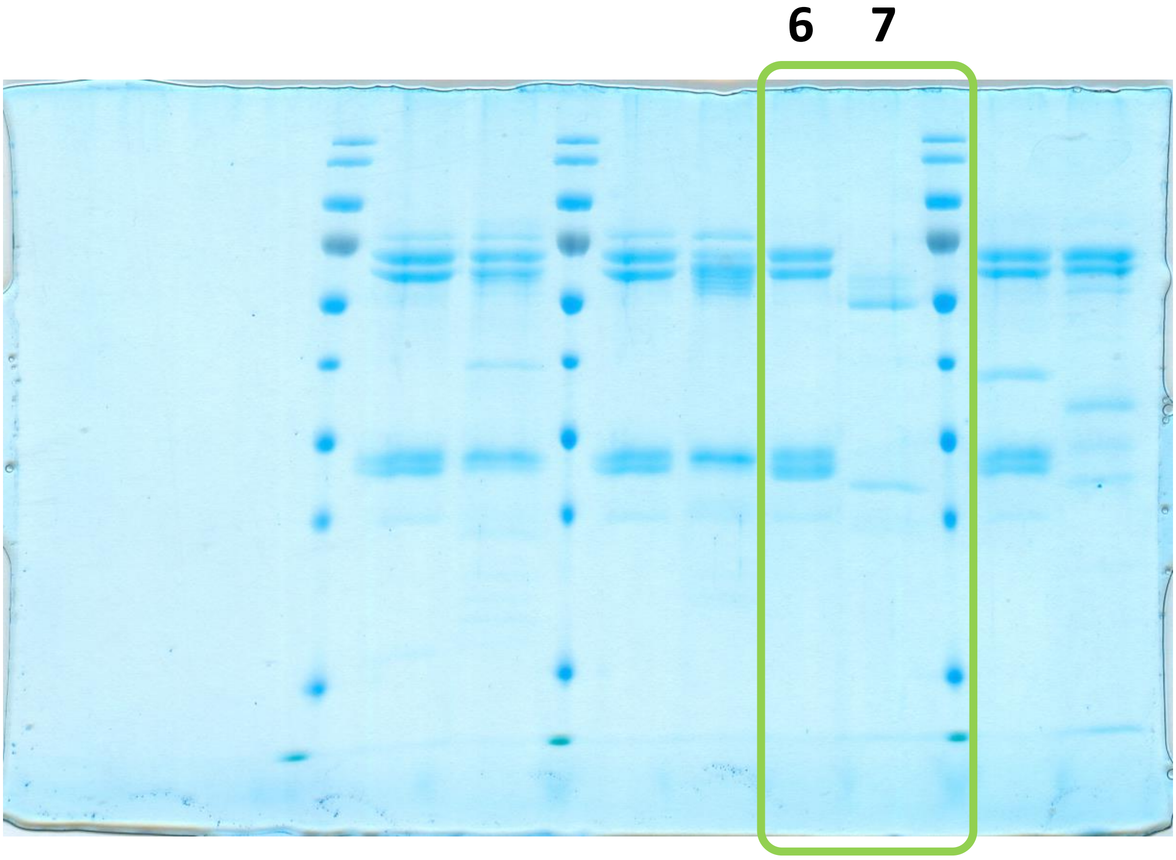

# Supplementary Figure 1A

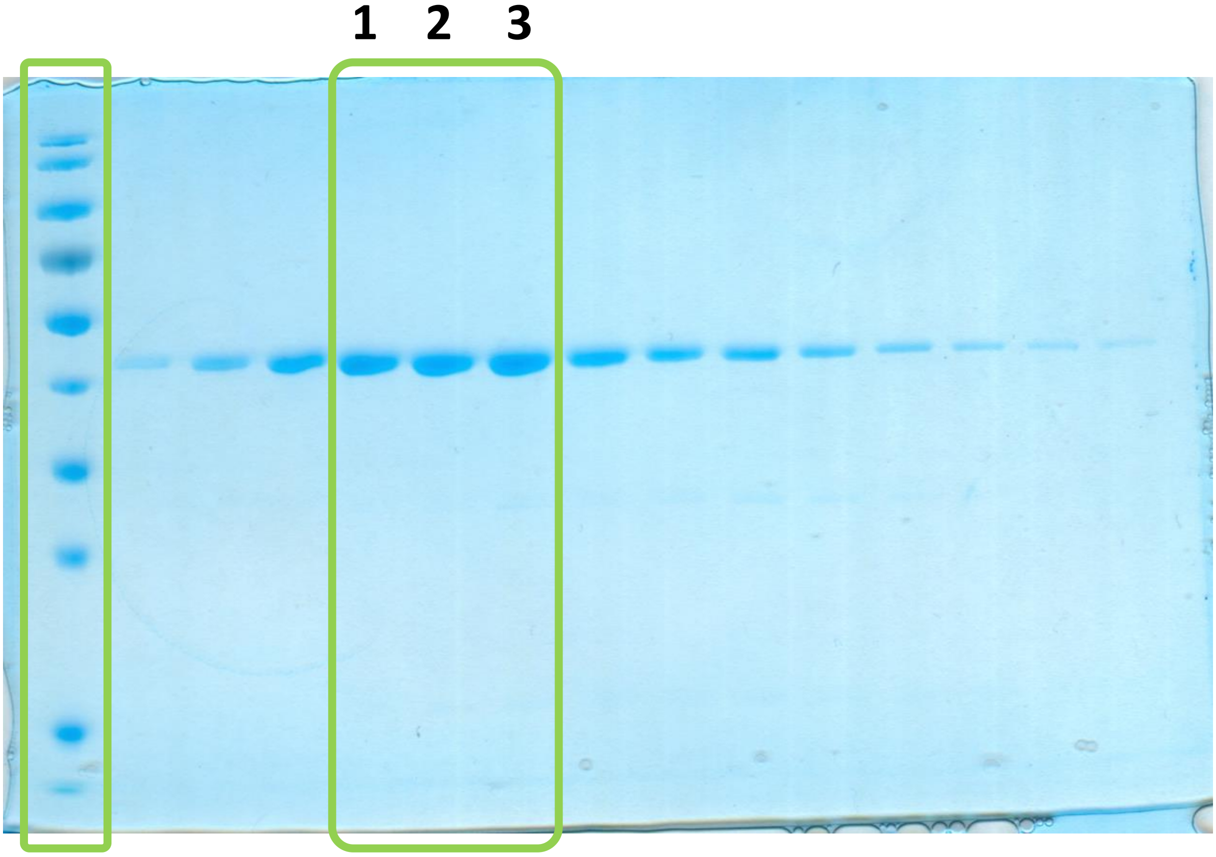


# Supplementary Figure 1B

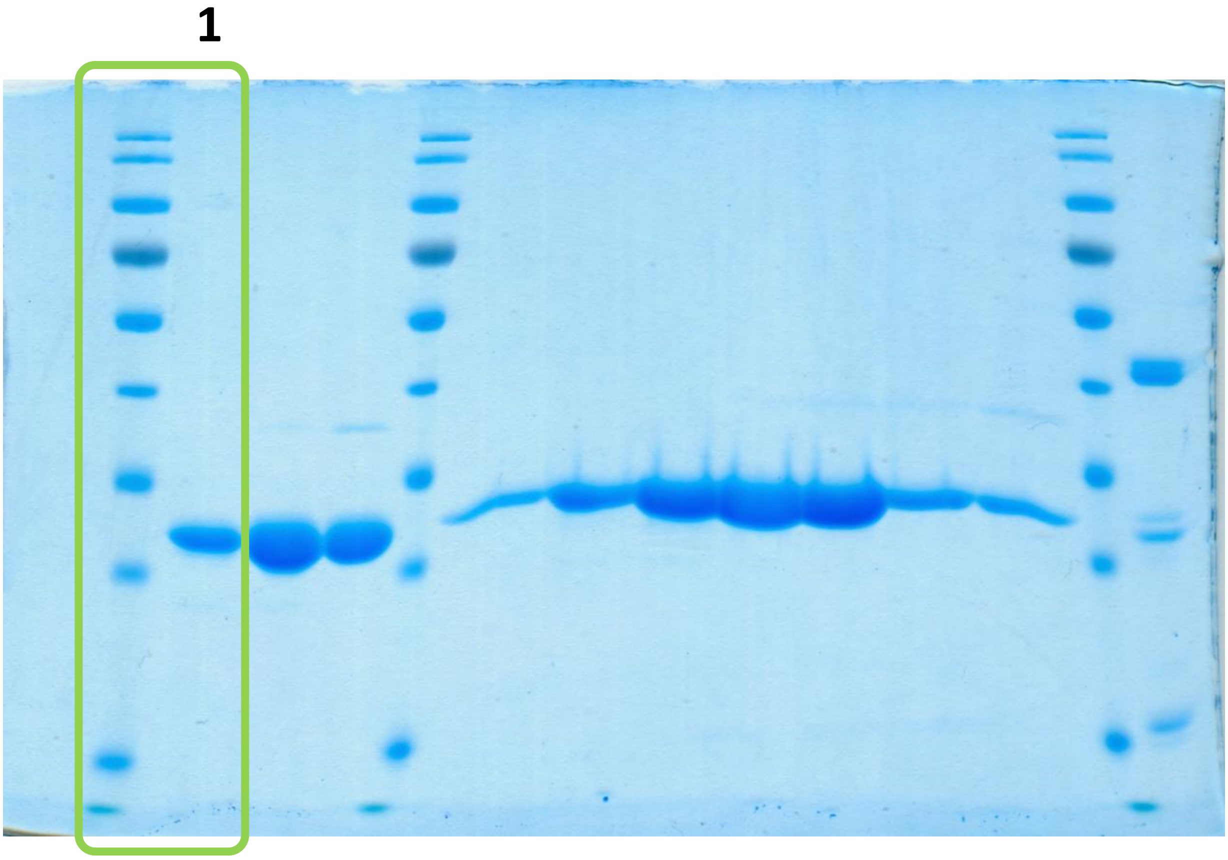

## Supplementary Figure 2A

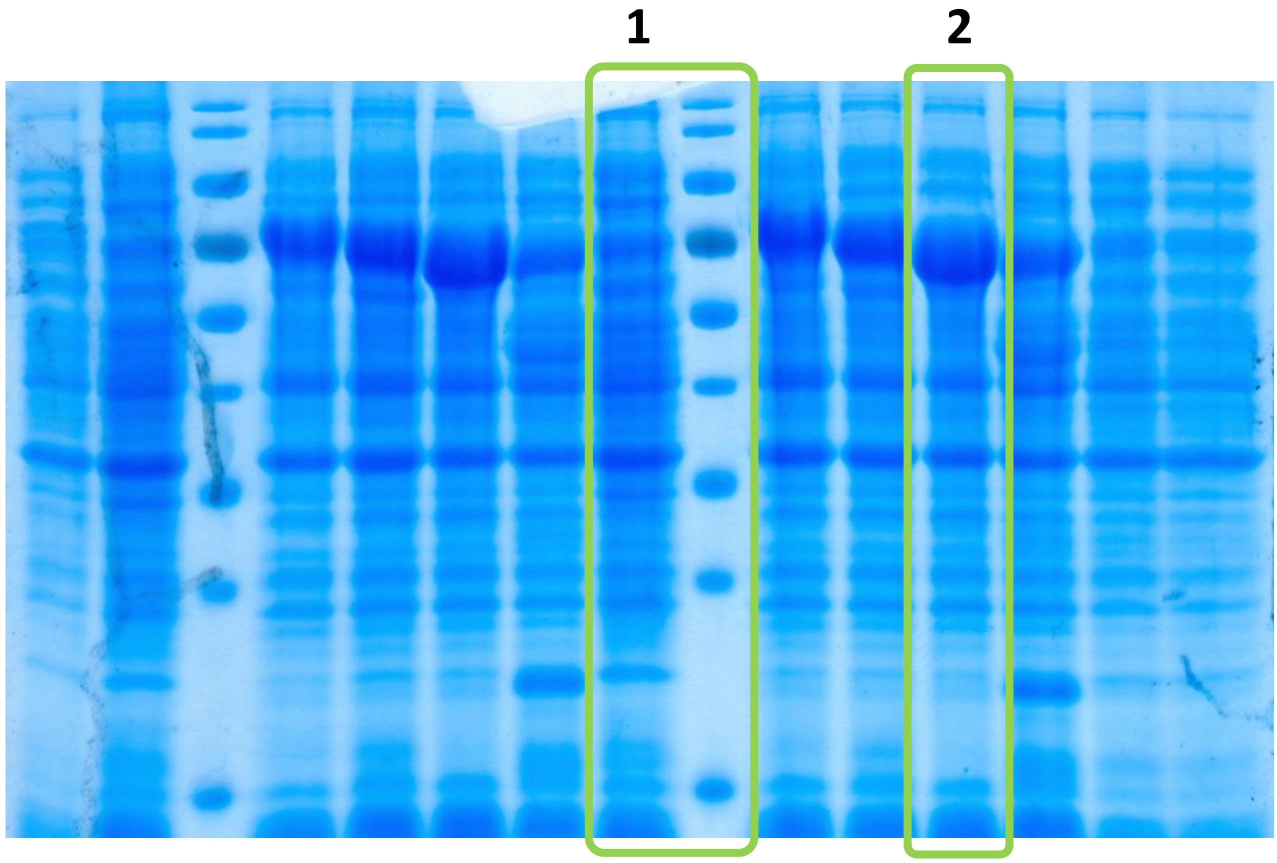


## Supplementary Figure 2B

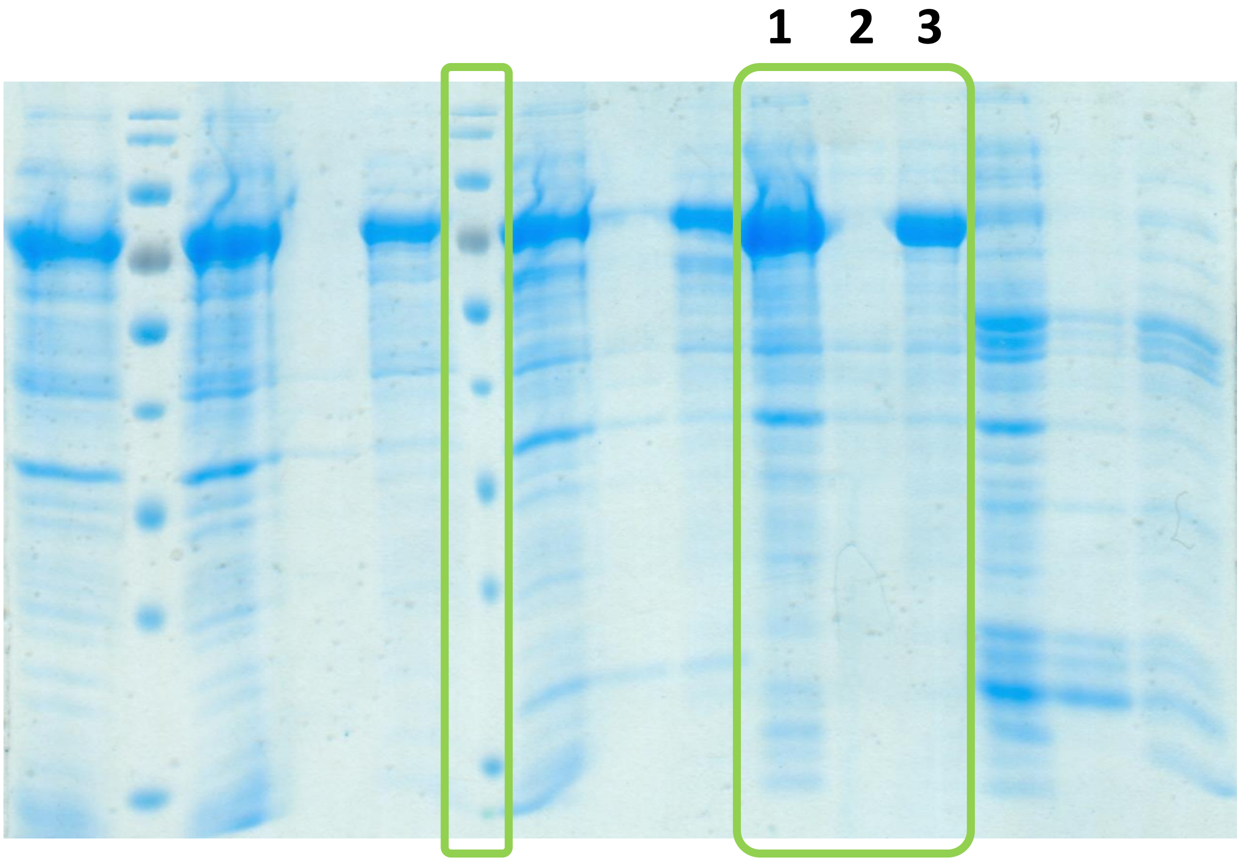

## Supplementary Figure 2B

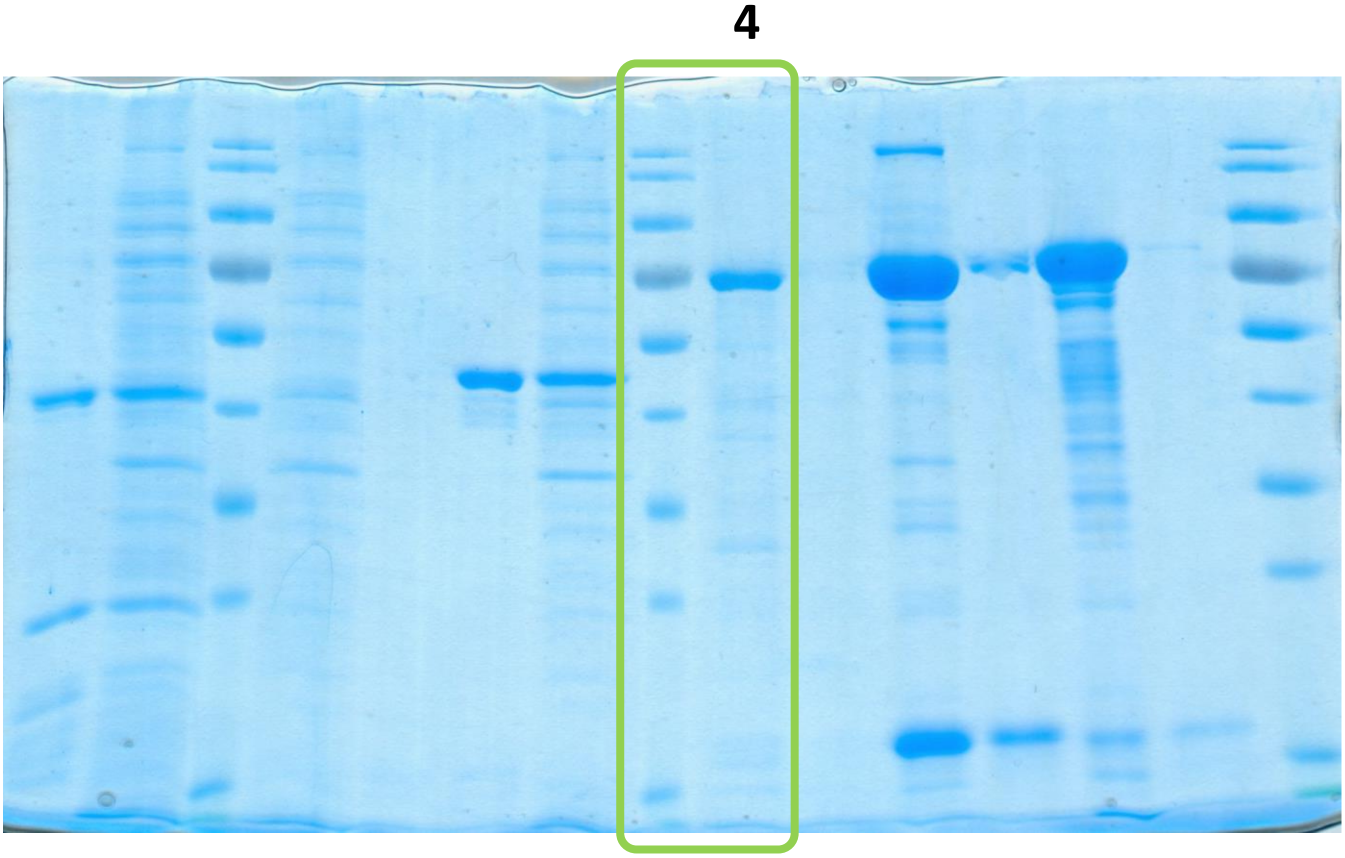


## Supplementary Figure 2C

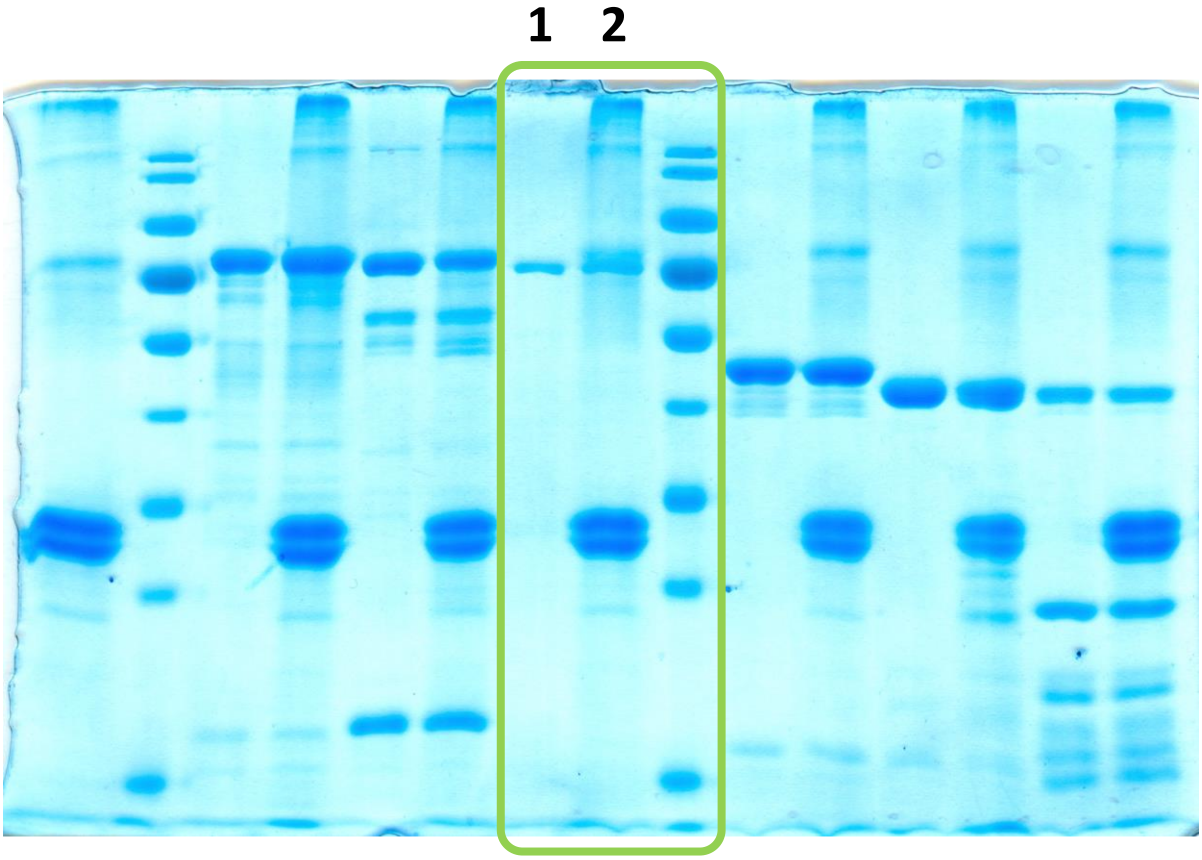

# Supplementary Figure 2C

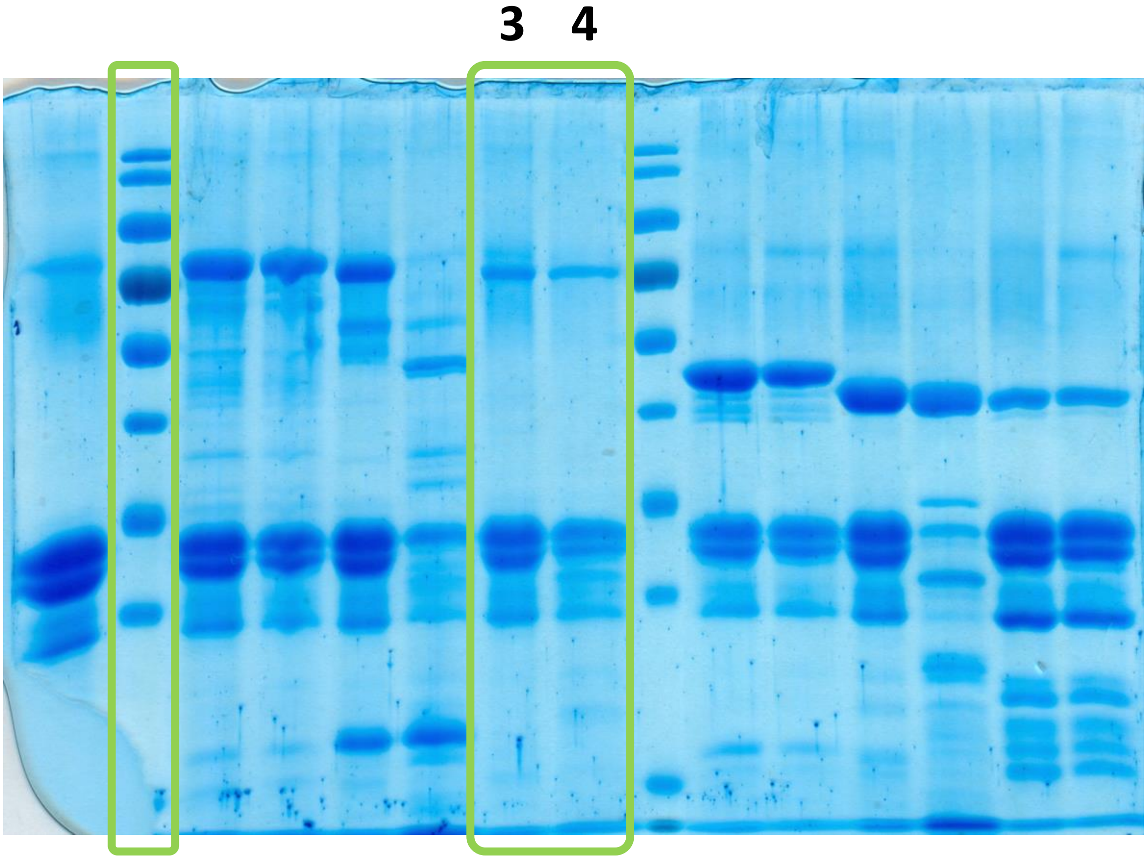


# Supplementary Figure 7A

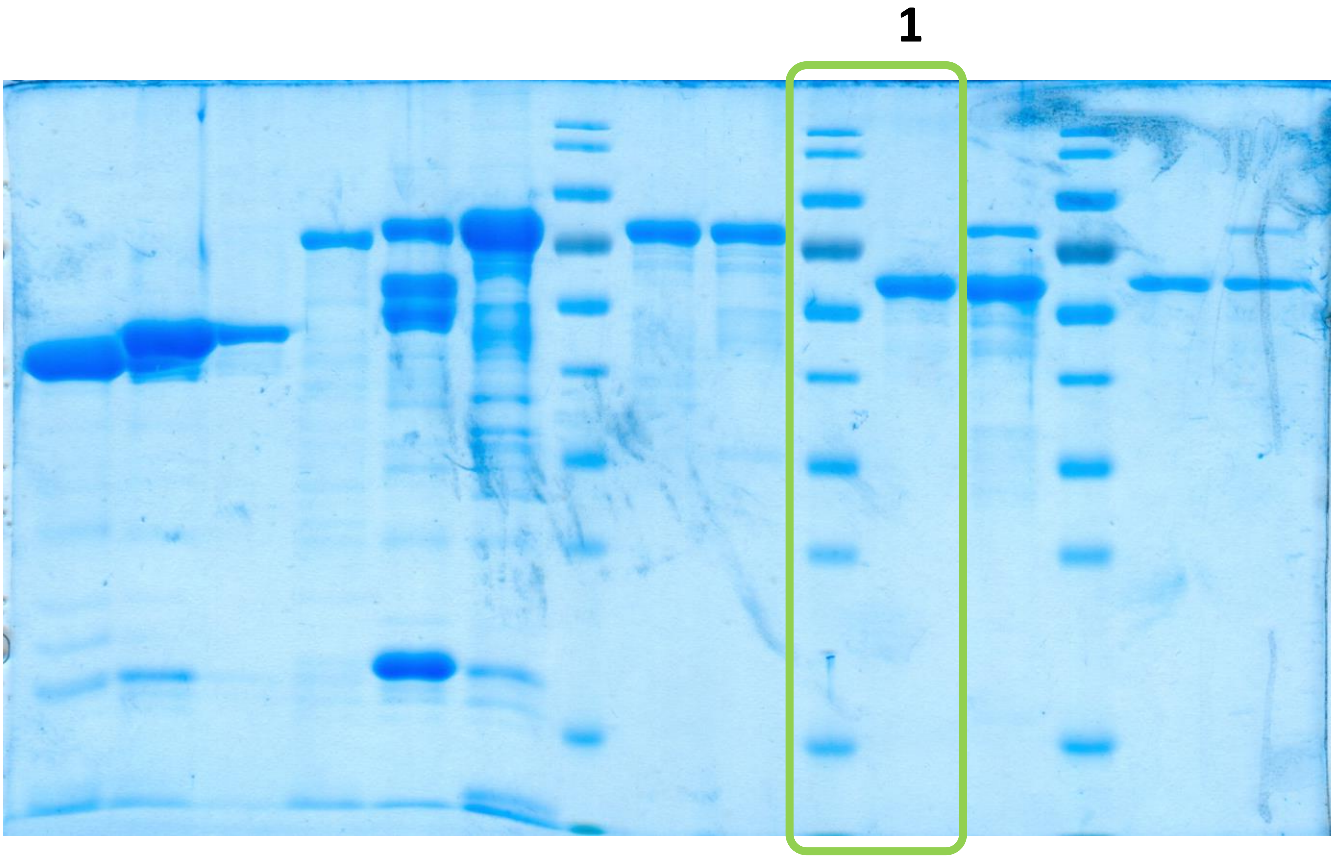

## Supplementary Figure 7A

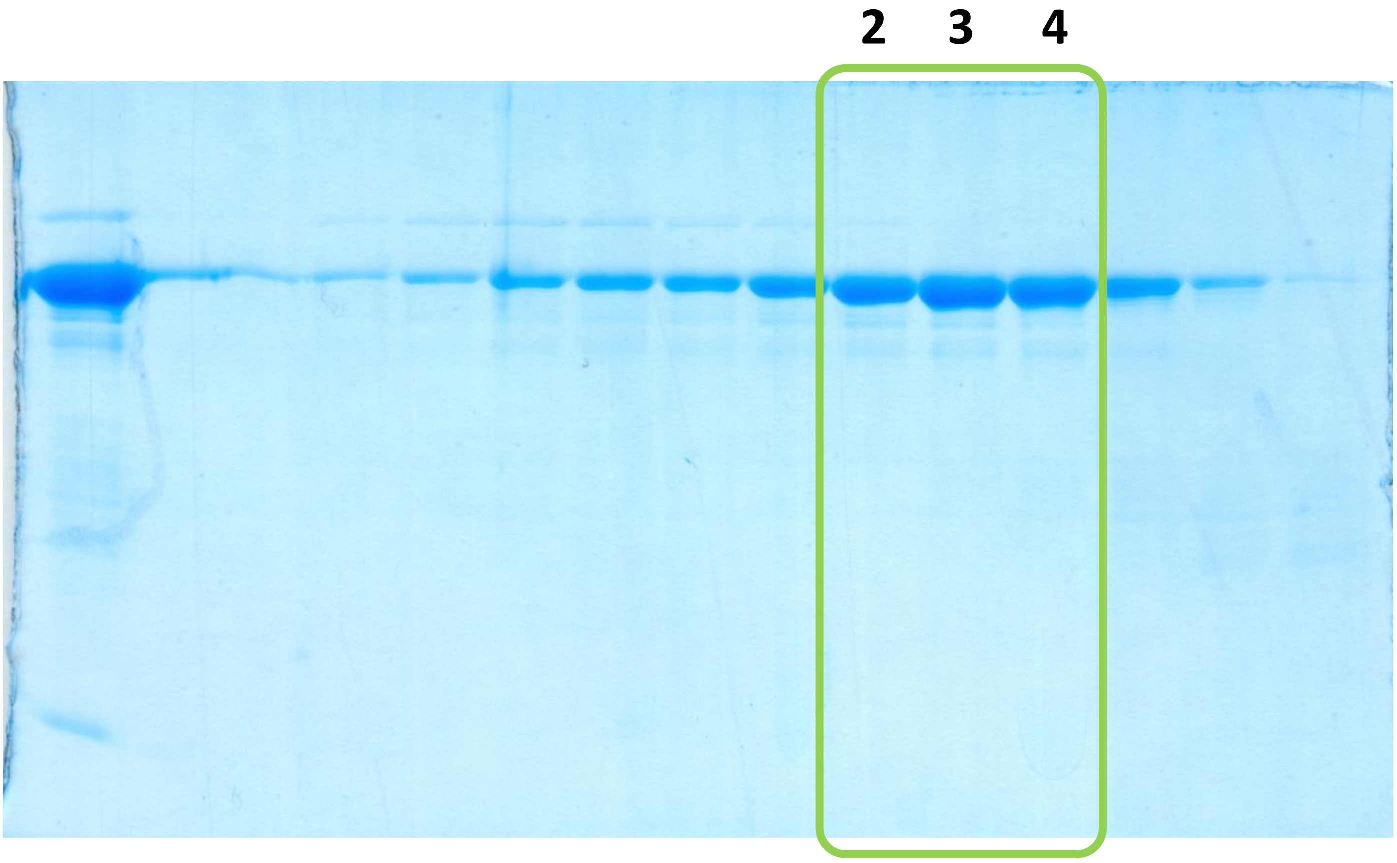


## Supplementary Figure 7B

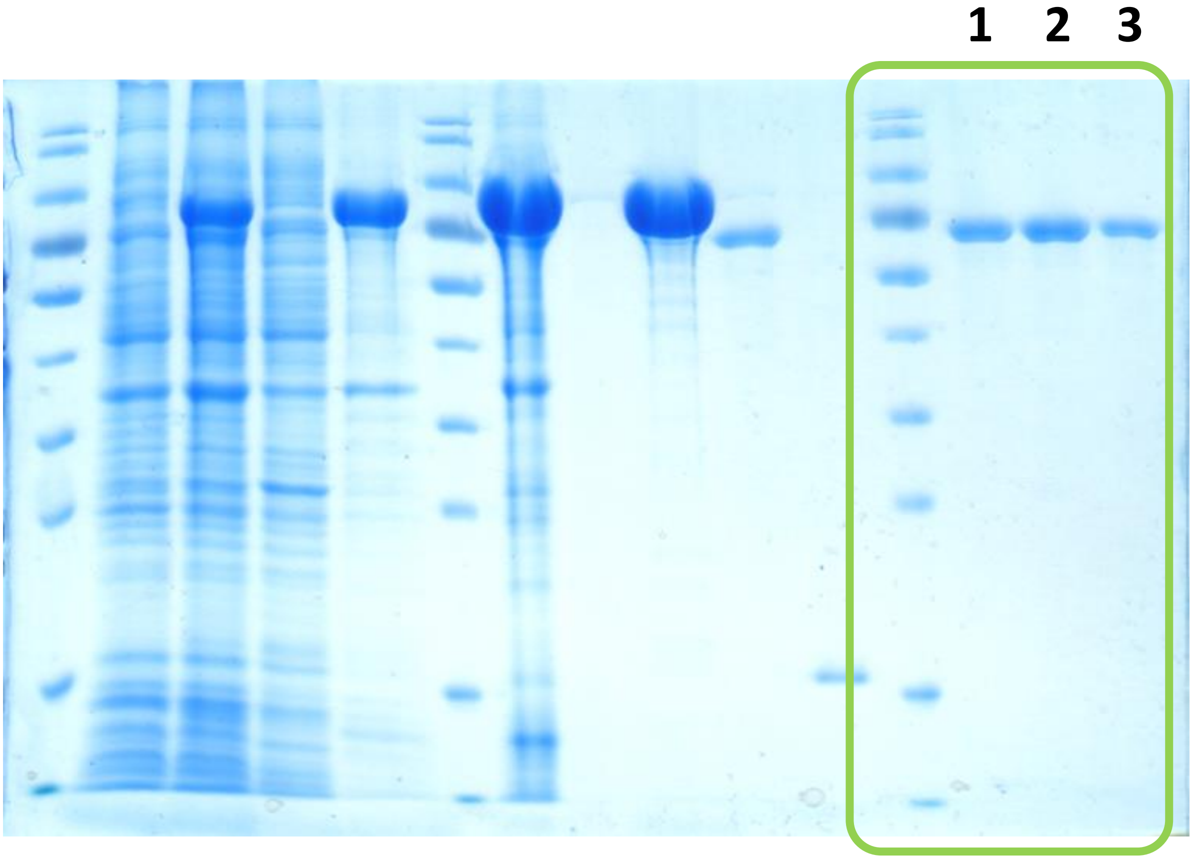

Supplement: Supplementary file 1 — Supplementary Figures. [file 41598_2020_76010_MOESM1_ESM.pdf]
